# ESMO-ESTRO consensus statements on the safety of combining radiotherapy with EGFR, ALK, or BRAF/MEK inhibitors

**DOI:** 10.1016/j.esmoop.2026.106076

**Published:** 2026-02-26

**Authors:** E.S.M. van Aken, S.M. O’Cathail, A.K. Gandhi, J. Bussink, L. Castelo-Branco, J.G. Eriksen, G. Argilés, C.T. Hiley, V. Atkinson, J. Kaźmierska, A. Calles, K. Konopa, E. Le Rhun, F. McDonald, G. Mountzios, P.M. Putora, B. Muoio, U. Ricardi, C.B. Westphalen, A. Wrona, P. Boot, G. Pentheroudakis, C. Belka, F. Lordick, C.A.M. Marijnen, D. Martins-Branco, D. De Ruysscher, J. Barriuso, B. Devnani, M.C. de Jong, A. Prelaj

**Affiliations:** 1 Department of Radiation Oncology, Netherlands Cancer Institute - Antoni van Leeuwenhoek, Amsterdam, The Netherlands; 2 Department of Radiation Oncology, Leiden University Medical Center, Leiden, The Netherlands; 3 School of Cancer Sciences, University of Glasgow, Glasgow, Scotland; 4 CUH/UCC Cancer Centre, Cork University Hospital, Cork, Ireland; 5 Department of Radiation Oncology, Dr. Ram Manohar Lohia Institute of Medical Sciences, Lucknow, India; 6 Department of Radiation Oncology, Radboud University Medical Center, Nijmegen, The Netherlands; 7 Oncology Institute of Southern Switzerland (IOSI), Ente Ospedaliero Cantonale, Bellinzona, Switzerland; 8 Experimental Clinical Oncology, Department of Oncology, Aarhus University Hospital, Aarhus, Denmark; 9 Memorial Sloan Kettering Hospital, New York, USA; 10 Cancer Research UK Lung Cancer Centre of Excellence, University College London Cancer Institute, London, UK; 11 Princess Alexandra Hospital, Greenslopes Private Hospital, University of Queensland, Brisbane, Queensland, Australia; 12 Radiotherapy Department II, Greater Poland Cancer Center, Poznan, Poland; 13 Electroradiology Department, University of Medical Sciences, Poznan, Poland; 14 Medical Oncology, Hospital General Universitario Gregorio Marañón, Madrid, Spain; 15 Department of Oncology and Radiotherapy, Medical University of Gdańsk, Gdańsk, Poland; 16 Department of Medical Oncology and Hematology, Brain Tumor Center, University Hospital and University of Zurich, Zurich, Switzerland; 17 Royal Marsden Hospital, London, UK; 18 4th Oncology Clinic and Clinical Trials Department, Henry Dunant Hospital Center, Athens, Greece; 19 HOCH, Cantonal Hospital St. Gallen, Department of Radiation Oncology, St. Gallen, Switzerland; 20 Department of Radiation Oncology, Inselspital, Bern University Hospital and University of Bern, Bern, Switzerland; 21 Division of Medical Oncology, Oncology Institute of Southern Switzerland (IOSI), Ente Ospedaliero Cantonale, Bellinzona, Switzerland; 22 Department of Oncology, University of Turin, Turin, Italy; 23 Department of Medicine III, University Hospital, LMU Munich, Munich, Germany; 24 German Cancer Consortium (DKTK), Partner Site Munich, Munich, Germany; 25 Comprehensive Cancer Center (CCC Munich LMU), LMU University Hospital Munich, Munich, Germany; 26 Scientific and Medical Division, ESMO – European Society for Medical Oncology, Lugano, Switzerland; 27 Department of Radiation Oncology, University of Munich LMU, Munich, Germany; 28 Department of Medicine II, University of Leipzig Medical Center, Cancer Center Central Germany (CCCG), Leipzig, Germany; 29 Radiation Oncology Department, Maastro Clinic, Maastricht, The Netherlands; 30 Department of Radiotherapy, Erasmus MC Cancer Institute, University Medical Center Rotterdam, Rotterdam, The Netherlands; 31 Department of Medical Oncology, Hospital Universitario 12 de Octubre, Instituto de Investigación Hospital 12 de Octubre (Imas12), Madrid, Spain; 32 Radiation Oncology Department, All India Institute of Medical Sciences, Jodhpur, India; 33 Oncologia Medica Toracica Department, Fondazione IRCCS - Istituto Nazionale Dei Tumori, Milan, Italy

**Keywords:** radiotherapy, targeted therapy, toxicity, systematic review, consensus statements

## Abstract

**Background:**

Combining radiotherapy (RT) with targeted agents may lead to improved treatment outcomes across various tumor types. However, there is a risk of increased toxicity. Unfortunately, high-quality toxicity data are scarce, contributing to a lack of evidence-based guidelines.

**Design:**

ESMO and ESTRO launched a series of systematic literature reviews and evidence-based, multidisciplinary Delphi consensus recommendations on the safety of combining RT with targeted agents. The current paper addresses the safety of combining EGFR, ALK, and BRAF/MEK inhibitors with RT, regardless of (solid) tumor histology. During the two Delphi consensus rounds with 19 international experts, 57 clinical scenarios were evaluated by systematically covering different drug classes and irradiated areas. Based on the systematic literature reviews, safety statements were developed for all scenarios.

**Results:**

During the systematic literature review process for EGFR, ALK, and BRAF/MEK inhibitors, 2745 records were screened, and 110 reports were included in the final literature reviews and the database. Over the course of the subsequent Delphi consensus rounds, agreement was reached on all 57 scenario-specific safety statements.

**Conclusions:**

For most scenarios, concurrently combining RT with targeted agents may lead to increased toxicity. Therefore, we recommend a drug interruption, a drug dosage reduction, or a major RT adaptation in various scenarios.

## Highlights

• This ESMO-ESTRO consensus project assesses the safety of combining RT with targeted therapies.
• Consensus was reached for all 57 scenarios involving RT combined with EGFR, ALK, or BRAF/MEK inhibitors.
• Combining RT with EGFR, ALK, or BRAF/MEK inhibitors may lead to increased toxicity in many scenarios.
• Caution is recommended for most of these drug–RT combinations.

## Introduction

Systemic therapy, along with surgery and radiotherapy (RT), is a fundamental component of cancer treatment. The introduction of targeted agents and immune checkpoint inhibitors (ICIs) has caused a considerable increase in the number of systemic treatment options, and improved treatment outcomes across nearly all cancer types.^1^

Fifty percent of cancer patients receive RT at a certain stage in their treatment, with curative, radical, or palliative intent.^2^, ^3^, ^4^ Accordingly, patients on targeted agents are regularly referred for RT, mainly due to oligometastases, oligoprogression, or for palliative treatment.^5^, ^6^, ^7^, ^8^ The combination of RT with targeted agents may enhance tumor control but can also cause increased toxicity.^9^ Therefore, it is important to determine the safety of RT for patients on these targeted agents.^8^ There is a lack of high-quality toxicity data regarding the combination of RT with various targeted agents. Safety concerns have emerged for several combinations, due to reports of unexpected toxicity.^10^, ^11^, ^12^, ^13^, ^14^, ^15^, ^16^

Thus, oncologists are faced with a clinical dilemma when combining RT with targeted agents. Excessive toxicity should be avoided; however, interrupting the drug or reducing the drug dosage may cause tumor progression or tumor flare, 17, 18, 19 and reducing the RT dose may lead to impaired tumor or symptom control. We are confronted with a serious lack of data, knowledge, and consensus on this topic, and evidence-based, multidisciplinary guidelines are missing for most combinations.^8^^,^^20^^,^^21^ Depending on the drug–RT combination and treatment approach, this knowledge gap may lead to suboptimal treatment decisions (including both over- and undertreatment) or unanticipated toxicity in these patients.

Hence, the European Society for Medical Oncology (ESMO) and the European SocieTy for Radiotherapy and Oncology (ESTRO) launched a series of drug class-specific and irradiated area-specific systematic literature reviews and evidence-based, multidisciplinary Delphi consensus recommendations on the safety of combining RT with 10 classes of targeted agents and ICIs.^22^ Additionally, a framework paper complements this series by outlining the key (radio)biological processes, pharmacological aspects, and general clinical considerations.^9^ The current publication features the systematic reviews and Delphi consensus recommendations regarding the safety of combining RT with the following targeted agents, irrespective of tumor type: epidermal growth factor receptor (EGFR), anaplastic lymphoma kinase (ALK), and B-rapidly accelerated fibrosarcoma (BRAF)/mitogen-activated protein kinase (MEK) inhibitors.

## Methods

### Project governance

This project was a collaboration with ESMO and ESTRO. Both the ESMO board and the ESTRO guidelines committee granted permission. A coordinating committee of ESMO and ESTRO representatives and experts met monthly and managed the project. Researchers at the Netherlands Cancer Institute carried out the daily operational coordination of the project.

### Systematic literature reviews

Systematic searches of the Medline, Embase, and SCOPUS databases were carried out on 21 December 2020 (ALK and BRAF/MEK), and 26 March 2021 (EGFR). For the search strategy and keywords, please refer to Supplementary Tables S1-S4, available at https://doi.org/10.1016/j.esmoop.2026.106076. Only studies describing treatment-related toxicity on concurrent treatment of RT and targeted agents were included, with a maximum drug–RT time interval of five drug half-lives before RT, or 2 weeks after RT. Full inclusion and exclusion criteria are provided in Supplementary Table S5, available at https://doi.org/10.1016/j.esmoop.2026.106076. Title and abstract screening was conducted using a double-blind approach by EA and PB, with a consulting role by MJ. Subsequently, full-text screening was conducted by the same researchers for ALK and BRAF/MEK inhibitors. For EGFR inhibitors, representatives from ESMO (AKG, AP, BD, JB, LCB, SOC) contributed to the full-text screening and review (Supplementary Figures S1-S3, available at https://doi.org/10.1016/j.esmoop.2026.106076). The uniformity and quality of the review process were ensured via ongoing meetings and discussions. For EGFR inhibitors, an additional selection step was introduced to identify the highest-quality trials, to reduce the high number of included reports (Supplementary Figure S4, available at https://doi.org/10.1016/j.esmoop.2026.106076).

To offer drug class-specific and irradiated area-specific toxicity data, the drug-specific systematic literature reviews were separated into six irradiated area-specific reviews: for irradiation of the skin, brain, head and neck, thorax, abdomen/pelvis, and musculoskeletal tissues. Each section contained a summary, providing an accessible overview of the data. General information on frequently used drugs and the biological pathways involved was given as well (Supplementary Material, pages 2-26, available at https://doi.org/10.1016/j.esmoop.2026.106076).

### Literature database

All included reports were incorporated into a literature database built in Microsoft Excel 2016 (Microsoft Corporation, Redmond, WA), with search functionality to select publications with a specific drug target, irradiated area and/or study type. For all included publications, we registered all relevant data in the database, including the number of patients, drug name, drug dose, RT dose, RT fractionation scheme, RT technique, drug and RT timing, primary tumor type, tumor response, acute toxicity, late toxicity, and comparisons with drug and RT monotherapy toxicity.

### Safety recommendations

**Figure 1:**
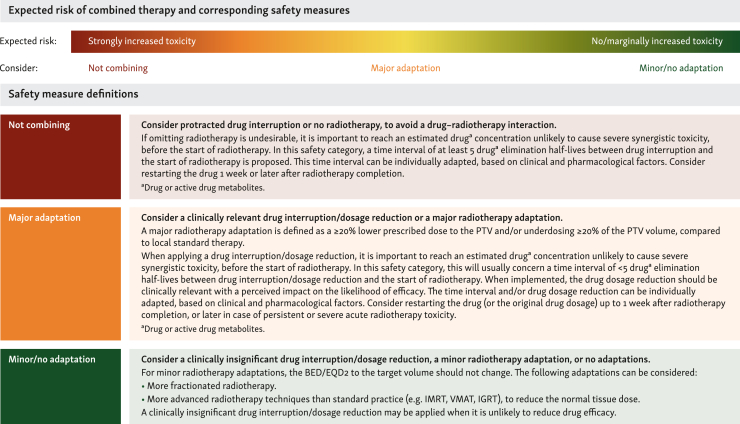
**Predefined safety measure definitions for combining targeted agents with radiotherapy, based on the expected risk.** BED, biologically equivalent dose; EQD_2_, equivalent dose in 2 Gy fractions; IGRT, image-guided radiotherapy; IMRT, intensity-modulated radiotherapy; PTV, planning target volume; VMAT, volumetric-modulated arc therapy.

The daily operational coordinators and the coordinating committee developed three safety measure options as defined in Figure 1: (i) not combining both treatments, (ii) a major treatment adaptation, or (iii) a minor/no treatment adaptation. For each drug class, at least 18 irradiated area-specific and RT scenario-specific (Table 1) safety recommendations were developed. Guided by the systematic literature reviews, EA proposed the most appropriate safety measure option for each safety recommendation.

**Table 1:** Radiotherapy scenario examples

RT scenario	Example
Low-dose palliative RT	*Examples:* 1 × 8, 2 × 8, 5 × 4, 10 × 3 Gy. Often used in patients with metastases and for palliation of symptoms. It generally has a lower risk of RT-induced toxicity. However, low-dose whole brain RT is relatively toxic compared with local high-dose stereotactic RT for brain metastases.
High-dose conventionally fractionated RT	*Examples:* 33 × 2 Gy (5 times per week), 5 × 5 Gy (daily) or similar. Often used in treatments with curative/radical or (neo)adjuvant intent.
High-dose stereotactic RT	*Examples:* ≥14 Gy in 1 fraction, 60 Gy in 5-8 fractions, or similar. Often used in treatments with curative/radical intent. Radical, high-dose stereotactic RT is also increasingly used in the oligometastatic or oligoprogressive setting or to treat brain metastases.

RT, radiotherapy.

The levels of evidence are derived from the ESMO Clinical Practice Guidelines Standard Operating Procedures (adapted from the Infectious Diseases Society of America-United States Public Health Service Grading System) (Supplementary Table S6, available at https://doi.org/10.1016/j.esmoop.2026.106076).^23^^,^^24^

### Modified Delphi process

Ten ESMO experts and 10 ESTRO experts were solicited to participate in a modified Delphi process.^25^ After receiving the systematic literature reviews and the literature database, they were requested to vote whether they agreed or disagreed with the proposed safety measure for each scenario. Upon disagreement, experts were asked to explain their choice and to add supporting (new) literature references, if applicable.

Two Delphi rounds were organized with a digital voting process. During the first Delphi round, ≥90% agreement was required to accept a statement without further voting in the second Delphi round. During the second Delphi round, ≥75% agreement was required to accept a statement.^26^ Statements with ≥90% agreement led to a stronger recommendation than statements with 75%-89% agreement. For statements evaluated in both Delphi rounds, the agreement rates from the second round were leading.

The voting results and expert comments from Delphi round one were evaluated by EA and selected experts (JB, PMP, SOC, UR). If applicable, statements were added, removed, or adapted, before starting Delphi round two. The daily operational coordinators and the ESMO office organized the Delphi process. Microsoft Excel 2016 was used for both developing the questionnaires and analyzing the voting results.

## Results

### EGFR inhibitors

#### Systematic literature review process results

For EGFR inhibitors, 2384 unique records were screened, and 54 reports were included in the literature review and the database. The PRISMA flow diagram^27^ and the full systematic literature review are provided (Supplementary Figure S1, and Supplementary Material, pages 2-13, available at https://doi.org/10.1016/j.esmoop.2026.106076).

#### Drug class and systematic literature review summary

Binding of a ligand to the EGFR leads to structural changes, promoting receptor dimerization and subsequently activation by phosphorylation of the cytoplasmic tail of the receptor.^28^ EGFR activation leads to downstream upregulation of several signaling pathways that are involved in cell survival and proliferation, including the MAPK and phosphatidylinositol 3-kinase (PI3K) pathways.^28^^,^^29^ Upregulated EGFR signaling is a common feature of many tumors, making the inhibition of EGFRs an effective target in several tumor types, including head and neck cancer (also in combination with RT^30^), colorectal cancer and lung cancer.^29^ EGFR inhibition influences DNA repair and cell cycle arrest and can increase radiosensitivity to some extent.^29^^,^^31^, ^32^, ^33^, ^34^, ^35^ Blood–brain barrier penetration of many EGFR inhibitors is generally poor, but significantly better for osimertinib and afatinib.^36^, ^37^, ^38^, ^39^

Data regarding the combination of RT and EGFR inhibitors clearly demonstrate an increased risk of dermatitis, skin rash, and mucositis. In addition, EGFR inhibitors possibly increase the risk of pneumonitis, and they may modestly increase (lower) gastrointestinal (GI)-related and brain-related toxicity, when combined with RT. However, results from most studies suggest that these combinations are feasible.

The data identified for each irradiated area (Supplementary Material, pages 2-13, available at https://doi.org/10.1016/j.esmoop.2026.106076) are summarized here:

• *Skin*^40^, ^41^, ^42^, ^43^, ^44^, ^45^, ^46^, ^47^, ^48^: a large number of high-quality data show a markedly increased risk of high-grade dermatitis and skin rash when RT is combined with EGFR inhibitors. Most data are derived from head and neck cancer trials.
• *Brain*^46^, ^47^, ^48^, ^49^, ^50^, ^51^, ^52^, ^53^, ^54^, ^55^, ^56^, ^57^, ^58^, ^59^, ^60^, ^61^, ^62^: most studies describe the addition of EGFR tyrosine kinase inhibitors (TKIs) (and not monoclonal antibodies) to brain RT. Several studies suggest an increased risk of toxicity when EGFR inhibitors are combined with RT, but most RT-related toxicities are moderately or insignificantly increased.
• *Head and neck*^40^^,^^42^, ^43^, ^44^^,^^63^, ^64^, ^65^, ^66^, ^67^, ^68^, ^69^: a reasonable number of randomized studies and meta-analyses show that combining EGFR antibodies (particularly cetuximab) with head and neck RT significantly increases the risk of grade ≥3 mucositis. Fewer data are available about EGFR TKI combinations, but some show increased grade ≥3 mucositis risks as well.
• *Thorax*^41^^,^^59^^,^^61^^,^^62^^,^^70^, ^71^, ^72^, ^73^, ^74^, ^75^, ^76^, ^77^, ^78^, ^79^, ^80^, ^81^, ^82^, ^83^, ^84^, ^85^, ^86^: EGFR inhibitors may modestly increase the risk of RT toxicities, primarily radiation pneumonitis, when combined with thoracic RT. One retrospective study shows a high radiation pneumonitis risk when RT is combined with osimertinib. The effect of EGFR inhibitors on RT-related esophageal toxicity appears limited.
• *Abdomen/pelvis*^45^^,^^87^, ^88^, ^89^, ^90^, ^91^, ^92^, ^93^, ^94^, ^95^, ^96^: the reported evidence on toxicity of anti-EGFR drugs in combination with RT was mainly derived from studies involving pancreatic or rectal cancer. Toxicity is slightly increased, but considered acceptable, when these drugs are combined with RT or chemoradiotherapy (CRT). Most studies lack a (C)RT-only control arm, but the available data indicate a modestly increased toxicity risk, particularly GI-related.
• Musculoskeletal tissues: no publications were identified that are specific for this RT area, but in the other studies, musculoskeletal toxicity was not a main concern.

#### Delphi process results

The Delphi process was conducted between 18 September 2023 and 14 February 2024. Among the Delphi experts, 95% (19/20) completed both Delphi rounds. There were no other missing answers. The full results from Delphi round one, Delphi round two, and the Delphi statement flow diagram are provided (Supplementary Tables S7, S8 and Supplementary Figure S5, respectively, available at https://doi.org/10.1016/j.esmoop.2026.106076). The final Delphi outcomes for EGFR inhibitors are presented in Table 2.

**Table 2:** EGFR inhibitor consensus statements

For the combination of EGFR inhibitors with radiotherapy to the:				
Irradiated area	Radiotherapy scenario	Recommendation	Agreement rate^a^	Level of evidence
**Skin**	Low-dose palliative	Minor/no adaptation	89%	I^b^
	High-dose conventionally fractionated	Major adaptation	95%	I
	High-dose stereotactic	Major adaptation	100%	II
**Brain**	Low-dose palliative	Minor/no adaptation	89%	II
	High-dose conventionally fractionated	Major adaptation	95%	III
	High-dose stereotactic	Major adaptation	100%	II
**Head & neck**	Low-dose palliative	Major adaptation	100%	I^b^
	High-dose conventionally fractionated	Major adaptation^c^	100%	I
	High-dose stereotactic	Major adaptation	95%	V
**Thorax**	Low-dose palliative	Major adaptation	89%	I^b^
	High-dose conventionally fractionated	Major adaptation	95%	I
	High-dose stereotactic	Major adaptation	95%	III
**Abdomen/pelvis**	Low-dose palliative	Minor/no adaptation	95%	II^b^
	High-dose conventionally fractionated	Major adaptation	100%	II
	High-dose stereotactic	Major adaptation	100%	V
**Musculoskeletal tissues**	Low-dose palliative	Minor/no adaptation	95%	I^b^
	High-dose conventionally fractionated	Major adaptation	100%	I
	High-dose stereotactic	Major adaptation	100%	V
**EXCEPTIONS: for the combination ofosimertinib with radiotherapy to the:**				

**Thorax**	High-dose conventionally fractionated	Not combining	95%	V
	High-dose stereotactic	Not combining	95%	V

EGFR, epidermal growth factor receptor.

a Agreement rates ≥90%: strongly recommended.

b Level of evidence based on data from high radiotherapy dose scenarios.

c This does not apply to intentional concurrent radiotherapy with cetuximab.

### Delphi consensus recommendations

For most scenarios with EGFR inhibitors, we recommend considering a major adaptation (Table 2). For low-dose palliative RT to the skin, brain, abdomen/pelvis, and musculoskeletal tissues, we recommend considering a minor or no adaptation. Due to data from one retrospective study indicating a high risk of radiation pneumonitis, it is recommended to consider not combining osimertinib and high-dose RT to the thorax.

### ALK inhibitors

#### Systematic literature review process results

For ALK inhibitors, 54 unique records were screened, and 15 reports were included in the literature review and the database. For the PRISMA flow diagram^27^ and the full systematic literature review please refer to Supplementary Figure S2, and Supplementary Material, pages 14-18, respectively, available at https://doi.org/10.1016/j.esmoop.2026.106076.

#### Drug class and systematic literature review summary

ALK signaling influences several major oncogenic pathways that contribute to cell survival and proliferation, including RAS, phospholipase C-γ, signal transducer and activator of transcription 3 (STAT3) and PI3K.^97^^,^^98^ Increased ALK activation is primarily caused by chromosomal translocations or inversions, leading to fusion proteins (e.g. EML4-ALK in non-small-cell lung cancer) that lead to ligand-independent activation of the ALK protein kinase domains.^98^^,^^99^ In addition, mutations or ALK amplification can play a role in certain cancer types.^98^^,^^99^

Inhibition of ALK (e.g. alectinib, brigatinib, ceritinib, crizotinib, lorlatinib), IGF1R (brigatinib), MET (crizotinib), RET (alectinib), ROS1 (brigatinib, crizotinib, and lorlatinib) receptor tyrosine kinases downregulates RAS PI3K and STAT3 signaling,^100^ leading to inhibition of growth/proliferation, fewer cells in the radioresistant S-phase,^101^ and inhibition of anti-apoptotic signals, possibly leading to radiosensitization of (mostly rapidly proliferating) normal tissue and tumor cells. So in general, reduced cell survival and repopulation by ALK inhibitors may lead to an increased risk of RT toxicity, whereas inhibition of growth/proliferation could lead to a decreased number of cells in the radiosensitive M-phase,^101^ leading to more radioresistance.

Information on the combination of ALK inhibitors and RT is scarce and consists of case reports and small retrospective series. Most data available are about RT to the central nervous system (CNS). Based on available biological/pharmacological data, it can be hypothesized that survival and repopulation of (particularly fast dividing) normal tissues could be reduced by ALK inhibitors after RT, thus increasing the toxicity of RT. Most interactions could be expected in days-to-weeks after RT (acute phase). However, evidence is scarce and what is available, primarily applies to crizotinib. A case of severe (grade 4) esophagitis has been reported with crizotinib and 10 × 3 Gy on the cervical spine. Gamma Knife RT for brain metastases seemed safe during crizotinib therapy. However, it should be noted that crizotinib is the only ALK inhibitor reported to not cross the blood–brain barrier, whereas CNS-penetrant ALK inhibitors may have a greater potential for side effects when combined with CNS-directed RT.

The data identified for each irradiated area (Supplementary Material, pages 14-18, available at https://doi.org/10.1016/j.esmoop.2026.106076) are summarized here:

• *Skin*^102^: no severe skin toxicity is reported, but the available safety data are very limited.
• *Brain*^103^, ^104^, ^105^, ^106^, ^107^, ^108^, ^109^, ^110^, ^111^, ^112^, ^113^, ^114^, ^115^, ^116^, ^117^, ^118^: in general, no high rates of grade ≥3 toxicity are reported. Most data concern crizotinib use during RT, which has a limited blood–brain barrier penetration.^103^^,^^104^ These results should therefore not be extrapolated to newer TKIs with a higher brain penetration rate. Furthermore, some relatively larger studies combine data from patients using ALK and EGFR inhibitors,^105^, ^106^, ^107^, ^108^, ^109^ which complicates interpretation of the ALK-specific results. A possibly increased risk of ototoxicity (with whole brain RT) should be considered as well.
• *Head and neck*^102^^,^^108^^,^^119^: with only case reports, the available safety data are limited. One case report shows grade 4 ulceration of the hypopharynx with the combination of crizotinib and low-dose palliative RT. Ototoxicity was already described in the brain section.
• *Thorax*^105^^,^^109^^,^^119^^,^^120^: with only retrospective studies and a case report, often with different drug types and timing schedules, the available safety data are limited. The case report showing grade 4 ulceration of the hypopharynx also describes ulceration of the upper esophagus.
• *Abdomen/pelvis*^109^^,^^120^: the available safety data are limited. Two studies interrupting ALK (or EGFR) inhibitors during RT, include abdominal RT and do not describe increased toxicity.^109^^,^^120^
• *Musculoskeletal tissues*^105^^,^^106^^,^^109^: the available safety data are limited, but do not indicate increased toxicity risks.

#### Delphi consensus recommendations

The Delphi process was conducted between 18 September 2023 and 14 February 2024. Among the Delphi experts, 95% (19/20) completed both Delphi rounds. During round one, one expert deliberately refrained from making a decision for two scenarios. In round two, one expert answer was missing for one statement. There were no other missing answers. The full results from Delphi round one, Delphi round two, and the Delphi statement flow diagram are provided (Supplementary Tables S9, S10 and Supplementary Figure S6, respectively, available at https://doi.org/10.1016/j.esmoop.2026.106076). The final Delphi outcomes for ALK inhibitors are presented in Table 3.

**Table 3:** ALK inhibitor consensus statements

For the combination of ALK inhibitors with radiotherapy to the:				
Irradiated area	Radiotherapy scenario	Recommendation	Agreement rate^a^	Level of evidence
**Skin**	Low-dose palliative	Minor/no adaptation	95%	V
	High-dose conventionally fractionated	Major adaptation	95%	V
	High-dose stereotactic	Major adaptation	95%	V
**Brain**	Low-dose palliative	Major adaptation	100%	V
	High-dose conventionally fractionated	Major adaptation	100%	V
	High-dose stereotactic	Major adaptation	95%	IV
**Head & neck**	Low-dose palliative	Major adaptation	84%	V
	High-dose conventionally fractionated	Major adaptation	95%	V
	High-dose stereotactic	Major adaptation	95%	V
**Thorax**	Low-dose palliative	Major adaptation	89%	V
	High-dose conventionally fractionated	Major adaptation	95%	V
	High-dose stereotactic	Major adaptation	94%	V
**Abdomen/pelvis**	Low-dose palliative	Major adaptation	84%	V
	High-dose conventionally fractionated	Major adaptation	95%	V
	High-dose stereotactic	Major adaptation	95%	V
**Musculoskeletal tissues**	Low-dose palliative	Minor/no adaptation	95%	V
	High-dose conventionally fractionated	Major adaptation	100%	V
	High-dose stereotactic	Major adaptation	100%	V

ALK, anaplastic lymphoma kinase.

a Agreement rates ≥90%: strongly recommended.

For most scenarios with ALK inhibitors, we recommend considering a major adaptation (Table 3). Only for low-dose palliative skin and musculoskeletal RT, do we recommend considering a minor/no adaptation. It should be noted that the level of evidence for these scenarios is low.

### BRAF/MEK inhibitors

#### Systematic literature review process results

For BRAF/MEK inhibitors, 307 unique records were screened, and 41 reports were included in the literature review and the database. The PRISMA flow diagram^27^ and the full systematic literature review (Supplementary Figure S3, and Supplementary Material, pages 19-26, respectively) are available at https://doi.org/10.1016/j.esmoop.2026.106076).

#### Drug class and systematic literature review summary

BRAF is part of the RAS-RAF-MEK-ERK signaling pathway. In BRAF-mutated tumors (most frequent mutation: BRAFV600E), activation of BRAF can cause cell proliferation, independently from upstream signaling.^121^ Inhibition of BRAF protein kinases downregulates RAF signaling, leading to inhibition of growth/proliferation in BRAF-mutated cells.^121^ Inhibition of BRAF can also lead to paradoxical MAPK signaling in RAS-mutant and RAS/RAF wild-type cells, leading to fast division of these cells.^122^^,^^123^ Additionally, increased vascular endothelial growth factor production has been described when BRAF is inhibited.^124^ Faster proliferation of keratinocytes can lead to more cells in M-phase and consequently increased skin radiosensitivity. Alternatively, inhibition of proliferation could lead to less repopulation after RT.

MEK inhibitors (MEKi) inhibit activation of MEK, downstream of RAF. The combination with BRAF inhibitors (BRAFi) can increase progression-free and overall survival in patients with BRAF V600-mutated melanoma. Additionally, this combination probably reduces the risk of paradoxical MAPK signaling-induced hyperkeratosis and cutaneous squamous-cell carcinoma.^125^^,^^126^

The data identified for each irradiated area (Supplementary Material, pages 19-26, available at https://doi.org/10.1016/j.esmoop.2026.106076) are summarized here:

• *Skin*^12^^,^^13^^,^^16^^,^^127^, ^128^, ^129^, ^130^, ^131^, ^132^, ^133^, ^134^, ^135^, ^136^, ^137^, ^138^, ^139^, ^140^, ^141^, ^142^, ^143^, ^144^, ^145^, ^146^, ^147^, ^148^, ^149^, ^150^, ^151^, ^152^, ^153^, ^154^, ^155^, ^156^: the literature data clearly indicate an increased risk of skin toxicity when RT is given concurrently or in close proximity to BRAFi ± MEKi, particularly in combination with vemurafenib. Stereotactic RT may be combined without increased skin toxicity when the dose to the skin is low. Reducing the skin dose and temporary drug interruption probably reduces the risk of an interaction but does not exclude this possibility. MEKi-specific data are scarce, but the addition of MEKi to BRAFi does not clearly increase skin toxicity, compared with BRAFi alone.
• *Brain*^12^^,^^16^^,^^135^^,^^136^^,^^138^^,^^145^, ^146^, ^147^, ^148^, ^149^, ^150^, ^151^^,^^157^, ^158^, ^159^, ^160^, ^161^, ^162^, ^163^, ^164^, ^165^, ^166^, ^167^: a number of retrospective studies and case reports have been published with varying methodologies, toxicity analyses, and time intervals between BRAFi ± MEKi and RT. In many studies, BRAFi/MEKi are temporarily paused. Although some studies show higher neurological toxicity rates when BRAFi/MEKi are combined with RT (concurrently or within a certain time interval), several other studies do not report increased toxicity. Combined therapy should therefore not be considered an absolute contra-indication regarding neurological toxicity, but due to the low quality and heterogeneity of the data, increased neurological toxicity cannot be ruled out. We found no studies investigating brain RT combined with a MEKi without a BRAFi.
• *Head and neck*: apart from two case reports concerning increased skin toxicity,^131^^,^^142^ we identified no studies concerning head and neck RT combined with BRAFi/MEKi. Hecht et al. (2015 and 2018) report hearing disorder in 0%-7%, which might also be related to whole brain RT.^12^^,^^146^
• *Thorax*^12^^,^^127^^,^^129^^,^^130^^,^^134^^,^^136^^,^^140^^,^^142^^,^^146^^,^^168^: very limited data are available regarding thoracic RT combined with BRAFi/MEKi. Two case reports show increased toxicity, but the other studies do not clearly indicate increased non-skin-related thoracic RT toxicity. Nevertheless, caution is needed when combining these therapies due to the low number of toxicity data.
• *Abdomen/pelvis*^128^^,^^130^^,^^132^^,^^135^^,^^136^^,^^140^^,^^142^^,^^152^, ^153^, ^154^: although some increased toxicity is reported, very limited data are available regarding abdominal/pelvic RT combined with BRAFi/MEKi. Caution is needed when combining these therapies.
• *Musculoskeletal tissues*^12^^,^^132^^,^^139^^,^^140^^,^^142^, ^143^, ^144^, ^145^, ^146^: no increased musculoskeletal-specific toxicity is reported.

#### Delphi consensus recommendations

The Delphi process was conducted between 18 September 2023 and 14 February 2024. Among the Delphi experts, 95% (19/20) completed both Delphi rounds. There were no other missing answers. The full results from Delphi round one, Delphi round two, and the Delphi statement flow diagram (Supplementary Tables S11, S12, and Supplementary Figure S7, respectively, available at https://doi.org/10.1016/j.esmoop.2026.106076), are provided. The final Delphi outcomes for BRAF/MEK inhibitors are presented in Table 4.

**Table 4:** BRAF/MEK inhibitor consensus statements

For the combination of BRAF/MEK inhibitors with radiotherapy to the:				
Irradiated area	Radiotherapy scenario	Recommendation	Agreement rate^a^	Level of evidence
**Skin**	Low-dose palliative	Major adaptation	100%	IV
	High-dose conventionally fractionated	Not combining	100%	IV
	High-dose stereotactic	Not combining	95%	IV
**Brain**	Low-dose palliative	Major adaptation	100%	IV
	High-dose conventionally fractionated	Major adaptation	100%	IV
	High-dose stereotactic	Major adaptation	100%	IV
**Head & neck**	Low-dose palliative	Major adaptation	100%	V
	High-dose conventionally fractionated	Major adaptation	100%	V
	High-dose stereotactic	Major adaptation	100%	V
**Thorax**	Low-dose palliative	Major adaptation	100%	V
	High-dose conventionally fractionated	Major adaptation	100%	V
	High-dose stereotactic	Major adaptation	100%	V
**Abdomen/pelvis**	Low-dose palliative	Major adaptation	100%	V
	High-dose conventionally fractionated	Major adaptation	100%	III
	High-dose stereotactic	Major adaptation	100%	V
**Musculoskeletal tissues**	Low-dose palliative	Major adaptation	95%	IV
	High-dose conventionally fractionated	Major adaptation	100%	V
	High-dose stereotactic	Major adaptation	100%	V
**EXCEPTION: for the combination of vemurafenib with radiotherapy to the:**				

**Skin**	Low-dose palliative	Not combining	100%	IV

BRAF, B-rapidly accelerated fibrosarcoma; MEK, mitogen-activated protein kinase.

a Agreement rates ≥90%: strongly recommended.

Due to the lack of high-quality toxicity data and the possible increased toxicity, we recommend considering a major adaptation for most RT combinations with BRAF/MEK inhibitors. As the data clearly indicate increased skin toxicity, it is recommended to consider not combining BRAF/MEK inhibitors and RT in cases of high-dose skin RT. For vemurafenib, it is recommended to consider not combining this with skin RT, not even with low-dose palliative RT.

## Discussion

These ESMO-ESTRO consensus recommendations provide evidence-based guidance on the safety of combining RT with targeted cancer therapies. In the current publication, we provide the systematic literature reviews and recommendations on the safety of combining RT with EGFR, ALK, or BRAF/MEK inhibitors. For most combination scenarios with these drugs and RT, we recommend exercising caution.

The development of these scenario-specific and multidisciplinary ESMO-ESTRO consensus recommendations required an intensive interdisciplinary collaboration and the development of drug class-specific and RT scenario-specific systematic reviews. With these statements, we aim to provide pragmatic and evidence-based safety recommendations for clinical practice, based on the current best available evidence and supported by expert validation. These recommendations are not intended to serve as strict guidelines, nor to substitute high-quality registries or clinical trials that combine these drugs with RT. During decision making, various patient and treatment characteristics (including previous RT) should be evaluated, as described in more detail in the complementary ESMO-ESTRO framework paper.^9^ Furthermore, the expected toxicity should always be considered in light of the anticipated or ongoing treatment efficacy.

This comprehensive project comprising multiple papers has some limitations. The extensive size of the systematic literature reviews and the Delphi processes in this project resulted in relevant time intervals between the original literature searches, the Delphi consensus processes, and publication of the manuscripts. To mitigate the effects as far as possible, we invited the experts to provide (new) literature references if they did not agree with a proposed safety recommendation. The high agreement rates and the limited amount of suggested new literature indicate the relevance and the validity of the consensus recommendations. However, the published recommendations should always be evaluated in consideration of any new data that may enhance our knowledge of combined treatment toxicity.

The lack of high-quality evidence for many drug–RT combination scenarios remains a concerning reality for clinicians, particularly in the context of ALK and BRAF/MEK inhibitors. This is reflected in the evidence levels assigned to each recommendation. Major limitations of many reviewed studies are their retrospective design and low patient numbers. Other important limitations include the absence of RT-only or drug-only control groups, limited RT dose and fractionation details, varying intervals between drug administration and RT, pooled results of different targeted agents, concurrent use of chemotherapy, heterogeneous toxicity reporting, and limited follow-up. We carefully addressed these limitations in the literature database and full systematic literature reviews (Supplementary Material, pages 2-26, available at https://doi.org/10.1016/j.esmoop.2026.106076).

Introducing new targeted agents without first acquiring toxicity data for their combination with RT causes challenging clinical dilemmas, emphasizing the critical need for strategies to be developed to collect these data. To accomplish this, it is essential to increase awareness within the pharmaceutical industry and academic collaborative groups. Cross-disciplinary collaborations, aimed at collecting these data by developing preclinical studies, clinical trials, and prospective data registries combining targeted agents with RT should be initiated.^8^^,^^169^^,^^170^ However, the results from this comprehensive project provide valuable guidance for clinicians facing the dilemma of combining RT with targeted agents.

## References

[1] Scott E.C., Baines A.C., Gong Y. (2023). Trends in the approval of cancer therapies by the FDA in the twenty-first century. Nat Rev Drug Discov.

[2] Yap M.L., Zubizarreta E., Bray F., Ferlay J., Barton M. (2016). Global access to radiotherapy services: have we made progress during the past decade?. J Glob Oncol.

[3] Citrin D.E. (2017). Recent developments in radiotherapy. N Engl J Med.

[4] De Ruysscher D., Niedermann G., Burnet N.G., Siva S., Lee A.W.M., Hegi-Johnson F. (2019). Radiotherapy toxicity. Nat Rev Dis Primers.

[5] Cheung P. (2016). Stereotactic body radiotherapy for oligoprogressive cancer. Br J Radiol.

[6] Lievens Y., Guckenberger M., Gomez D. (2020). Defining oligometastatic disease from a radiation oncology perspective: an ESTRO-ASTRO consensus document. Radiother Oncol.

[7] Lutz S.T., Chow E.L., Hartsell W.F., Konski A.A. (2007). A review of hypofractionated palliative radiotherapy. Cancer.

[8] van Aken E.S.M., van der Linden Y.M., van Thienen J.V. (2022). Hypofractionated radiotherapy combined with targeted therapy or immunotherapy: Dutch survey on current practice, knowledge and challenges. Clin Transl Radiat Oncol.

[9] van Aken E.S.M., Devnani B., Castelo-Branco L. (2025). ESMO-ESTRO framework for assessing the interactions and safety of combining radiotherapy with targeted cancer therapies or immunotherapy. Radiother Oncol.

[10] van Aken E.S.M., Beeker A., Houtenbos I. (2022). Unexpected toxicity of CDK4/6 inhibitor palbociclib and radiotherapy. Cancer Rep (Hoboken).

[11] de Haan R., van den Heuvel M.M., van Diessen J. (2021). Phase I and pharmacologic study of olaparib in combination with high-dose radiotherapy with and without concurrent cisplatin for non-small cell lung cancer. Clin Cancer Res.

[12] Hecht M., Zimmer L., Loquai C. (2015). Radiosensitization by BRAF inhibitor therapy-mechanism and frequency of toxicity in melanoma patients. Ann Oncol.

[13] Harding J.J., Barker C.A., Carvajal R.D., Wolchok J.D., Chapman P.B., Lacouture M.E. (2014). Cutis verticis gyrata in association with vemurafenib and whole-brain radiotherapy. J Clin Oncol.

[14] Barney B.M., Markovic S.N., Laack N.N. (2013). Increased bowel toxicity in patients treated with a vascular endothelial growth factor inhibitor (VEGFI) after stereotactic body radiation therapy (SBRT). Int J Radiat Oncol Biol Phys.

[15] Peters N.A., Richel D.J., Verhoeff J.J., Stalpers L.J. (2008). Bowel perforation after radiotherapy in a patient receiving sorafenib. J Clin Oncol.

[16] Kroeze S.G.C., Fritz C., Schaule J. (2021). Continued versus interrupted targeted therapy during metastasis-directed stereotactic radiotherapy: a retrospective multi-center safety and efficacy analysis. Cancers (Basel).

[17] Chaft J.E., Oxnard G.R., Sima C.S., Kris M.G., Miller V.A., Riely G.J. (2011). Disease flare after tyrosine kinase inhibitor discontinuation in patients with EGFR-mutant lung cancer and acquired resistance to erlotinib or gefitinib: implications for clinical trial design. Clin Cancer Res.

[18] Pop O., Pirvu A., Toffart A.C., Moro-Sibilot D. (2012). Disease flare after treatment discontinuation in a patient with EML4-ALK lung cancer and acquired resistance to crizotinib. J Thorac Oncol.

[19] Amirault M., Choo S., Waxweiler T. (2021). Tumor flare of brain metastases upon dose interruption of sunitinib in a patient with metastatic renal cell carcinoma. Cancer Treat Res Commun.

[20] Amin N.P., Remick J., Agarwal M., Desai N.A., Bergom C., Simone C.B. (2019). Concurrent radiation and immunotherapy: survey of practice patterns in the United States. Am J Clin Oncol.

[21] Kraus K.M., Fischer J.C., Borm K.J. (2021). Evaluation of practical experiences of German speaking radiation oncologists in combining radiation therapy with checkpoint blockade. Sci Rep.

[22] van Aken E.S.M., Devnani B., Prelaj A. (2026). ESMO-ESTRO consensus statements on the safety of combining radiotherapy with immune checkpoint inhibitors, VEGF(R) inhibitors, or multitargeted tyrosine kinase inhibitors. Ann Oncol.

[23] Dykewicz C.A. (2001). Summary of the guidelines for preventing opportunistic infections among hematopoietic stem cell transplant recipients. Clin Infect Dis.

[24] Gross P.A., Barrett T.L., Dellinger E.P. (1994). Purpose of quality standards for infectious diseases. Infectious Diseases Society of America. Clin Infect Dis.

[25] Nasa P., Jain R., Juneja D. (2021). Delphi methodology in healthcare research: how to decide its appropriateness. World J Methodol.

[26] Barrios M., Guilera G., Nuño L., Gómez-Benito J. (2021). Consensus in the delphi method: what makes a decision change?. Technol Forecast Soc Change.

[27] Page M.J., McKenzie J.E., Bossuyt P.M. (2021). The PRISMA 2020 statement: an updated guideline for reporting systematic reviews. BMJ.

[28] Sigismund S., Avanzato D., Lanzetti L. (2018). Emerging functions of the EGFR in cancer. Mol Oncol.

[29] Wheeler D.L., Dunn E.F., Harari P.M. (2010). Understanding resistance to EGFR inhibitors-impact on future treatment strategies. Nat Rev Clin Oncol.

[30] Bonner J.A., Harari P.M., Giralt J. (2006). Radiotherapy plus cetuximab for squamous-cell carcinoma of the head and neck. N Engl J Med.

[31] Brand T.M., Iida M., Luthar N., Starr M.M., Huppert E.J., Wheeler D.L. (2013). Nuclear EGFR as a molecular target in cancer. Radiother Oncol.

[32] Dittmann K., Mayer C., Rodemann H.P. (2005). Inhibition of radiation-induced EGFR nuclear import by C225 (Cetuximab) suppresses DNA-PK activity. Radiother Oncol.

[33] Horn D., Hess J., Freier K., Hoffmann J., Freudlsperger C. (2015). Targeting EGFR-PI3K-AKT-mTOR signaling enhances radiosensitivity in head and neck squamous cell carcinoma. Expert Opin Ther Targets.

[34] Kriegs M., Gurtner K., Can Y. (2015). Radiosensitization of NSCLC cells by EGFR inhibition is the result of an enhanced p53-dependent G1 arrest. Radiother Oncol.

[35] Kriegs M., Kasten-Pisula U., Riepen B. (2016). Radiosensitization of HNSCC cells by EGFR inhibition depends on the induction of cell cycle arrests. Oncotarget.

[36] Colclough N., Chen K., Johnstrom P. (2021). Preclinical comparison of the blood-brain barrier permeability of osimertinib with other EGFR TKIs. Clin Cancer Res.

[37] Varrone A., Varnas K., Jucaite A. (2020). A PET study in healthy subjects of brain exposure of (11)C-labelled osimertinib - a drug intended for treatment of brain metastases in non-small cell lung cancer. J Cereb Blood Flow Metab.

[38] Hochmair M. (2018). Medical treatment options for patients with epidermal growth factor receptor mutation-positive non-small cell lung cancer suffering from brain metastases and/or leptomeningeal disease. Target Oncol.

[39] Shah R., Lester J.F. (2020). Tyrosine kinase inhibitors for the treatment of EGFR mutation-positive non-small-cell lung cancer: a clash of the generations. Clin Lung Cancer.

[40] Wang B.C., Shi L.L., Fu C. (2019). A meta-analysis of cisplatin-based concurrent chemoradiotherapy with or without cetuximab for locoregionally advanced nasopharyngeal carcinoma. Medicine.

[41] Liu R., Wei S., Zhang Q. (2019). Epidermal growth factor receptor tyrosine kinase inhibitors combined with thoracic radiotherapy or chemoradiotherapy for advanced or metastatic non-small cell lung cancer: a systematic review and meta-analysis of single-arm trials. Medicine (Baltimore).

[42] Wang N., Wang K., Song F., Liu Y. (2018). Cetuximab in combination with chemoradiotherapy for nasopharyngeal carcinoma: a meta-analysis. Indian J Cancer.

[43] Liang Z.G., Lin G.X., Ye J.X. (2018). Cetuximab or nimotuzumab versus cisplatin concurrent with radiotherapy for local-regionally advanced nasopharyngeal carcinoma: a meta-analysis. Asian Pac J Cancer Prev.

[44] Tejwani A., Wu S., Jia Y., Agulnik M., Millender L., Lacouture M.E. (2009). Increased risk of high-grade dermatologic toxicities with radiation plus epidermal growth factor receptor inhibitor therapy. Cancer.

[45] Zhong X., Zhou Y., Cui W. (2020). The addition of EGFR inhibitors in neoadjuvant therapy for KRAS-wild type locally advanced rectal cancer patients: a systematic review and meta-analysis. Front Pharmacol.

[46] Zheng M.H., Sun H.T., Xu J.G. (2016). Combining whole-brain radiotherapy with gefitinib/erlotinib for brain metastases from non-small-cell lung cancer: a meta-analysis. Biomed Res Int.

[47] Wang X., Xu Y., Tang W., Liu L. (2018). Efficacy and safety of radiotherapy plus EGFR-TKIs in NSCLC patients with brain metastases: a meta-analysis of published data. Transl Oncol.

[48] Jiang T., Min W., Li Y., Yue Z., Wu C., Zhou C. (2016). Radiotherapy plus EGFR TKIs in non-small cell lung cancer patients with brain metastases: an update meta-analysis. Cancer Med.

[49] Sperduto P.W., Wang M., Robins H.I. (2013). A phase 3 trial of whole brain radiation therapy and stereotactic radiosurgery alone versus WBRT and SRS with temozolomide or erlotinib for non-small cell lung cancer and 1 to 3 brain metastases: Radiation Therapy Oncology Group 0320. Int J Radiat Oncol Biol Phys.

[50] Fleischhack G., Massimino M., Warmuth-Metz M. (2019). Nimotuzumab and radiotherapy for treatment of newly diagnosed diffuse intrinsic pontine glioma (DIPG): a phase III clinical study. J Neurooncol.

[51] Chakravarti A., Wang M., Robins H.I. (2013). RTOG 0211: a phase 1/2 study of radiation therapy with concurrent gefitinib for newly diagnosed glioblastoma patients. Int J Radiat Oncol Biol Phys.

[52] Pesce G.A., Klingbiel D., Ribi K. (2012). Outcome, quality of life and cognitive function of patients with brain metastases from non-small cell lung cancer treated with whole brain radiotherapy combined with gefitinib or temozolomide. A randomised phase II trial of the Swiss Group for Clinical Cancer Research (SAKK 70/03). Eur J Cancer.

[53] Prados M.D., Chang S.M., Butowski N. (2009). Phase II study of erlotinib plus temozolomide during and after radiation therapy in patients with newly diagnosed glioblastoma multiforme or gliosarcoma. J Clin Oncol.

[54] Du X.J., Li X.M., Cai L.B. (2019). Efficacy and safety of nimotuzumab in addition to radiotherapy and temozolomide for cerebral glioblastoma: a phase II multicenter clinical trial. J Cancer.

[55] Macy M.E., Kieran M.W., Chi S.N. (2017). A pediatric trial of radiation/cetuximab followed by irinotecan/cetuximab in newly diagnosed diffuse pontine gliomas and high-grade astrocytomas: a Pediatric Oncology Experimental Therapeutics Investigators’ Consortium study. Pediatr Blood Cancer.

[56] Lee S.M., Lewanski C.R., Counsell N. (2014). Randomized trial of erlotinib plus whole-brain radiotherapy for NSCLC patients with multiple brain metastases. J Natl Cancer Inst.

[57] Zhou L., He J., Xiong W. (2016). Impact of whole brain radiation therapy on CSF penetration ability of Icotinib in EGFR-mutated non-small cell lung cancer patients with brain metastases: results of phase I dose-escalation study. Lung Cancer.

[58] Yomo S., Serizawa T., Yamamoto M. (2019). The impact of EGFR-TKI use on clinical outcomes of lung adenocarcinoma patients with brain metastases after Gamma Knife radiosurgery: a propensity score-matched analysis based on extended JLGK0901 dataset (JLGK0901-EGFR-TKI). J Neurooncol.

[59] Santarpia M., Altavilla G., Borsellino N. (2020). High-dose radiotherapy for oligo-progressive NSCLC receiving EGFR tyrosine kinase inhibitors: real world data. In Vivo.

[60] Chen H., Wu A., Tao H. (2018). Concurrent versus sequential whole brain radiotherapy and TKI in EGFR-mutated NSCLC patients with brain metastasis: a single institution retrospective analysis. Medicine (Baltimore).

[61] Li L., Liu L.Y., Chen M. (2015). A pilot study of conformal radiotherapy combined with erlotinib-based multimodality therapy in newly diagnosed metastatic non-small-cell lung cancer. Eur Rev Med Pharmacol Sci.

[62] Wang Y., Li Y., Xia L. (2018). Continued EGFR-TKI with concurrent radiotherapy to improve time to progression (TTP) in patients with locally progressive non-small cell lung cancer (NSCLC) after front-line EGFR-TKI treatment. Clin Transl Oncol.

[63] Gebre-Medhin M., Brun E., Engstrom P. (2021). ARTSCAN III: a randomized phase III study comparing chemoradiotherapy with cisplatin versus cetuximab in patients with locoregionally advanced head and neck squamous cell cancer. J Clin Oncol.

[64] Ghi M.G., Paccagnella A., Ferrari D. (2017). Induction TPF followed by concomitant treatment versus concomitant treatment alone in locally advanced head and neck cancer. A phase II-III trial. Ann Oncol.

[65] Patil V.M., Noronha V., Joshi A. (2019). A randomized phase 3 trial comparing nimotuzumab plus cisplatin chemoradiotherapy versus cisplatin chemoradiotherapy alone in locally advanced head and neck cancer. Cancer.

[66] Gregoire V., Hamoir M., Chen C. (2011). Gefitinib plus cisplatin and radiotherapy in previously untreated head and neck squamous cell carcinoma: a phase II, randomized, double-blind, placebo-controlled study. Radiother Oncol.

[67] Saini S.K., Srivastava S., Dixit A.K. (2018). Gefitinib with concurrent chemoradiation in locally advanced head neck cancer. Gac Mex Oncol.

[68] Yao M., Woods C., Lavertu P. (2016). Phase II study of erlotinib and docetaxel with concurrent intensity-modulated radiotherapy in locally advanced head and neck squamous cell carcinoma. Head Neck.

[69] Margalit D.N., Haddad R.I., Tishler R.B. (2019). A phase 1 study of afatinib in combination with postoperative radiation therapy with and without weekly docetaxel in intermediate- and high-risk patients with resected squamous cell carcinoma of the head and neck. Int J Radiat Oncol Biol Phys.

[70] Bradley J.D., Hu C., Komaki R.R. (2020). Long-term results of NRG oncology RTOG 0617: standard- versus high-dose chemoradiotherapy with or without cetuximab for unresectable stage III non-small-cell lung cancer. J Clin Oncol.

[71] Chun S.G., Hu C., Choy H. (2017). Impact of intensity-modulated radiation therapy technique for locally advanced non-small-cell lung cancer: a secondary analysis of the NRG Oncology RTOG 0617 randomized clinical trial. J Clin Oncol.

[72] Bradley J.D., Paulus R., Komaki R. (2015). Standard-dose versus high-dose conformal radiotherapy with concurrent and consolidation carboplatin plus paclitaxel with or without cetuximab for patients with stage IIIA or IIIB non-small-cell lung cancer (RTOG 0617): a randomised, two-by-two factorial phase 3 study. Lancet Oncol.

[73] Crosby T., Hurt C.N., Falk S. (2013). Chemoradiotherapy with or without cetuximab in patients with oesophageal cancer (SCOPE1): a multicentre, phase 2/3 randomised trial. Lancet Oncol.

[74] Crosby T., Hurt C.N., Falk S. (2017). Long-term results and recurrence patterns from SCOPE-1: a phase II/III randomised trial of definitive chemoradiotherapy +/- cetuximab in oesophageal cancer. Br J Cancer.

[75] Ruhstaller T., Thuss-Patience P., Hayoz S. (2018). Neoadjuvant chemotherapy followed by chemoradiation and surgery with and without cetuximab in patients with resectable esophageal cancer: a randomized, open-label, phase III trial (SAKK 75/08). Ann Oncol.

[76] Xing L., Wu G., Wang L. (2020). Erlotinib vs etoposide/cisplatin with radiotherapy in unresectable stage III epidermal growth factor receptor mutation-positive non-small-cell lung cancer: a multicenter, randomized, open-label, phase 2 trial. Int J Radiat Oncol Biol Phys.

[77] Liu T., He Z., Dang J., Li G. (2019). Comparative efficacy and safety for different chemotherapy regimens used concurrently with thoracic radiation for locally advanced non-small cell lung cancer: a systematic review and network meta-analysis. Radiat Oncol.

[78] Choi H.J., Sohn J.H., Lee C.G. (2011). A phase I study of nimotuzumab in combination with radiotherapy in stages IIB-IV non-small cell lung cancer unsuitable for radical therapy: Korean results. Lung Cancer.

[79] Xie C., Jing Z., Luo H. (2020). Chemoradiotherapy with extended nodal irradiation and/or erlotinib in locally advanced oesophageal squamous cell cancer: long-term update of a randomised phase 3 trial. Br J Cancer.

[80] Wu S.X., Wang L.H., Luo H.L. (2018). Randomised phase III trial of concurrent chemoradiotherapy with extended nodal irradiation and erlotinib in patients with inoperable oesophageal squamous cell cancer. Eur J Cancer.

[81] Swaminath A., Wright J.R., Tsakiridis T.K. (2016). A phase II trial of erlotinib and concurrent palliative thoracic radiation for patients with non-small-cell lung cancer. Clin Lung Cancer.

[82] Komaki R., Allen P.K., Wei X. (2015). Adding erlotinib to chemoradiation improves overall survival but not progression-free survival in stage III non-small cell lung cancer. Int J Radiat Oncol Biol Phys.

[83] Chan O.S.H., Lam K.C., Li J.Y.C. (2020). ATOM: a phase II study to assess efficacy of preemptive local ablative therapy to residual oligometastases of NSCLC after EGFR TKI. Lung Cancer.

[84] Marquez-Medina D., Chachoua A., Martin-Marco A. (2013). Continued erlotinib maintenance and salvage radiation for solitary areas of disease progression: a useful strategy in selected non-small cell lung cancers?. Clin Transl Oncol.

[85] Syahruddin E., Huswatun A.L., Prabowo A. (2018). Efficacy of gefitinib and radiotherapy combination in Indonesian patients with lung adenocarcinoma. Rom J Intern Med.

[86] Jia W., Guo H., Jing W. (2020). An especially high rate of radiation pneumonitis observed in patients treated with thoracic radiotherapy and simultaneous osimertinib. Radiother Oncol.

[87] Hammel P., Huguet F., van Laethem J.L. (2016). Effect of chemoradiotherapy vs chemotherapy on survival in patients with locally advanced pancreatic cancer controlled after 4 months of gemcitabine with or without erlotinib: the LAP07 randomized clinical trial. JAMA.

[88] Hammel P. (Updated December 11, 2015). Gemcitabine with or without capecitabine and/or radiation therapy or gemcitabine with or without erlotinib in treating patients with locally advanced pancreatic cancer that cannot be removed by surgery. https://clinicaltrials.gov/ct2/show/NCT00634725.

[89] Berlin J.D., Feng Y., Catalano P. (2018). An intergroup randomized phase II study of bevacizumab or cetuximab in combination with gemcitabine and in combination with chemoradiation in patients with resected pancreatic carcinoma: a trial of the ECOG-ACRIN Cancer Research Group (E2204). Oncology.

[90] Maurel J., Sanchez-Cabus S., Laquente B. (2018). Outcomes after neoadjuvant treatment with gemcitabine and erlotinib followed by gemcitabine-erlotinib and radiotherapy for resectable pancreatic cancer (GEMCAD 10-03 trial). Cancer Chemother Pharmacol.

[91] Robertson J.M., Margolis J., Jury R.P. (2012). Phase I study of conformal radiotherapy and concurrent full-dose gemcitabine with erlotinib for unresected pancreatic cancer. Int J Radiat Oncol Biol Phys.

[92] Bertolini F., Chiara S., Bengala C. (2009). Neoadjuvant treatment with single-agent cetuximab followed by 5-FU, cetuximab, and pelvic radiotherapy: a phase II study in locally advanced rectal cancer. Int J Radiat Oncol Biol Phys.

[93] Valentini V., De Paoli A., Gambacorta M.A. (2008). Infusional 5-fluorouracil and ZD1839 (Gefitinib-Iressa) in combination with preoperative radiotherapy in patients with locally advanced rectal cancer: a phase I and II trial (1839IL/0092). Int J Radiat Oncol Biol Phys.

[94] Gambacorta M.A., De Paoli A., Lupattelli M. (2018). Phase I and II trial on infusional 5-fluorouracil and gefitinib in combination with preoperative radiotherapy in rectal cancer: 10-years median follow-up. Clin Transl Radiat Oncol.

[95] Leichman C.G., McDonough S.L., Smalley S.R. (2018). Cetuximab combined with induction oxaliplatin and capecitabine, followed by neoadjuvant chemoradiation for locally advanced rectal cancer: SWOG 0713. Clin Colorectal Cancer.

[96] Dewdney A., Cunningham D., Tabernero J. (2012). Multicenter randomized phase II clinical trial comparing neoadjuvant oxaliplatin, capecitabine, and preoperative radiotherapy with or without cetuximab followed by total mesorectal excision in patients with high-risk rectal cancer (EXPERT-C). J Clin Oncol.

[97] Chiarle R., Voena C., Ambrogio C., Piva R., Inghirami G. (2008). The anaplastic lymphoma kinase in the pathogenesis of cancer. Nat Rev Cancer.

[98] Roskoski R. (2013). Anaplastic lymphoma kinase (ALK): structure, oncogenic activation, and pharmacological inhibition. Pharmacol Res.

[99] Hallberg B., Palmer R.H. (2016). The role of the ALK receptor in cancer biology. Ann Oncol.

[100] Cooper W.A., Lam D.C., O’Toole S.A., Minna J.D. (2013). Molecular biology of lung cancer. J Thorac Dis.

[101] Pawlik T.M., Keyomarsi K. (2004). Role of cell cycle in mediating sensitivity to radiotherapy. Int J Radiat Oncol Biol Phys.

[102] Brunac A.C., Laprie A., Castex M.P. (2020). The combination of radiotherapy and ALK inhibitors is effective in the treatment of intraosseous rhabdomyosarcoma with FUS-TFCP2 fusion transcript. Pediatr Blood Cancer.

[103] Petrelli F., Lazzari C., Ardito R. (2018). Efficacy of ALK inhibitors on NSCLC brain metastases: a systematic review and pooled analysis of 21 studies. PLoS One.

[104] Costa D.B., Kobayashi S., Pandya S.S. (2011). CSF concentration of the anaplastic lymphoma kinase inhibitor crizotinib. J Clin Oncol.

[105] Borghetti P., Bonu M.L., Giubbolini R. (2019). Concomitant radiotherapy and TKI in metastatic EGFR- or ALK-mutated non-small cell lung cancer: a multicentric analysis on behalf of AIRO lung cancer study group. Radiol Med.

[106] Borghetti P., Bonu M.L., Roca E. (2018). Radiotherapy and tyrosine kinase inhibitors in stage IV non-small cell lung cancer: real-life experience. In Vivo.

[107] Miller J.A., Kotecha R., Ahluwalia M.S. (2017). The impact of tumor biology on survival and response to radiation therapy among patients with non-small cell lung cancer brain metastases. Pract Radiat Oncol.

[108] Nakashima T., Nonoshita T., Hirata H. (2020). Adverse events of concurrent radiotherapy and ALK inhibitors for brain metastases of ALK-rearranged lung adenocarcinoma. In Vivo.

[109] Weickhardt A.J., Scheier B., Burke J.M. (2012). Local ablative therapy of oligoprogressive disease prolongs disease control by tyrosine kinase inhibitors in oncogene-addicted non-small-cell lung cancer. J Thorac Oncol.

[110] Choi J.W., Kong D.S., Seol H.J. (2016). Outcomes of gamma knife radiosurgery in combination with crizotinib for patients with brain metastasis from non-small cell lung cancer. World Neurosurg.

[111] Dudnik E., Siegal T., Zach L. (2016). Durable brain response with pulse-dose crizotinib and ceritinib in ALK-positive non-small cell lung cancer compared with brain radiotherapy. J Clin Neurosci.

[112] Onesti C.E., Iacono D., Angelini S. (2019). Four lines of anaplastic lymphoma kinase inhibitors and brain radiotherapy in a long-surviving non-small-cell lung cancer anaplastic lymphoma kinase-positive patient with leptomeningeal carcinomatosis. Anticancer Drugs.

[113] Pinto I.G., Lee M., Graziano S., Lacombe M., Gajra A. (2014). Concurrent crizotinib and whole-brain radiation for brain metastases in ALK-positive lung adenocarcinoma. Lung Cancer Manag.

[114] Tanigawa K., Mizuno K., Kamenohara Y., Unoki T., Misono S., Inoue H. (2019). Effect of bevacizumab on brain radiation necrosis in anaplastic lymphoma kinase-positive lung cancer. Respirol Case Rep.

[115] Urbanska E.M., Santoni-Rugiu E., Melchior L.C., Carlsen J.F., Sorensen J.B. (2021). Intracranial response of ALK non-small-cell lung cancer to second-line dose-escalated brigatinib after alectinib discontinuation due to drug-induced hepatitis and relapse after whole brain radiotherapy followed by stereotactic radiosurgery. Clin Lung Cancer.

[116] Ou S.H., Klempner S.J., Azada M.C., Rausei-Mills V., Duma C. (2015). Radiation necrosis presenting as pseudoprogression (PsP) during alectinib treatment of previously radiated brain metastases in ALK-positive NSCLC: implications for disease assessment and management. Lung Cancer.

[117] Ou S.H., Weitz M., Jalas J.R. (2016). Alectinib induced CNS radiation necrosis in an ALK+NSCLC patient with a remote (7 years) history of brain radiation. Lung Cancer.

[118] Zhu V.W., Nagasaka M., Kubota T. (2020). Symptomatic CNS radiation necrosis requiring neurosurgical resection during treatment with lorlatinib in ALK-rearranged NSCLC: a report of two cases. Lung Cancer (Auckl).

[119] Zimmermann M.H., Beckmann G., Jung P., Flentje M. (2017). Hypopharyngeal and upper esophageal ulceration after cervical spine radiotherapy concurrent with crizotinib. Strahlenther Onkol.

[120] Gan G.N., Weickhardt A.J., Scheier B. (2014). Stereotactic radiation therapy can safely and durably control sites of extra-central nervous system oligoprogressive disease in anaplastic lymphoma kinase-positive lung cancer patients receiving crizotinib. Int J Radiat Oncol Biol Phys.

[121] Ascierto P.A., Kirkwood J.M., Grob J.J. (2012). The role of BRAF V600 mutation in melanoma. J Transl Med.

[122] Poulikakos P.I., Zhang C., Bollag G., Shokat K.M., Rosen N. (2010). RAF inhibitors transactivate RAF dimers and ERK signalling in cells with wild-type BRAF. Nature.

[123] Hatzivassiliou G., Song K., Yen I. (2010). RAF inhibitors prime wild-type RAF to activate the MAPK pathway and enhance growth. Nature.

[124] Wang T., Xiao M., Ge Y. (2015). BRAF inhibition stimulates melanoma-associated macrophages to drive tumor growth. Clin Cancer Res.

[125] Flaherty K.T., Infante J.R., Daud A. (2012). Combined BRAF and MEK inhibition in melanoma with BRAF V600 mutations. N Engl J Med.

[126] Long G.V., Stroyakovskiy D., Gogas H. (2015). Dabrafenib and trametinib versus dabrafenib and placebo for Val600 BRAF-mutant melanoma: a multicentre, double-blind, phase 3 randomised controlled trial. Lancet.

[127] Alterio D., Marvaso G., Ferrari A. (2016). Combination of dabrafenib and radiotherapy: could skin toxicity be affected by different irradiation techniques?. BJR Case Rep.

[128] Anker C.J., Ribas A., Grossmann A.H. (2013). Severe liver and skin toxicity after radiation and vemurafenib in metastatic melanoma. J Clin Oncol.

[129] Boussemart L., Boivin C., Claveau J. (2013). Vemurafenib and radiosensitization. JAMA Dermatol.

[130] Churilla T.M., Chowdhry V.K., Pan D., de la Roza G., Damron T., Lacombe M.A. (2013). Radiation-induced dermatitis with vemurafenib therapy. Pract Radiat Oncol.

[131] Houriet C., Klass N.D., Beltraminelli H., Borradori L., Oberholzer P.A. (2014). Localized epidermal cysts as a radiation recall phenomenon in a melanoma patient treated with radiotherapy and the BRAF inhibitor vemurafenib. Case Rep Dermatol.

[132] Kuo K.Y., Jiang W., Swetter S.M., Kwong B.Y. (2016). Enhanced radiation dermatitis associated with concurrent palliative radiation and vemurafenib therapy. Cutis.

[133] Lang N., Sterzing F., Enk A.H., Hassel J.C. (2014). Cutis verticis gyrata-like skin toxicity during treatment of melanoma patients with the BRAF inhibitor vemurafenib after whole-brain radiotherapy is a consequence of the development of multiple follicular cysts and milia. Strahlenther Onkol.

[134] Merten R., Hecht M., Haderlein M. (2014). Increased skin and mucosal toxicity in the combination of vemurafenib with radiation therapy. Strahlenther Onkol.

[135] Peuvrel L., Ruellan A.L., Thillays F. (2013). Severe radiotherapy-induced extracutaneous toxicity under vemurafenib. Eur J Dermatol.

[136] Pulvirenti T., Hong A., Clements A. (2016). Acute radiation skin toxicity associated With BRAF inhibitors. J Clin Oncol.

[137] Reigneau M., Granel-Brocard F., Geoffrois L. (2013). Efflorescence of scalp cysts during vemurafenib treatment following brain radiation therapy: a radiation recall dermatitis?. Eur J Dermatol.

[138] Rompoti N., Schilling B., Livingstone E. (2013). Combination of BRAF inhibitors and brain radiotherapy in patients with metastatic melanoma shows minimal acute toxicity. J Clin Oncol.

[139] Saco M., Mitchell C. (2014). Severe radiation dermatitis associated with concomitant vemurafenib therapy in a patient with metastatic melanoma. J Am Acad Dermatol.

[140] Satzger I., Degen A., Asper H., Kapp A., Hauschild A., Gutzmer R. (2013). Serious skin toxicity with the combination of BRAF inhibitors and radiotherapy. J Clin Oncol.

[141] Schulze B., Meissner M., Wolter M., Rodel C., Weiss C. (2014). Unusual acute and delayed skin reactions during and after whole-brain radiotherapy in combination with the BRAF inhibitor vemurafenib. Two case reports. Strahlenther Onkol.

[142] Strobel S.B., Patzold S., Zimmer L., Jensen A., Enk A., Hassel J.C. (2017). Radiosensitization by BRAF inhibitors. J Dtsch Dermatol Ges.

[143] Ueki K., Kosaka Y., Kimino G., Imagumbai T., Takayama K., Kokubo M. (2016). Treatment of malignant melanoma with nivolumab and vemurafenib combined with hypofractionated radiation therapy. Int Cancer Conf J.

[144] Yilmaz M., Celik U., Hascicek S. (2020). Radiation recall dermatitis with dabrafenib and trametinib: a case report. World J Clin Cases.

[145] Ziegler J.S., Kroeze S., Hilbers M.L. (2020). Toxicity of combined targeted therapy and concurrent radiotherapy in metastatic melanoma patients: a single-center retrospective analysis. Melanoma Res.

[146] Hecht M., Meier F., Zimmer L. (2018). Clinical outcome of concomitant vs interrupted BRAF inhibitor therapy during radiotherapy in melanoma patients. Br J Cancer.

[147] Gaudy-Marqueste C., Carron R., Delsanti C. (2014). On demand Gamma-Knife strategy can be safely combined with BRAF inhibitors for the treatment of melanoma brain metastases. Ann Oncol.

[148] Tetu P., Allayous C., Oriano B. (2019). Impact of radiotherapy administered simultaneously with systemic treatment in patients with melanoma brain metastases within MelBase, a French multicentric prospective cohort. Eur J Cancer.

[149] Ahmed K.A., Freilich J.M., Sloot S. (2015). LINAC-based stereotactic radiosurgery to the brain with concurrent vemurafenib for melanoma metastases. J Neurooncol.

[150] Stera S., Balermpas P., Blanck O. (2019). Stereotactic radiosurgery combined with immune checkpoint inhibitors or kinase inhibitors for patients with multiple brain metastases of malignant melanoma. Melanoma Res.

[151] Patel B.G., Ahmed K.A., Johnstone P.A., Yu H.H., Etame A.B. (2016). Initial experience with combined BRAF and MEK inhibition with stereotactic radiosurgery for BRAF mutant melanoma brain metastases. Melanoma Res.

[152] Stefan D., Popotte H., Stefan A.R. (2016). Vemurafenib and concomitant stereotactic radiation for the treatment of melanoma with spinal metastases: a case report. Rep Pract Oncol Radiother.

[153] Marti F.E.M., Jayson G.C., Manoharan P. (2019). Novel phase I trial design to evaluate the addition of cediranib or selumetinib to preoperative chemoradiotherapy for locally advanced rectal cancer: the DREAMtherapy trial. Eur J Cancer.

[154] Wu C., Williams T.M., Robb R. (2020). Phase I trial of trametinib with neoadjuvant chemoradiation in patients with locally advanced rectal cancer. Clin Cancer Res.

[155] FDA (2020). Highlights of prescribing information: Koselugo (selumetinib).

[156] U.S. Food and Drug Administration (2020). Highlights of prescribing information: Mekinist (trametinib).

[157] Ghia A.J., Tward J.D., Anker C.J., Boucher K.M., Jensen R.L., Shrieve D.C. (2014). Radiosurgery for melanoma brain metastases: the impact of hemorrhage on local control. J Radiosurg SBRT.

[158] Raizer J.J., Hwu W.J., Panageas K.S. (2008). Brain and leptomeningeal metastases from cutaneous melanoma: survival outcomes based on clinical features. Neuro Oncol.

[159] Kotecha R., Miller J.A., Venur V.A. (2018). Melanoma brain metastasis: the impact of stereotactic radiosurgery, BRAF mutational status, and targeted and/or immune-based therapies on treatment outcome. J Neurosurg.

[160] Gatterbauer B., Hirschmann D., Eberherr N. (2020). Toxicity and efficacy of Gamma Knife radiosurgery for brain metastases in melanoma patients treated with immunotherapy or targeted therapy-a retrospective cohort study. Cancer Med.

[161] Ahmed K.A., Abuodeh Y.A., Echevarria M.I. (2016). Clinical outcomes of melanoma brain metastases treated with stereotactic radiosurgery and anti-PD-1 therapy, anti-CTLA-4 therapy, BRAF/MEK inhibitors, BRAF inhibitor, or conventional chemotherapy. Ann Oncol.

[162] Xu Z., Lee C.C., Ramesh A. (2017). BRAF V600E mutation and BRAF kinase inhibitors in conjunction with stereotactic radiosurgery for intracranial melanoma metastases. J Neurosurg.

[163] Ly D., Bagshaw H.P., Anker C.J. (2015). Local control after stereotactic radiosurgery for brain metastases in patients with melanoma with and without BRAF mutation and treatment. J Neurosurg.

[164] Patel K.R., Chowdhary M., Switchenko J.M. (2016). BRAF inhibitor and stereotactic radiosurgery is associated with an increased risk of radiation necrosis. Melanoma Res.

[165] Couty E., Vallard A., Sotton S. (2019). Safety assessment of anticancer drugs in association with radiotherapy in metastatic malignant melanoma: a real-life report: radiation/systemic drug combo in metastatic melanoma. Cancer Chemother Pharmacol.

[166] Liebner D.A., Walston S.A., Cavaliere R. (2014). Radiation necrosis mimicking rapid intracranial progression of melanoma metastasis in two patients treated with vemurafenib. Melanoma Res.

[167] Marquez-Rodas I., Aviles-Izquierdo J.A., Parra V. (2016). Exclusion criteria vs reality: dual BRAF/MEK inhibition and radiotherapy in a patient with melanoma metastatic to the brain and ECOG 3. Tumori.

[168] Flaum N., Lorigan P., Whitfield G.A., Hawkins R.E., Pinkham M.B. (2016). Integrating radiation therapy with emerging systemic therapies: lessons from a patient with cerebral radionecrosis, spinal cord myelopathy, and radiation pneumonitis. Pract Radiat Oncol.

[169] Ahmad S.S., Crittenden M.R., Tran P.T. (2019). Clinical Development of novel drug-radiotherapy combinations. Clin Cancer Res.

[170] Sharma R.A., Plummer R., Stock J.K. (2016). Clinical development of new drug-radiotherapy combinations. Nat Rev Clin Oncol.

