# Supplementary material for: ESMO-ESTRO consensus statements on the safety of combining radiotherapy with EGFR, ALK, or BRAF/MEK inhibitors
Source: ESMO Open. 2026 Feb 26;11(3):106076. doi: 10.1016/j.esmoop.2026.106076 (PMC12955645; doi:10.1016/j.esmoop.2026.106076)
Supplement: Supplementary Figures [file mmc1.pdf]

Supplementary Figures

This document provides additional data for the ESMO-ESTRO consensus statements on the safety of combining radiotherapy with EGFR, ALK, or BRAF/MEK inhibitors.

Table of Contents

Figure S1. PRISMA flow diagram for EGFR inhibitors. 2
Figure S2. PRISMA flow diagram for ALK inhibitors. 3
Figure S3. PRISMA flow diagram for BRAF/MEK inhibitors. 4
Figure S4. Additional selection step for EGFR inhibitors. 5
Figure S5. Delphi statement flow diagram for EGFR inhibitors. 6
Figure S6. Delphi statement flow diagram for ALK inhibitors. 7
Figure S7. Delphi statement flow diagram for BRAF/MEK inhibitors. 8

Figure S1. PRISMA flow diagram for EGFR inhibitors.

Original search date: 26 March 2021.

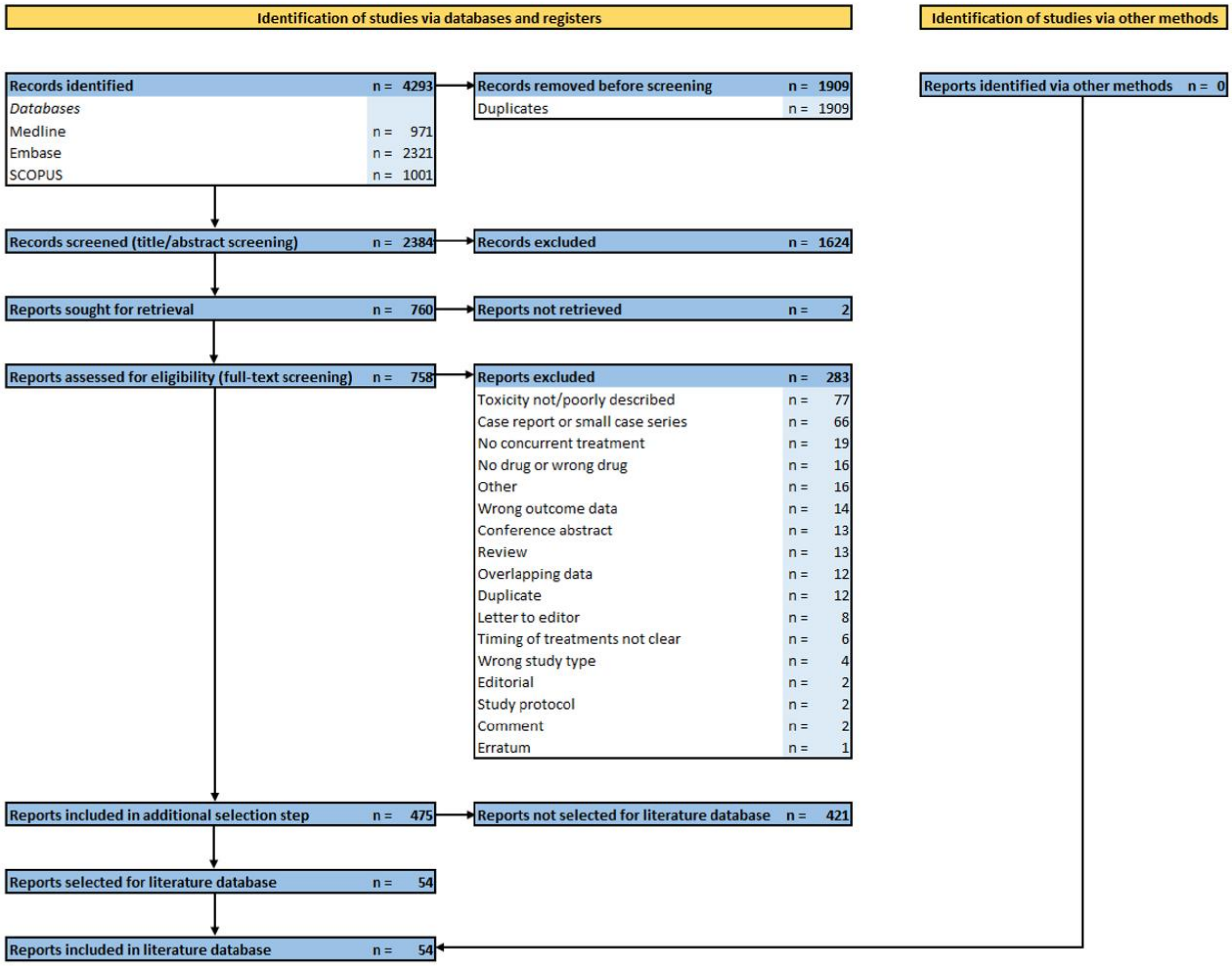

Figure S2. PRISMA flow diagram for ALK inhibitors.

Original search date: 21 December 2020.

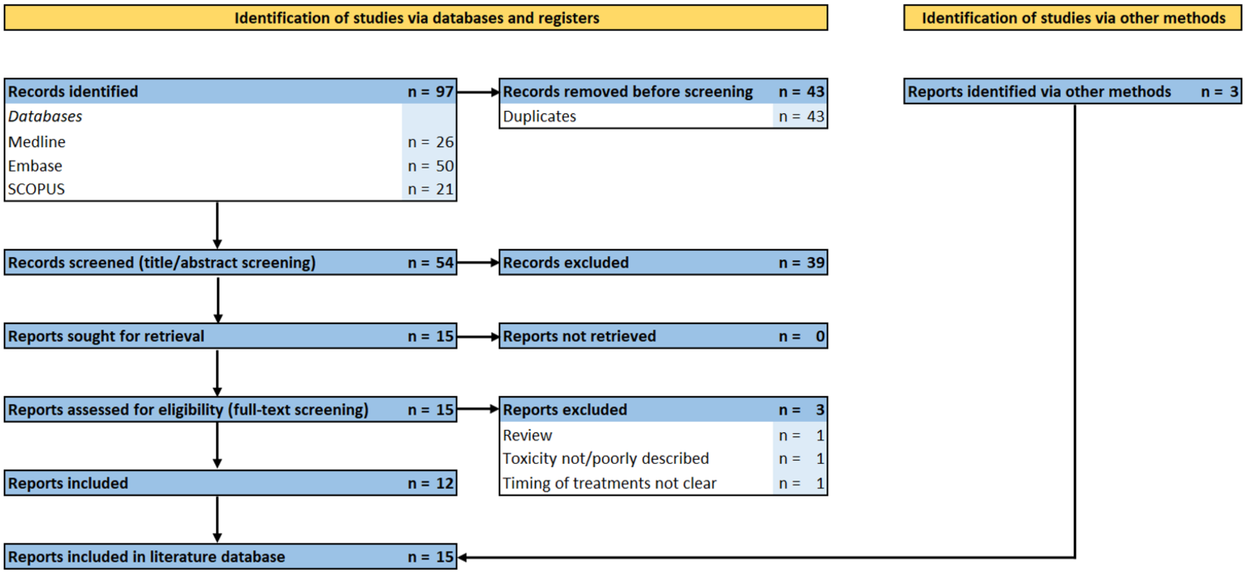

Figure S3. PRISMA flow diagram for BRAF/MEK inhibitors.

Original search date: 21 December 2020.

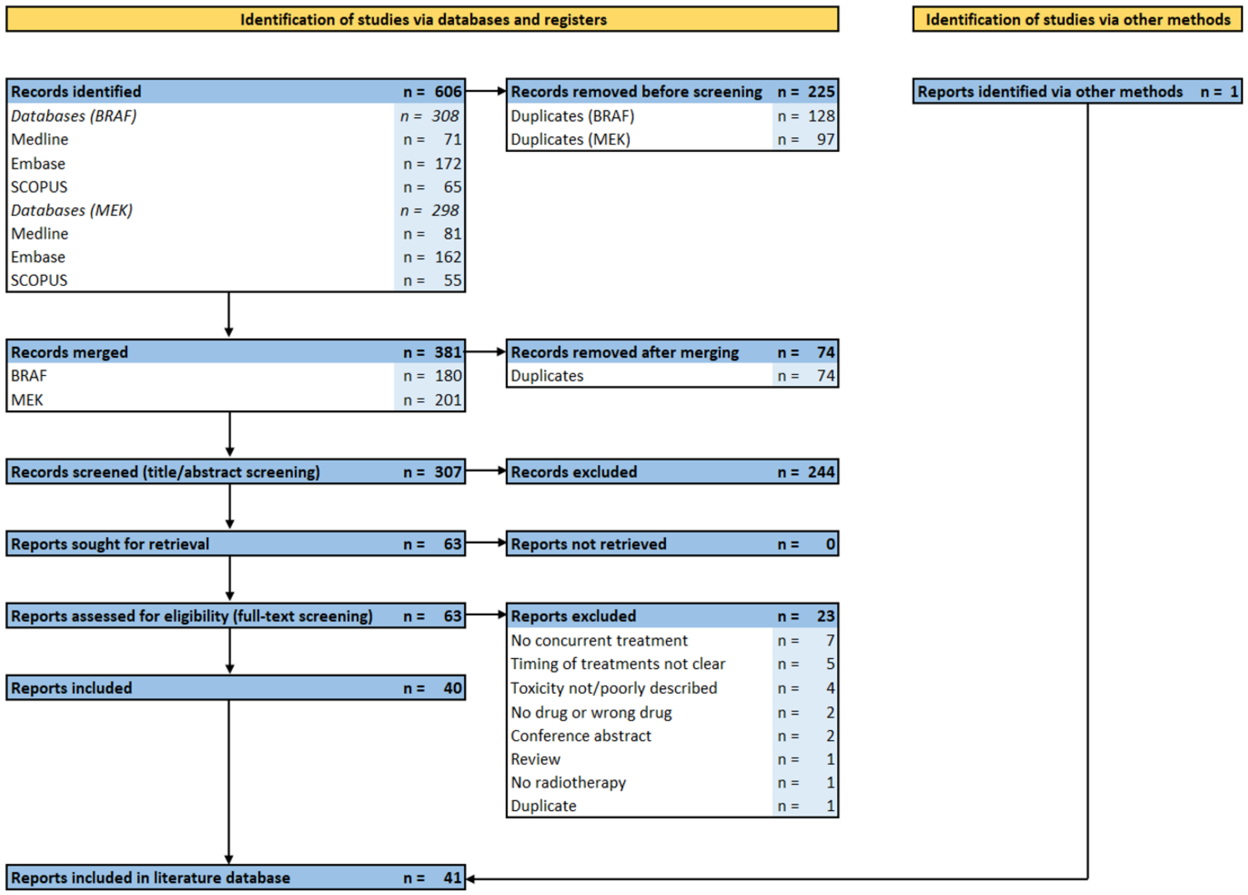

Figure S4. Additional selection step for EGFR inhibitors.

**Additional selection step for 3 large reviews: immunotherapy, VEGF inhibitors, EGFR inhibitors.**	
**Step 1**	**All reports were prioritized according to the following priorities:**<br>**Priority 1:** Study type: phase III > phase II > phase I > prospective cohort > retrospective cohort > cases<br>**Priority 2:** Number of patients: prioritized from large to small studies<br>**Priority 3:** Year of publication: new publications prioritized
**Step 2**	**For each irradiated tissue type, both high-dose and low-dose RT, as well as the different drug subtypes were considered separate scenarios. This resulted in 24 scenarios for immunotherapy and 20 for VEGF inhibitors (shown below).**
**Step 3**	**For each scenario, the first 3 studies/trials were selected.**
**Step 4**	**On top of that, the most recent meta-analyses were added.**

**The following scenarios were defined, based on irradiated tissue, drug subtype and radiotherapy dose:**						
**EGFR inhibitors:**	BRAIN_ANTIBODY_LOW	BRAIN_ANTIBODY_HIGH	BRAIN_TKI1_LOW	BRAIN_TKI1_HIGH	BRAIN_TKI23_LOW	BRAIN_TKI23_HIGH
	HN_ANTIBODY_LOW	HN_ANTIBODY_HIGH	HN_TKI1_LOW	HN_TKI1_HIGH	HN_TKI23_LOW	HN_TKI23_HIGH
	THORAX_ANTIBODY_LOW	THORAX_ANTIBODY_HIGH	THORAX_TKI1_LOW	THORAX_TKI1_HIGH	THORAX_TKI23_LOW	THORAX_TKI23_HIGH
	ABDOMEN/PELVIS_ANTIBODY_LOW	ABDOMEN/PELVIS_ANTIBODY_HIGH	ABDOMEN/PELVIS_TKI1_LOW	ABDOMEN/PELVIS_TKI1_HIGH	ABDOMEN/PELVIS_TKI23_LOW	ABDOMEN/PELVIS_TKI23_HIGH
	VARIOUS_ANTIBODY_LOW	VARIOUS_ANTIBODY_HIGH	VARIOUS_TKI1_LOW	VARIOUS_TKI1_HIGH	VARIOUS_TKI23_LOW	VARIOUS_TKI23_HIGH

Abbreviations:
LOW: low radiotherapy dose
HIGH: high radiotherapy dose
TKI1: 1st generation tyrosine kinase inhibitor
TKI23: 2nd/3rd generation tyrosine kinase inhibitor
HN: head and neck

Figure S5. Delphi statement flow diagram for EGFR inhibitors.

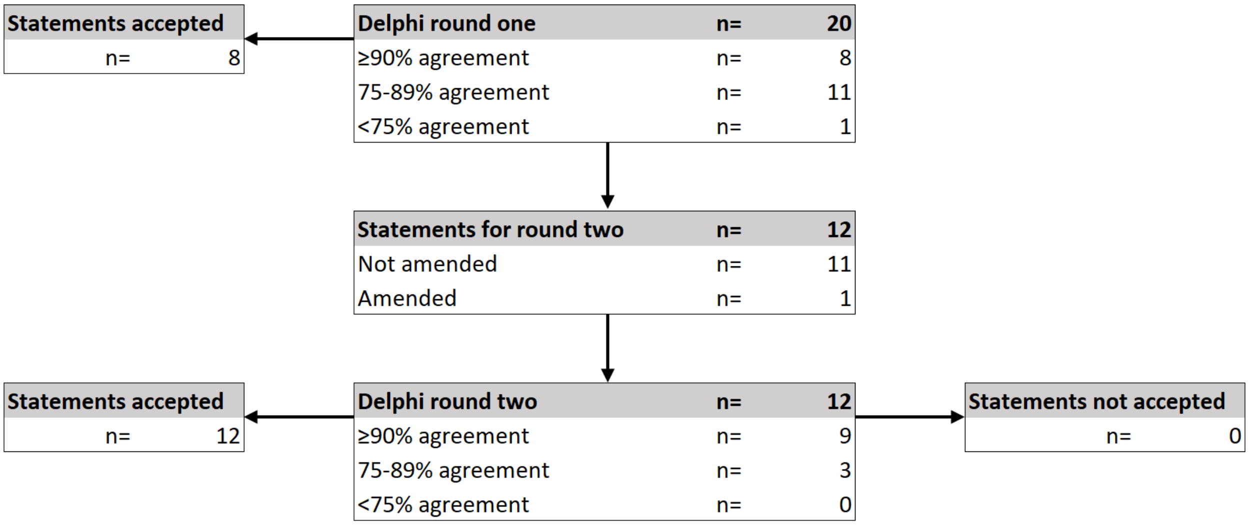

Figure S6. Delphi statement flow diagram for ALK inhibitors.

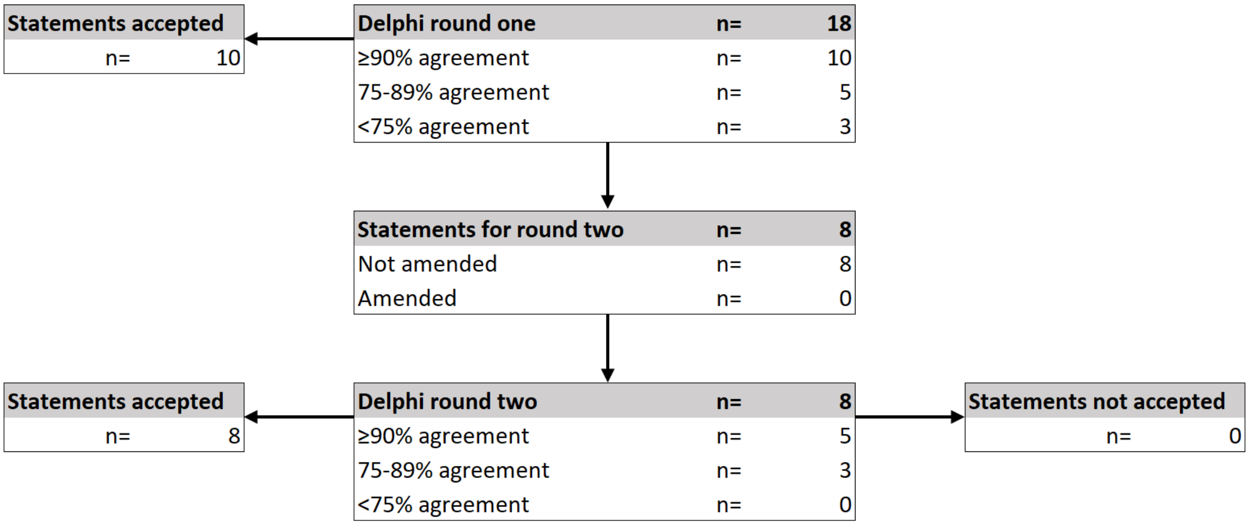

Figure S7. Delphi statement flow diagram for BRAF/MEK inhibitors.

Delphi round one	n=	19
≥90% agreement	n=	18
75-89% agreement	n=	1
<75% agreement	n=	0

Statements accepted	
n=	18

Statements for round two	n=	1
Not amended	n=	1
Amended	n=	0

Delphi round two	n=	1
≥90% agreement	n=	1
75-89% agreement	n=	0
<75% agreement	n=	0

Statements accepted	
n=	1

Statements not accepted	
n=	0
