# Supplementary material for: ESMO-ESTRO consensus statements on the safety of combining radiotherapy with EGFR, ALK, or BRAF/MEK inhibitors
Source: ESMO Open. 2026 Feb 26;11(3):106076. doi: 10.1016/j.esmoop.2026.106076 (PMC12955645; doi:10.1016/j.esmoop.2026.106076)
Supplement: Supplementary Material [file mmc2.pdf]

Supplementary Material

This document provides additional data for the ESMO-ESTRO consensus statements on the safety of combining radiotherapy with EGFR, ALK, or BRAF/MEK inhibitors.

Table of Contents

EGFR inhibitors. General information and systematic literature review. 2

ALK inhibitors. General information and systematic literature review. 14

BRAF and MEK inhibitors. General information and systematic literature review. 19

EGFR (epidermal growth factor receptor) inhibitors

1. Drug information

Name	Drug type	$T_{1/2}$ (± SD)	$T_{1/2}$ x 5	Weblinks
Cetuximab *Erbitux*	mAb	112h	23d	FDA
Panitumumab *Vectibix*	mAb	7.5d	37.5d	FDA
Necitumumab *Portrazza*	mAb	14d	70d	FDA
Erlotinib *Tarceva*	Small-molecule	36.2h	7.5d	FDA
Gefitinib *Iressa*	Small-molecule	48h	10d	FDA
Osimertinib *Tagrisso*	Small-molecule	48h	10d	FDA
Afatinib *Giotrif, gilotrif*	Small-molecule	37h	7.7d	FDA
Icotinib *Conmana*	Small-molecule	5.5h	27.5h	PubChem

mAb = monoclonal antibody; $T_{1/2}$ = plasma half-life; h = hours; d = days; m = minutes; SD = standard deviation.

2. Biological & pharmacological data

2.1 Pathway & drug mechanism

Binding of a ligand to an EGFR leads to structural changes, promoting receptor dimerization and subsequently activation by phosphorylation of the cytoplasmic tail of the receptor [1]. EGFR activation leads to downstream upregulation of several signaling pathways that are involved in cell survival and proliferation, including the MAPK and PI3K pathways [1, 2]. Upregulated EGFR signaling is a common feature of many tumors, making the inhibition of EGFRs an effective target, in e.g. head and neck cancer (also in combination with RT [3]), colorectal cancer and lung cancer [2]. EGFR inhibition influences DNA repair and cell cycle arrest and can increase radiosensitivity to some extent [2, 4-8].

2.2 Organ-specific availability

Blood-brain barrier penetration of many EGFR TKIs is generally poor, but significantly better for osimertinib [9-12]. Also afatinib has shown to penetrate the blood-brain barrier [11, 12].

3. Literature review

General summary

Data regarding the combination of RT and EGFR inhibitors clearly demonstrate an increased risk of dermatitis, skin rash and mucositis. In addition, EGFR inhibitors possibly increase pneumonitis risk and they may modestly increase (lower) GI-related and brain-related toxicity, when combined with RT. However, most studies consider these combinations feasible.

Skin

A large amount of high-quality data shows a markedly increased risk of high-grade dermatitis and skin rash when RT is combined with EGFR inhibitors. Most data are derived from head and neck cancer trials. The most relevant meta-analyses are summarized below.

A **meta-analysis** comparing cisplatin-based CRT +/- cetuximab in 1744 nasopharyngeal carcinoma patients, shows significantly higher G3-4 dermatitis (RR 6.41, based on all patients) and skin rash (RR 38.09, based on 417 patients) in patients receiving cetuximab [13]. A **meta-analysis** about combining EGFR TKIs (gefitinib and erlotinib) and thorax (C)RT reports G1-2 rash in 42% (RT) and 67% (CRT), but does not analyze dermatitis [14]. In a different (slightly older) **meta-analysis** in nasopharyngeal carcinoma patients, the RR of skin rash (grade not mentioned) was 1.46, but not statistically significant [15]. A **meta-analysis** comparing cetuximab/nimotuzumab + RT with cisplatin-based CRT in nasopharyngeal cancer patients shows a signficantly higher risk of G3-4 skin rash (RR 6.45), but a subgroup analysis showed that only cetuximab and not nimotuzumab signficantly increased this risk (RR 11.41 vs. RR 1.32, respectively) [16]. Another, earlier **meta-analysis** analyzing the addition of EGFR inhibitors to RT (primarily head & neck), shows significantly higher rates of G3-4 radiation dermatitis (31% vs. 13%) and G3-4 rash (16% vs. 5%) [17].

A **meta-analysis** about the combination of cetuximab or panitumumab with (C)RT as neoadjuvant therapy in KRAS-wild type locally advanced rectal cancer patients, reports G3-4 hand-foot syndrome in 2% and G3-4 acneiform rash in 15% [18]. Increased risk of G3-4 rash was also observed in a **meta-analysis** comparing whole brain RT+erlotinib/gefitinib with whole brain RT alone (OR 7.96 (p=0.003) [19]. In a **meta-analysis** analyzing whole brain RT/stereotactic RT for NSCLC brain metastases, risk of G≥3 rash (42% vs. 7%) and dry skin (16% vs. 1%) were increased in patients receiving the combination of RT with EGFR TKIs, compared to RT alone [20]. Another **meta-analysis** in the same patient category also showed significantly increased risks of all-grade rash (RR 5.0, p<0.001) and dry skin (RR 8.4, p=0.017) [21].

Brain

Most studies describe the addition of EGFR TKIs (and not monoclonal antibodies) to brain RT. Several studies suggest an increased risk of toxicities when EGFR inhibitors are combined with RT, but most RT-related toxicities are moderately or insignificantly increased. The most relevant clinical data are summarized below.

In a **meta-analysis** comparing whole brain RT + erlotinib/gefitinib with whole brain RT alone, the rates of G3-4 dyspnea (OR 1.09), fatigue (OR 0.69), diarrhea (OR 1.37) and nausea/vomiting (OR 1.37) were not significantly different. However, there was a significantly lower risk of G3-4 myelosuppression in patients receiving combined therapy (OR 0.19, p=0.001) [19]. In a **meta-analysis** analyzing whole brain RT/stereotactic RT +/- EGFR TKIs for NSCLC brain metastases, the overall adverse event risk was higher in the RT+EGFR TKI group (20% vs. 12%, p=0.003), but the only significantly increased non-dermatological toxicity was G≥3 diarrhea (20% vs. 8%, p=0.02). Possibly RT-related toxicities, including G≥3 fatigue (21% vs. 13%), dizziness (26% vs. 19%) and nausea/vomiting (26% vs. 17%), occurred more often in the RT+EGFR TKI group, but the differences were not statistically significant [20]. In another **meta-analysis** in the same patient category, no significant differences were seen in non-dermatological adverse events of any grade [21].

A **phase III** trial comparing whole brain RT + SRS +/- erlotinib (150 mg q.d.) for NSCLC brain metastases showed a significantly higher risk of G≥3 toxicity in the erlotinib arm (n=41): 49% vs. 11% (p<0.001). The G≥3 toxicity rate in a third arm (n=40), with temozolomide instead of erlotinib, was 41%. Grade 4-5 toxicities were only observed in the erlotinib and temozolomide arms. In the erlotinib arm, G4 myocardial ischemia, G4 brain necrosis and G5 hemorrhagic stroke (all n=1) were reported. In the temozolomide arm, G4 cytopenia, G4 hypokalemia and G5 thrombocytopenia (all n=1). The frequency of specific G3 toxicities was not reported [22]. In a **phase III** trial adding nimotuzumab (150 mg/m^2 weekly) to RT for diffuse intrinsic pontine glioma (n=42), most common drug-related toxicities were alopecia (14%), vomiting, headache and radiation skin injury (all 7%) [23].

The RTOG 0211 **phase I/II** trial showed high, but not unexpected toxicity rates (G3 46%, G4 18%, G5 1%) in 136 glioblastoma patients receiving gefitinib (500 mg q.d.) combined with RT. Grade 3-4 dermatological toxicity was observed in 14%, neurological toxicity in 13%, and constitutional symptoms in 9% [24]. A **randomized phase II** study adding gefitinib (250 mg q.d., n=16) or temozolomide (n=43) to whole brain RT for NSCLC brain metastases showed the following G3-4 toxicities in the gefitinib arm: G4 fatigue (n=1), G3

fatigue (n=2), G3 diarrhea, G3 mucositis and G3 dyspnea (all n=1). In the gefitinib arm, 3 patients discontinued therapy due to toxicity (asthenia, mucositis and diarrhea). Quality of life and cognitive function were analyzed, but not compared between the two treatment arms [25]. A **phase II** trial adding erlotinib (100 mg or 200 mg q.d.) to temozolomide-based CRT in 65 patients with glioblastoma and gliosarcoma did not report unexpected toxicities [26]. Also adding nimotuzumab (200 mg weekly) to temozolomide-based CRT in 36 glioblastoma patients, was found to be tolerable in another **phase II** trial [27]. Furthermore, adding cetuximab (250 mg/m^2 weekly) to RT for diffuse pontine gliomas and high-grade astrocytomas did not lead to unexpected toxicities in a **phase II** study with 45 pediatric patients [28]. A placebo-controlled, **randomized phase II** trial adding erlotinib (100 mg q.d.) to whole brain RT in patients with multiple NSCLC brain metastases, did not show increased non-dermatological G3-4 toxicity in the erlotinib arm (n=40), compared to placebo (n=40) and an even lower rate of G3-4 fatigue (17.5% vs. 35%). Quality of life scores at 1 and 2 months after RT were comparable between the two arms [29]. A **phase I** study evaluating the addition of icotinib (125-625 mg t.i.d.) to whole brain RT in 15 patients with NSCLC brain metastases, showed a recommended dose of 375 mg t.i.d. Only at 500 mg t.i.d., G3 toxicity was observed (nausea in 2/6 and alanine aminotransferase elevation in 1/6 patients). Within the short follow-up of 20 weeks, no deterioration of neurocognitive function (MMSE) was observed. Whole brain RT did not increase the blood-brain barrier penetration of icotinib [30].

A large **prospective** study did not show significant toxicity differences between patients with lung adenocarcinoma brain metastases receiving Gamma Knife radiosurgery with (n=238) or without (n=370) EGFR TKI concurrently or after SRS. In both groups together, SRS-related G3-4 toxicity was observed in only 2%, but a non-significant trend was seen towards more SRS-related (all-grade) toxicity in the EGFR TKI group (HR 1.72, p=0.097) [31].

Some small, **retrospective** studies did not report increased toxicity when combining EGFR TKIs with intracranial RT [32-35].

Head and neck

A reasonable amount of randomized studies and meta-analyses show that combining EGFR antibodies (particularly cetuximab) with head and neck RT significantly increases the risk of G≥3 mucositis. Less data are available about EGFR TKI combinations, but some show increased G≥3 mucositis risks as well.

<u>EGFR antibodies + RT</u>

A large Asian **meta-analysis** of 1239 patients compared EGFRi (cetuximab or nimotuzumab) + RT to cisplatin-based CRT. The EGFRi group exhibited a lower risk of experiencing G3–4 toxic events, including anemia (RR 0.11), neutropenia (RR 0.23), thrombocytopenia (RR 0.31) and vomiting (RR 0.04). The risk of G3-4 mucositis was significantly higher in the EGFRi group than in the cisplatin group (RR 1.24). However, significant heterogeneity was observed and sensitivity analysis led to the exclusion of one trial, resulting in a non-significant trend towards a higher G3-4 mucositis risk in the EGFRi group (RR 1.13). Subgroup analysis showed that the G3-4 mucositis risk was increased only in the cetuximab group (RR 1.62), but not in the nimotuzumab group (RR 0.92) [16].

An earlier **meta-analysis** from 2009 reporting on 14 trials (primarily head & neck) of EGFRi + RT versus RT alone, found significantly higher G3-4 mucositis rates in the EGFRi + RT group, compared to RT alone (47% vs. 27%, respectively) with an RR of 1.8 [17].

More recent data from the ARTSCAN III **phase III** trial, comparing cisplatin + RT (n=145) with cetuximab + RT (n=146) in locoregionally advanced head and neck cancer patients, showed significantly more acute G3-4 nausea (28% vs. 11%), vomiting (5% vs. 0%), acute kidney injury (13% vs. 1%), neutropenia (11% vs. 1%), tinnitus (10% vs. 1%), and dysphagia (32% vs. 21%) in the cisplatin arm, but more acute G3-4 mucositis (72% vs. 60%), skin reactions (22% vs. 8%), and acneiform rash (21% vs. 0%) in the cetuximab arm. Regarding late toxicities, significantly more G3 taste alteration (15% vs. 5%) and G3-4 hearing impairment (8% vs. 2%) were observed in the cisplatin arm, but significantly more G3 pain (19% vs. 10%) and G3-4 oral mucosa status (4% vs. 0%) were observed in the cetuximab arm. Overall toxicity did not differ significantly between the two treatment groups [36]. A **phase II-III trial** adding induction chemotherapy with docetaxel, cisplatin and fluorouracil to cisplatin-based CRT or to cetuximab-based RT, only reported a slight, significant increase of G3-4 neutropenia (4% vs. 1%) in the induction chemotherapy arms (n=183), compared with controls (n=201), but no other increases in toxicity [37].

EGFR antibodies + CRT

A **meta-analysis** comparing cisplatin-based CRT +/- cetuximab in 1744 nasopharyngeal carcinoma patients, showed a significantly increased risk of G3-4 mucositis (RR 2.75) upon adding cetuximab. There was no significant increase in hematological or gastrointestinal toxicity [13]. Another **meta-analysis** of eight studies (711 participants) showed a significantly reduced myelosuppression rate when cetuximab is added to cisplatin-based CRT (RR 0.77). Rates of mucositis and gastrointestinal reactions were not significantly different [15]. A significant increase in G≥3 mucositis (67% vs. 56%) was seen in a randomized **phase III** trial comparing nimotuzumab + cisplatin-based CRT (n=267) with CRT alone (n=267). The rate of hospitalizations due to toxicities was borderline significantly higher in the nimotuzumab arm (22% vs. 15%). Some slight, non-significant differences regarding late G≥3 toxicities were observed, including xerostomia (4% vs. 2%) and dysphagia (6% vs. 3%) [38].

EGFR TKIs + CRT

The data for EGFR TKI combinations with RT are predominantly from phase II trials. A randomized **phase II** trial with 226 patients evaluating the addition of gefitinib (250-500 mg q.d.) to cisplatin-based CRT showed no major differences in the overall incidence of common CRT-associated toxicities, including mucositis, dysphagia, and dry mouth. There was a trend for an increase in G≥3 mucositis (all causes) in the gefitinib arms (46%), compared to placebo (36%). Radiation mucositis occurrred in 20-27% and 13%, respectively, but no increased incidence of G≥3 pharyngitis was observed (9% vs. 8%, respectively) [39]. Another randomized **phase II** trial with 67 patients adding gefitinib (250 mg q.d.) to cisplatin-based CRT also showed no major CRT-related toxicity differences [40]. In a non-randomized **phase II** trial (n=43), combining docetaxel and erlotinib (150 mg q.d.) with RT for locally advanced head and neck cancer appeared tolerable. Most common G3-4 toxicities were lymphopenia (95%), dysphagia (49%), radiation dermatitis (37%) and oral mucositis (35%) [41]. Studies with next-generation TKIs are less developed and still at a dose finding phase. In a **phase I** study, the combination of afatinib (20-40 mg q.d.) and docetaxel with RT in 27 patients with resected head and neck cancer was considered poorly tolerable, primarily due to dose-limiting mucositis. Grade 3 mucositis was observed in 63% [42].

Thorax

EGFR inhibitors may modestly increase the risk of RT toxicities when combined with thoracic RT, primarily the risk of radiation pneumonitis. In particular, one retrospective trial shows a high radiation pneumonitis risk when RT is combined with osimertinib. The effect of EGFR inhibitors on RT-related esophageal toxicity appears limited.

EGFR antibodies

The long-term results of the randomized **phase III** RTOG-0617 trial for unresectable stage III NSCLC demonstrated significantly higher overall treatment-related G≥3 toxicity in the CRT + cetuximab arms (87%), compared to no cetuximab (71%). Grade 5 events that were possibly, probably or definitely related to treatment occurred in 5/259 patients without cetuximab, and in 12/237 patients with cetuximab and were primarily pulmonary in nature [43]. The use of IMRT in 47% of the trial population was associated with lower rates of G≥3 pneumonitis (7.9% vs. 3.5%) even when stratified by cetuximab allocation [44]. Grade ≥3 pneumonitis risk was 4.7% without cetuximab and 7.6% with cetuximab and G≥3 esophagitis risk was 10.5% without cetuximab and 11.8% with cetuximab (significance not analyzed). Patients receiving high-dose RT (with/without cetuximab) experienced more G≥3 esophagitis than with standard-dose RT [45].

A randomized **phase II/III** trial analyzing the addition of cetuximab (400mg/m^2 first infusion, followed by 250mg/m^2 weekly) to CRT with cisplatin and capecitabine in 258 patients with esophageal cancer showed significantly more G3-4 non-hematological toxicity in the cetuximab-arm (79% vs. 63%). The largest G3-4 non-hematological toxicity increases were dermatological (22% vs. 4%), biochemical (24% vs. 11%), cardiac (6% vs. 2%) and pulmonary (6% vs. 3%). GI toxicity was similar in both groups [46]. No late G≥3 toxicity was observed with cetuximab, but some cases of G3 toxicity were observed in the CRT-alone arm (primarily esophageal toxicity), possibly because chemotherapy compliance was higher in the CRT-alone arm [47].

A randomized **phase III** trial (n=300) analyzing the addition of cetuximab (250 mg/m^2 weekly) to induction chemotherapy and preoperative CRT with cisplatin and docetaxel for resectable esophageal cancer unexpectedly showed significantly less G≥3 dysphagia in the cetuximab arm (13%), compared to controls

(27%). Also G≥3 esophagitis risk was lower with cetuximab (9% vs. 14%). Other RT-related toxicities and postoperative complications were similar in both groups [48].
A randomized **phase II** trial compared the addition of erlotinib (150 mg q.d.) to RT with CRT (etoposide and cisplatin) with 20 patients in both arms. Generally, G3-4 toxicity was low. Only G3-4 radiation pneumonitis occurred in 17% with erlotinib vs. 0% with etoposide/cisplatin. No G3-4 radiation esophagitis was observed, but all-grade esophagitis occurred in 33% with erlotinib and 21% with etoposide/cisplatin. The etoposide/cisplatin arm experienced more hematological toxicity [49].
In a **meta-analysis** of 14 RCTs with 2975 patients, 290 patients received cetuximab combined with CRT, but no significant differences regarding overall SAEs and radiation pneumonitis were observed, when compared to the corresponding CRT-alone arms [50]. A **phase I** trial shows, apart from one G4 pneumonia with neutropenia, no non-hematological G≥3 toxicity when nimotuzumab (100-400 mg weekly) was combined with palliative RT for stage IIB–IV NSCLC [51].

<u>EGFR TKIs</u>
A **meta-analysis** of EGFR TKIs, given concurrently with (C)RT for locally advanced or metastatic NSCLC included 446 patients from 16 prospective trials with varying (C)RT schedules. Toxicities were generally mild. The most common adverse events related to TKI use were rash and diarrhea. When combined with TKIs, the G1-3 esophagitis risk was 32% with RT and 37% with CRT, whereas the G1-2 interstitial pneumonia risk was 12% with RT and 17% with CRT. No G≥4 esophagitis or interstitial pneumonia were reported [14].
In a **phase III** trial in esophageal cancer, patients were randomized to CRT with paclitaxel/cisplatin +/- erlotinib (150 mg q.d.). Apart from skin rash, severe toxicities were similar in both groups; 71% in the erlotinib arms (n=176) and 64% in the control arms (n=176). No G≥3 radiation pneumonitis was observed among all patients. Long-term follow up, with a minimum of 5 years, demonstrated no differences in cardiac disorders or esophageal stenosis between groups [52, 53]. A single-arm **phase II** trial combining palliative thoracic RT with erlotinib (150 mg q.d.) in 40 NSCLC patients had minimal safety concerns with 16 patients experiencing G≥3 toxicities, but the only *treatment-related* toxicities were G4 rash (n=1) and G3 nausea (n=1) [54]. In another **phase II** trial adding erlotinib (150 mg q.d.) to CRT with paclitaxel and carboplatin in 46 NSCLC patients, the only G≥3 non-dermatological toxicities were G4 pneumonitis (n=1), G3 pneumonitis (n=2) and G3 esophagitis (n=1) [55].
The ATOM **phase II** trial, (terminated early due to poor accrual) included patients on afatinib, erlotinib or gefitinib. TKIs were paused during RT. No G≥3 toxicities were reported in the 16 patients enrolled and one patient developed G2 pneumonitis [56].
Some **retrospective** cohorts also confirm that there are no significant concerns about increased RT toxicity when combining RT with erlotinib [34, 57], although one patient died due to interstitial pneumonitis [34]. Also for the combination of RT with gefitinib, **retrospective** data do not lead to safety concerns [32, 58]. Another **retrospective** trial including patients on different EGFR TKIs and with RT to several locations (including 11 lung lesions), reports one G≥3 radiation pneumonitis [35].
However, osimertinib, a third-generation TKI, has been reported to cause a potentially high rate of radiation pneumonitis when concurrently combined with conventional RT or stereotactic RT. Although **retrospective** in nature with only 11 patients reported, pneumonitis was observed in all eleven patients on follow-up CT imaging. Six patients (55%) developed G≥3 radiation pneumonitis, including 1 (9%) case of fatal radiation pneumonitis. The median RT dose for all patients was 60 Gy (range 30–64 Gy) and the median dose per fraction was 2 Gy (range 2–5 Gy). The median lung dose was 6.4 Gy [59].

Abdomen/pelvis
The reported evidence on toxicity of anti-EGFR drugs in combination with RT is grouped into two different pathologies, pancreatic cancer and rectal cancer. Toxicity is slightly increased, but considered acceptable, when these drugs are combined with RT or CRT. Most studies lack a (C)RT-only control arm, but the available data indicate a modestly increased toxicity risk, particularly GI-related.

<u>Pancreas</u>
A **phase III** trial included 66 patients with induction and maintenance gemcitabine (1000 mg/m^2 weekly) and erlotinib (100 mg q.d.) combined with capecitabine-based CRT. During the induction phase patients treated with gemcitabine + erlotinib experienced significantly more G3-4 anemia, febrile neutropenia, diarrhea, and acneiform rash, compared to patients treated with gemcitabine alone. All progression-free and eligible

patients were subsequently randomized between chemotherapy (+/- erlotinib) and CRT (+/- erlotinib). Six percent of the CRT group had G3-4 nausea vs. none in the chemotherapy group (p = 0.008) and G3-4 diarrhea occurred in 5% of the CRT group vs. 1% in the chemotherapy group (n.s.), but there were no other clinically or statistically significant toxicity differences between both groups. However, since erlotinib was only used as induction and maintenance, it was paused some days before RT and resumed 15 days after RT in this study, reducing possible radiosensitisation risks from erlotinib [60, 61].

In a randomized **phase II** study comparing the combination of cetuximab (400 mg/m^2 first infusion, followed by 250 mg/m^2 weekly) vs. bevacizumab with CRT in patients with resected pancreatic adenocarcinoma, 65 patients were allocated to the cetuximab arm. In both arms, the most common G≥3 adverse events were neutropenia and leukocytopenia, with 43% and 37% in the cetuximab arm and 56% and 38% in the bevacizumab arm, respectively. Particularly G≥3 skin rash occurred more often in the cetuximab arm (18%) compared to the bevacizumab arm (0%). In the cetuximab arm, G≥3 nausea and diarrhea risks were 4% and 7%, respectively, compared to 3% and 2% in the bevacizumab arm. Grade ≥3 elevated ALT and AST were observed in the cetuximab arm (both 9%), but not in the bevacizumab arm. Both treatment arms were considered tolerable [62].

Another **phase II** study analyzed the efficacy of induction chemotherapy (n=25) with gemcitabine (100 mg/m^2 weekly) and erlotinib (100 mg q.d.) followed by CRT (25 x 1.8 Gy) with erlotinib (n=16). The only reported G≥3 toxicities during CRT were G3 neutropenia (12.5%) and G4 thrombocytopenia (6.3%). No other significant toxicities were described [63].

In a **phase I** trial evaluating CRT (up to 38 Gy in 2 Gy fractions) with gemcitabine (100 mg/m^2 weekly) and erlotinib (100 mg q.d.) in 20 patients, 2 patients treated with 34 Gy experienced a DLT (G3 nausea and vomiting) and 1 patient treated with 38 Gy developed a DLT (G4 fatigue). Furthermore, one patient at the 34 Gy dose level developed G3 neutropenia and cellulitis during week 8. The recommended phase II dose was 15 x 2 Gy. All-grade nausea, vomiting and infection occurred significantly more often with higher RT doses [64].

Rectum

A **meta-analysis** published by Zhong et al. about EGFR inhibitors for locally advanced rectal cancer, showed the following pooled estimates: G3-4 diarrhea (17%), G3-4 hand–foot syndrome (2%) and G3-4 acneiform rash (15%) [18].

In a neoadjuvant **phase II** trial of the combination of cetuximab (400 mg/m^2 first infusion, followed by 250 mg/m^2 weekly) plus 5-FU (225 mg/m^2 q.d.) and RT in 40 patients with locally advanced rectal cancer, the most frequent G3–4 adverse events were skin rash (7.5%), diarrhoea (7.5%) and hypersensitivity (7.5%) reactions. Dose reduction or interruption of treatment occurred in six patients (15%), mainly due to G3 acneiform rash and G3 GI toxicity (both n=2). However, treatment was resumed when toxicity was decreased to G≤1 in 4 patients. Two G4 toxicities were reported: one anaphylactic shock (during first cetuximab dose) and one G4 diarrhea (after last cetuximab dose) [65].

A **phase I/II** study of the combination of 5-FU (225 mg/m^2 q.d.) and gefitinib (250-500 mg q.d.) with RT (50.4 Gy in 28 fractions and intraoperative 10 Gy boost) in 39 rectal cancer patients reported that 62% of patients required a dose reduction of gefitinib, primarily due to toxicity. The most common acute G≥3 toxicities were GI toxicity (20.5%), skin toxicity (15.3%) and genitourinary toxicity (10.2%) [66]. All-grade late toxicity occurred in 74%, with particularly GI toxicity (56%), reproductive system toxicity (49%) and urinary system toxicity (28%). Late G≥3 toxicity occurred in 38%, with primarily reproductive system toxicity (28%) and GI toxicity (10%) [67].

The SWOG 0713 **phase II** trial included 83 patients with locally advanced rectal cancer, receiving oxaliplatin, capecitabine and cetuximab (400 mg/m^2 first infusion, followed by 250mg/m^2 weekly) as induction therapy and concurrently with RT (45-54 Gy in fractions of 1.8 Gy). Most common G≥3 toxicities were diarrhea (35%), rash/acne (12%), lymphopenia (11%), hypokalemia (11%) and nausea (9%). Grade 4 toxicity was observed in two patients: dehydration, and colitis with hyponatremia. One G5 multi-organ failure was considered possibly related to study treatment by the investigators [68].

The Expert-C randomized **phase II** clinical trial included 83 patients receiving oxaliplatin, capecitabine and cetuximab (400mg/m^2 first infusion, followed by 250mg/m^2 weekly) as induction, concurrently with RT and as adjuvant therapy. During RT, G≥3 diarrhea was increased with cetuximab (10%), compared to no cetuximab (1%). During adjuvant chemotherapy, risk of G≥3 diarrhea was also higher in the cetuximab arm (16% vs. 6%) [69].

Musculoskeletal/other

No publications were identified that are specific for this RT area.

References

[1] Sigismund S, Avanzato D, Lanzetti L. Emerging functions of the EGFR in cancer. Mol Oncol. 2018;12:3-20. DOI: 10.1002/1878-0261.12155.
[2] Wheeler DL, Dunn EF, Harari PM. Understanding resistance to EGFR inhibitors-impact on future treatment strategies. Nat Rev Clin Oncol. 2010;7:493-507. DOI: 10.1038/nrclinonc.2010.97.
[3] Bonner JA, Harari PM, Giralt J, Azarnia N, Shin DM, Cohen RB, et al. Radiotherapy plus cetuximab for squamous-cell carcinoma of the head and neck. N Engl J Med. 2006;354:567-78. DOI: 10.1056/NEJMoa053422.
[4] Brand TM, Iida M, Luthar N, Starr MM, Huppert EJ, Wheeler DL. Nuclear EGFR as a molecular target in cancer. Radiother Oncol. 2013;108:370-7. DOI: 10.1016/j.radonc.2013.06.010.
[5] Dittmann K, Mayer C, Rodemann HP. Inhibition of radiation-induced EGFR nuclear import by C225 (Cetuximab) suppresses DNA-PK activity. Radiother Oncol. 2005;76:157-61. DOI: 10.1016/j.radonc.2005.06.022.
[6] Horn D, Hess J, Freier K, Hoffmann J, Freudlsperger C. Targeting EGFR-PI3K-AKT-mTOR signaling enhances radiosensitivity in head and neck squamous cell carcinoma. Expert Opin Ther Targets. 2015;19:795-805. DOI: 10.1517/14728222.2015.1012157.
[7] Kriegs M, Gurtner K, Can Y, Brammer I, Rieckmann T, Oertel R, et al. Radiosensitization of NSCLC cells by EGFR inhibition is the result of an enhanced p53-dependent G1 arrest. Radiother Oncol. 2015;115:120-7. DOI: 10.1016/j.radonc.2015.02.018.
[8] Kriegs M, Kasten-Pisula U, Riepen B, Hoffer K, Struve N, Myllynen L, et al. Radiosensitization of HNSCC cells by EGFR inhibition depends on the induction of cell cycle arrests. Oncotarget. 2016;7:45122-33. DOI: 10.18632/oncotarget.9161.
[9] Colclough N, Chen K, Johnstrom P, Strittmatter N, Yan Y, Wrigley GL, et al. Preclinical Comparison of the Blood-brain barrier Permeability of Osimertinib with Other EGFR TKIs. Clin Cancer Res. 2021;27:189-201. DOI: 10.1158/1078-0432.CCR-19-1871.
[10] Varrone A, Varnas K, Jucaite A, Cselenyi Z, Johnstrom P, Schou M, et al. A PET study in healthy subjects of brain exposure of (11)C-labelled osimertinib - A drug intended for treatment of brain metastases in non-small cell lung cancer. J Cereb Blood Flow Metab. 2020;40:799-807. DOI: 10.1177/0271678X19843776.
[11] Hochmair M. Medical Treatment Options for Patients with Epidermal Growth Factor Receptor Mutation-Positive Non-Small Cell Lung Cancer Suffering from Brain Metastases and/or Leptomeningeal Disease. Target Oncol. 2018;13:269-85. DOI: 10.1007/s11523-018-0566-1.
[12] Shah R, Lester JF. Tyrosine Kinase Inhibitors for the Treatment of EGFR Mutation-Positive Non-Small-Cell Lung Cancer: A Clash of the Generations. Clin Lung Cancer. 2020;21:e216-e28. DOI: 10.1016/j.cllc.2019.12.003.
[13] Wang BC, Shi LL, Fu C, Zhou HX, Zhang ZJ, Ding Q, et al. A meta-analysis of cisplatin-based concurrent chemoradiotherapy with or without cetuximab for locoregionally advanced nasopharyngeal carcinoma. Medicine. 2019;98:e17486.
[14] Liu R, Wei S, Zhang Q, Zhang X, Luo H, Tian J, et al. Epidermal growth factor receptor tyrosine kinase inhibitors combined with thoracic radiotherapy or chemoradiotherapy for advanced or metastatic non-small cell lung cancer: A systematic review and meta-analysis of single-arm trials. Medicine (Baltimore). 2019;98:e16427. DOI: 10.1097/MD.0000000000016427.
[15] Wang N, Wang K, Song F, Liu Y. Cetuximab in combination with chemoradiotherapy for nasopharyngeal carcinoma: A meta-analysis. Indian J Cancer. 2018;55:196-200. DOI: 10.4103/ijc.IJC_446_17.
[16] Liang ZG, Lin GX, Ye JX, Li Y, Li L, Qu S, et al. Cetuximab or Nimotuzumab Versus Cisplatin Concurrent with Radiotherapy for Local-Regionally Advanced Nasopharyngeal Carcinoma: a Meta-analysis. Asian Pac J Cancer Prev. 2018;19:1397-404. DOI: 10.22034/APJCP.2018.19.5.1397.

[17] Tejwani A, Wu S, Jia Y, Agulnik M, Millender L, Lacouture ME. Increased risk of high-grade dermatologic toxicities with radiation plus epidermal growth factor receptor inhibitor therapy. Cancer. 2009;115:1286-99. DOI: 10.1002/cncr.24120.
[18] Zhong X, Zhou Y, Cui W, Su X, Guo Z, Hidasa I, et al. The Addition of EGFR Inhibitors in Neoadjuvant Therapy for KRAS-Wild Type Locally Advanced Rectal Cancer Patients: A Systematic Review and Meta-Analysis. Front Pharmacol. 2020;11:706. DOI: 10.3389/fphar.2020.00706.
[19] Zheng MH, Sun HT, Xu JG, Yang G, Huo LM, Zhang P, et al. Combining Whole-Brain Radiotherapy with Gefitinib/Erlotinib for Brain Metastases from Non-Small-Cell Lung Cancer: A Meta-Analysis. Biomed Res Int. 2016;2016:5807346. DOI: 10.1155/2016/5807346.
[20] Wang X, Xu Y, Tang W, Liu L. Efficacy and Safety of Radiotherapy Plus EGFR-TKIs in NSCLC Patients with Brain Metastases: A Meta-Analysis of Published Data. Transl Oncol. 2018;11:1119-27. DOI: 10.1016/j.tranon.2018.07.003.
[21] Jiang T, Min W, Li Y, Yue Z, Wu C, Zhou C. Radiotherapy plus EGFR TKIs in non-small cell lung cancer patients with brain metastases: an update meta-analysis. Cancer Med. 2016;5:1055-65. DOI: 10.1002/cam4.673.
[22] Sperduto PW, Wang M, Robins HI, Schell MC, Werner-Wasik M, Komaki R, et al. A phase 3 trial of whole brain radiation therapy and stereotactic radiosurgery alone versus WBRT and SRS with temozolomide or erlotinib for non-small cell lung cancer and 1 to 3 brain metastases: Radiation Therapy Oncology Group 0320. Int J Radiat Oncol Biol Phys. 2013;85:1312-8. DOI: 10.1016/j.ijrobp.2012.11.042.
[23] Fleischhack G, Massimino M, Warmuth-Metz M, Khuhlaeva E, Janssen G, Graf N, et al. Nimotuzumab and radiotherapy for treatment of newly diagnosed diffuse intrinsic pontine glioma (DIPG): a phase III clinical study. J Neurooncol. 2019;143:107-13. DOI: 10.1007/s11060-019-03140-z.
[24] Chakravarti A, Wang M, Robins HI, Lautenschlaeger T, Curran WJ, Brachman DG, et al. RTOG 0211: a phase 1/2 study of radiation therapy with concurrent gefitinib for newly diagnosed glioblastoma patients. Int J Radiat Oncol Biol Phys. 2013;85:1206-11. DOI: 10.1016/j.ijrobp.2012.10.008.
[25] Pesce GA, Klingbiel D, Ribi K, Zouhair A, von Moos R, Schlaeppi M, et al. Outcome, quality of life and cognitive function of patients with brain metastases from non-small cell lung cancer treated with whole brain radiotherapy combined with gefitinib or temozolomide. A randomised phase II trial of the Swiss Group for Clinical Cancer Research (SAKK 70/03). Eur J Cancer. 2012;48:377-84. DOI: 10.1016/j.ejca.2011.10.016.
[26] Prados MD, Chang SM, Butowski N, DeBoer R, Parvataneni R, Carliner H, et al. Phase II study of erlotinib plus temozolomide during and after radiation therapy in patients with newly diagnosed glioblastoma multiforme or gliosarcoma. J Clin Oncol. 2009;27:579-84. DOI: 10.1200/JCO.2008.18.9639.
[27] Du XJ, Li XM, Cai LB, Sun JC, Wang SY, Wang XC, et al. Efficacy and safety of nimotuzumab in addition to radiotherapy and temozolomide for cerebral glioblastoma: a phase II multicenter clinical trial. J Cancer. 2019;10:3214-23. DOI: 10.7150/jca.30123.
[28] Macy ME, Kieran MW, Chi SN, Cohen KJ, MacDonald TJ, Smith AA, et al. A pediatric trial of radiation/cetuximab followed by irinotecan/cetuximab in newly diagnosed diffuse pontine gliomas and high-grade astrocytomas: A Pediatric Oncology Experimental Therapeutics Investigators' Consortium study. Pediatr Blood Cancer. 2017;64. DOI: 10.1002/pbc.26621.
[29] Lee SM, Lewanski CR, Counsell N, Ottensmeier C, Bates A, Patel N, et al. Randomized trial of erlotinib plus whole-brain radiotherapy for NSCLC patients with multiple brain metastases. J Natl Cancer Inst. 2014;106. DOI: 10.1093/jnci/dju151.
[30] Zhou L, He J, Xiong W, Liu Y, Xiang J, Yu Q, et al. Impact of whole brain radiation therapy on CSF penetration ability of Icotinib in EGFR-mutated non-small cell lung cancer patients with

brain metastases: Results of phase I dose-escalation study. Lung Cancer. 2016;96:93-100. DOI: 10.1016/j.lungcan.2016.04.003.
[31] Yomo S, Serizawa T, Yamamoto M, Higuchi Y, Sato Y, Shuto T, et al. The impact of EGFR-TKI use on clinical outcomes of lung adenocarcinoma patients with brain metastases after Gamma Knife radiosurgery: a propensity score-matched analysis based on extended JLGK0901 dataset (JLGK0901-EGFR-TKI). Journal of Neuro-Oncology. 2019;145:151-7.
[32] Santarpia M, Altavilla G, Borsellino N, Girlando A, Mancuso G, Pergolizzi S, et al. High-dose Radiotherapy for Oligo-progressive NSCLC Receiving EGFR Tyrosine Kinase Inhibitors: Real World Data. In Vivo. 2020;34:2009-14. DOI: 10.21873/invivo.11999.
[33] Chen H, Wu A, Tao H, Yang D, Luo Y, Li S, et al. Concurrent versus sequential whole brain radiotherapy and TKI in EGFR-mutated NSCLC patients with brain metastasis: A single institution retrospective analysis. Medicine (Baltimore). 2018;97:e13014. DOI: 10.1097/MD.0000000000013014.
[34] Li L, Liu LY, Chen M, Xiao NJ, Zhang YW, Zhang Y, et al. A pilot study of conformal radiotherapy combined with erlotinib-based multimodality therapy in newly diagnosed metastatic non-small-cell lung cancer. Eur Rev Med Pharmacol Sci. 2015;19:1812-20.
[35] Wang Y, Li Y, Xia L, Niu K, Chen X, Lu D, et al. Continued EGFR-TKI with concurrent radiotherapy to improve time to progression (TTP) in patients with locally progressive non-small cell lung cancer (NSCLC) after front-line EGFR-TKI treatment. Clin Transl Oncol. 2018;20:366-73. DOI: 10.1007/s12094-017-1723-1.
[36] Gebre-Medhin M, Brun E, Engstrom P, Haugen Cange H, Hammarstedt-Nordenvall L, Reizenstein J, et al. ARTSCAN III: A Randomized Phase III Study Comparing Chemoradiotherapy With Cisplatin Versus Cetuximab in Patients With Locoregionally Advanced Head and Neck Squamous Cell Cancer. J Clin Oncol. 2021;39:38-47. DOI: 10.1200/JCO.20.02072.
[37] Ghi MG, Paccagnella A, Ferrari D, Foa P, Alterio D, Codeca C, et al. Induction TPF followed by concomitant treatment versus concomitant treatment alone in locally advanced head and neck cancer. A phase II-III trial. Ann Oncol. 2017;28:2206-12. DOI: 10.1093/annonc/mdx299.
[38] Patil VM, Noronha V, Joshi A, Agarwal J, Ghosh-Laskar S, Budrukkar A, et al. A randomized phase 3 trial comparing nimotuzumab plus cisplatin chemoradiotherapy versus cisplatin chemoradiotherapy alone in locally advanced head and neck cancer. Cancer. 2019;125:3184-97. DOI: 10.1002/cncr.32179.
[39] Gregoire V, Hamoir M, Chen C, Kane M, Kawecki A, Julka PK, et al. Gefitinib plus cisplatin and radiotherapy in previously untreated head and neck squamous cell carcinoma: a phase II, randomized, double-blind, placebo-controlled study. Radiother Oncol. 2011;100:62-9. DOI: 10.1016/j.radonc.2011.07.008.
[40] Saini SK, Srivastava S, Dixit AK. Gefitinib with concurrent chemoradiation in locally advanced head neck cancer. Gaceta Mexicana de Oncologia. 2018;17:192-7.
[41] Yao M, Woods C, Lavertu P, Fu P, Gibson M, Rezaee R, et al. Phase II study of erlotinib and docetaxel with concurrent intensity-modulated radiotherapy in locally advanced head and neck squamous cell carcinoma. Head Neck. 2016;38 Suppl 1:E1770-6. DOI: 10.1002/hed.24313.
[42] Margalit DN, Haddad RI, Tishler RB, Chau NG, Schoenfeld JD, Bakst RL, et al. A Phase 1 Study of Afatinib in Combination with Postoperative Radiation Therapy with and Without Weekly Docetaxel in Intermediate- and High-Risk Patients with Resected Squamous Cell Carcinoma of the Head and Neck. International Journal of Radiation Oncology, Biology, Physics. 2019;105:132-9.
[43] Bradley JD, Hu C, Komaki RR, Masters GA, Blumenschein GR, Schild SE, et al. Long-Term Results of NRG Oncology RTOG 0617: Standard- Versus High-Dose Chemoradiotherapy With or Without Cetuximab for Unresectable Stage III Non-Small-Cell Lung Cancer. Journal of Clinical Oncology. 2020;38:706-14.
[44] Chun SG, Hu C, Choy H, Komaki RU, Timmerman RD, Schild SE, et al. Impact of Intensity-Modulated Radiation Therapy Technique for Locally Advanced Non-Small-Cell Lung Cancer: A

Secondary Analysis of the NRG Oncology RTOG 0617 Randomized Clinical Trial. J Clin Oncol. 2017;35:56-62. DOI: 10.1200/JCO.2016.69.1378.
[45] Bradley JD, Paulus R, Komaki R, Masters G, Blumenschein G, Schild S, et al. Standard-dose versus high-dose conformal radiotherapy with concurrent and consolidation carboplatin plus paclitaxel with or without cetuximab for patients with stage IIIA or IIIB non-small-cell lung cancer (RTOG 0617): a randomised, two-by-two factorial phase 3 study. Lancet Oncology. 2015;16:187-99.
[46] Crosby T, Hurt CN, Falk S, Gollins S, Mukherjee S, Staffurth J, et al. Chemoradiotherapy with or without cetuximab in patients with oesophageal cancer (SCOPE1): a multicentre, phase 2/3 randomised trial. Lancet Oncology. 2013;14:627-37.
[47] Crosby T, Hurt CN, Falk S, Gollins S, Staffurth J, Ray R, et al. Long-term results and recurrence patterns from SCOPE-1: a phase II/III randomised trial of definitive chemoradiotherapy +/- cetuximab in oesophageal cancer. British Journal of Cancer. 2017;116:709-16.
[48] Ruhstaller T, Thuss-Patience P, Hayoz S, Schacher S, Knorrenschild JR, Schnider A, et al. Neoadjuvant chemotherapy followed by chemoradiation and surgery with and without cetuximab in patients with resectable esophageal cancer: a randomized, open-label, phase III trial (SAKK 75/08). Annals of Oncology. 2018;29:1386-93.
[49] Xing L, Wu G, Wang L, Li J, Wang J, Yuan Z, et al. Erlotinib vs etoposide/cisplatin with radiotherapy in unresectable stage III epidermal growth factor receptor mutation-positive non-small-cell lung cancer: A multicenter, randomized, open-label, phase 2 trial. International Journal of Radiation Oncology, Biology, Physics. 2020;18:18.
[50] Liu T, He Z, Dang J, Li G. Comparative efficacy and safety for different chemotherapy regimens used concurrently with thoracic radiation for locally advanced non-small cell lung cancer: a systematic review and network meta-analysis. Radiation Oncology. 2019;14:55.
[51] Choi HJ, Sohn JH, Lee CG, Shim HS, Lee IJ, Yang WI, et al. A phase I study of nimotuzumab in combination with radiotherapy in stages IIB-IV non-small cell lung cancer unsuitable for radical therapy: Korean results. Lung Cancer. 2011;71:55-9. DOI: 10.1016/j.lungcan.2010.04.010.
[52] Xie C, Jing Z, Luo H, Jiang W, Ma L, Hu W, et al. Chemoradiotherapy with extended nodal irradiation and/or erlotinib in locally advanced oesophageal squamous cell cancer: long-term update of a randomised phase 3 trial. British Journal of Cancer. 2020;123:1616-24.
[53] Wu SX, Wang LH, Luo HL, Xie CY, Zhang XB, Hu W, et al. Randomised phase III trial of concurrent chemoradiotherapy with extended nodal irradiation and erlotinib in patients with inoperable oesophageal squamous cell cancer. Eur J Cancer. 2018;93:99-107. DOI: 10.1016/j.ejca.2018.01.085.
[54] Swaminath A, Wright JR, Tsakiridis TK, Ung YC, Pond GR, Sur R, et al. A Phase II Trial of Erlotinib and Concurrent Palliative Thoracic Radiation for Patients With Non-Small-Cell Lung Cancer. Clin Lung Cancer. 2016;17:142-9. DOI: 10.1016/j.cllc.2015.09.008.
[55] Komaki R, Allen PK, Wei X, Blumenschein GR, Tang X, Lee JJ, et al. Adding Erlotinib to Chemoradiation Improves Overall Survival but Not Progression-Free Survival in Stage III Non-Small Cell Lung Cancer. International Journal of Radiation Oncology, Biology, Physics. 2015;92:317-24.
[56] Chan OSH, Lam KC, Li JYC, Choi FPT, Wong CYH, Chang ATY, et al. ATOM: A phase II study to assess efficacy of preemptive local ablative therapy to residual oligometastases of NSCLC after EGFR TKI. Lung Cancer. 2020;142:41-6. DOI: 10.1016/j.lungcan.2020.02.002.
[57] Marquez-Medina D, Chachoua A, Martin-Marco A, Desai AM, Garcia-Reglero V, Salud-Salvia A, et al. Continued erlotinib maintenance and salvage radiation for solitary areas of disease progression: a useful strategy in selected non-small cell lung cancers? Clin Transl Oncol. 2013;15:959-64. DOI: 10.1007/s12094-013-1035-z.
[58] Syahruddin E, Huswatun AL, Prabowo A, Zaini J, Nurwidya F, Hudoyo A, et al. Efficacy of gefitinib and radiotherapy combination in Indonesian patients with lung adenocarcinoma. Rom J Intern Med. 2018;56:173-81. DOI: 10.2478/rjim-2018-0011.

[59] Jia W, Guo H, Jing W, Jing X, Li J, Wang M, et al. An especially high rate of radiation pneumonitis observed in patients treated with thoracic radiotherapy and simultaneous osimertinib. Radiotherapy & Oncology. 2020;152:96-100.
[60] Hammel P, Huguet F, van Laethem JL, Goldstein D, Glimelius B, Artru P, et al. Effect of Chemoradiotherapy vs Chemotherapy on Survival in Patients With Locally Advanced Pancreatic Cancer Controlled After 4 Months of Gemcitabine With or Without Erlotinib: The LAP07 Randomized Clinical Trial. JAMA. 2016;315:1844-53.
[61] Hammel P. Gemcitabine With or Without Capecitabine and/or Radiation Therapy or Gemcitabine With or Without Erlotinib in Treating Patients With Locally Advanced Pancreatic Cancer That Cannot Be Removed by Surgery. https://clinicaltrials.gov/ct2/show/NCT00634725. Updated December 11, 2015. Accessed March 21, 2023.
[62] Berlin JD, Feng Y, Catalano P, Abbruzzese JL, Philip PA, McWilliams RR, et al. An Intergroup Randomized Phase II Study of Bevacizumab or Cetuximab in Combination with Gemcitabine and in Combination with Chemoradiation in Patients with Resected Pancreatic Carcinoma: A Trial of the ECOG-ACRIN Cancer Research Group (E2204). Oncology. 2018;94:39-46. DOI: 10.1159/000480295.
[63] Maurel J, Sanchez-Cabus S, Laquente B, Gaba L, Visa L, Fabregat J, et al. Outcomes after neoadjuvant treatment with gemcitabine and erlotinib followed by gemcitabine-erlotinib and radiotherapy for resectable pancreatic cancer (GEMCAD 10-03 trial). Cancer Chemother Pharmacol. 2018;82:935-43. DOI: 10.1007/s00280-018-3682-9.
[64] Robertson JM, Margolis J, Jury RP, Balaraman S, Cotant MB, Ballouz S, et al. Phase I study of conformal radiotherapy and concurrent full-dose gemcitabine with erlotinib for unresected pancreatic cancer. Int J Radiat Oncol Biol Phys. 2012;82:e187-92. DOI: 10.1016/j.ijrobp.2010.08.050.
[65] Bertolini F, Chiara S, Bengala C, Antognoni P, Dealis C, Zironi S, et al. Neoadjuvant treatment with single-agent cetuximab followed by 5-FU, cetuximab, and pelvic radiotherapy: a phase II study in locally advanced rectal cancer. Int J Radiat Oncol Biol Phys. 2009;73:466-72. DOI: 10.1016/j.ijrobp.2008.04.065.
[66] Valentini V, De Paoli A, Gambacorta MA, Mantini G, Ratto C, Vecchio FM, et al. Infusional 5-fluorouracil and ZD1839 (Gefitinib-Iressa) in combination with preoperative radiotherapy in patients with locally advanced rectal cancer: a phase I and II Trial (1839IL/0092). Int J Radiat Oncol Biol Phys. 2008;72:644-9. DOI: 10.1016/j.ijrobp.2008.01.046.
[67] Gambacorta MA, De Paoli A, Lupattelli M, Chiloiro G, Solazzo AP, Barbaro B, et al. Phase I and II trial on infusional 5-fluorouracil and gefitinib in combination with preoperative radiotherapy in rectal cancer: 10-years median follow-up. Clin Transl Radiat Oncol. 2018;10:23-8. DOI: 10.1016/j.ctro.2018.02.003.
[68] Leichman CG, McDonough SL, Smalley SR, Billingsley KG, Lenz HJ, Beldner MA, et al. Cetuximab Combined With Induction Oxaliplatin and Capecitabine, Followed by Neoadjuvant Chemoradiation for Locally Advanced Rectal Cancer: SWOG 0713. Clin Colorectal Cancer. 2018;17:e121-e5. DOI: 10.1016/j.clcc.2017.10.008.
[69] Dewdney A, Cunningham D, Tabernero J, Capdevila J, Glimelius B, Cervantes A, et al. Multicenter randomized phase II clinical trial comparing neoadjuvant oxaliplatin, capecitabine, and preoperative radiotherapy with or without cetuximab followed by total mesorectal excision in patients with high-risk rectal cancer (EXPERT-C). Journal of Clinical Oncology. 2012;30:1620-7.

ALK (anaplastic lymphoma kinase) inhibitors

1. Common ALK inhibitors

Name	Drug type	$T_{1/2}$	$T_{1/2\ el}$ x 5	Weblinks
**Alectinib**<br>*Alecensa*	TKI	33 h<br>(31 h for active metabolite)	7 d	PubChem<br>FDA
**Brigatinib**<br>*Alunbrig*	TKI	25 h	5 d	PubChem<br>FDA
**Ceritinib**<br>*Zykadia*	TKI	41 h	9 d	PubChem<br>FDA
**Crizotinib**<br>*Xalkori*	TKI	42 h	9 d	PubChem<br>FDA
**Lorlatinib**<br>*Lorbrena*<br>*Lorviqua*	TKI	24 h	5 d	PubChem<br>FDA

TKI = tyrosine kinase inhibitor; $T_{1/2}$ = plasma half-life; h = hours; d = days.

2. Biological & pharmacological data

2.1 Pathway & drug mechanism

ALK signaling influences several major oncogenic pathways, including Ras, phospholipase C-γ (PLC γ), signal transducer and activator of transcription 3 (STAT3) and phosphatidylinositol 3-kinase (PI3K), which contribute to cell survival and proliferation [1, 2]. Increased ALK activation is primarily caused by chromosomal translocations or inversions, leading to fusion proteins (e.g. EML4-ALK in non-small cell lung cancer) that lead to ligand-independent activation of the ALK protein kinase domains [2, 3]. Also mutations or ALK amplification can play a role in certain cancer types [2, 3].

Inhibition of ALK (all), IGF1R (Brigatinib), MET(Crizotinib), RET (Alectinib), ROS1 (Brigatinib, Crizotinib and Lorlatinib) receptor tyrosine kinases downregulates Ras, PI3K and STAT3 signaling [4], leading to inhibition of growth/proliferation, less cells in the radioresistant S-phase [5] and inhibition of anti-apoptotic signals, possibly leading to radiosensitization of (mostly rapidly proliferating) normal tissue and tumor cells. So in general, reduced cell survival and repopulation by ALK inhibitors may lead to an increased risk of radiotherapy toxicity, whereas inhibition of growth/proliferation could lead to a decreased amount of cells in the radiosensitive M-phase [5], leading to more radioresistance.

2.2 Organ-specific availability

While crizotinib has a poor blood-brain barrier penetrance, alectinib, brigatinib, ceritinib and lorlatinib have been shown to improve intracranial control and thus to cross the blood-brain barrier [6, 7].

3. Literature review

General summary

Information is scarce and consists of case-reports and small retrospective series. Most data are available about radiotherapy to the CNS. Based on available biological/pharmacological data it can be hypothesized that survival and repopulation of (particularly fast dividing) normal tissues could be reduced by ALK inhibitors after radiotherapy, thus increasing the toxicity of radiotherapy. Most interactions could be expected in days to weeks after radiotherapy (acute phase). However, evidence is scarce and primarily

present for crizotinib. A case of severe (G4) esophagitis has been reported with crizotinib and 10 x 3 Gy on the cervical spine. Gammaknife radiotherapy for brain metastases seemed safe during crizotinib. However, it should be noted that crizotinib is the only drug reported to not cross the blood-brain barrier, so other ALK inhibitors might give more side effects when combined with CNS-directed radiotherapy.

Skin

No severe skin toxicity is reported, but the available safety data are very limited.

A **case report** from Brunac et al. (2020) shows G2 dermatitis and local temporary alopecia in a patient that received 30 x 2 Gy to a craniovertebral mass and started crizotinib 5 days after radiotherapy [8].

Brain

In general, no high rates of ≥G3 toxicity are reported. Most data concern crizotinib use during radiotherapy, which has a limited blood brain barrier penetration [6, 9]. These results should therefore not be extrapolated to newer TKIs with a higher brain penetration rate. Furthermore, some relatively larger studies combine data from patients using ALK and EGFR inhibitors [10-14], which complicates interpretation of the ALK-specific results.

One **retrospective study** in 29 patients concludes that gammaknife radiotherapy can be safely administered during crizotinib use [15]. Another **retrospective study** describes a higher rate of radionecrosis after SRS (stereotactic radiosurgery) (18% vs. 4% at 12 months, multivariable analysis, $p<0.001$) in ALK+ patients, but no significant association with concurrent ALK inhibitor use within 30 days of SRS, which took place in 15 patients [12]. **Nakashima et al.** show G3 otitis media in both patients who received whole brain radiotherapy (WBRT) with concurrent ALK inhibition (crizotinib and alectinib) and tinnitus complaints in one patient 33 months after WBRT [13]. One small **retrospective study** of 24 patients with mainly cerebral, bone and lung metastases describes G3 fatigue within 6 months after WBRT in 2/6 patients, but no unexpected side effects. However, they do not describe whether these patients received either crizotinib (ALK inhibitor) or erlotinib (EGFR inhibitor) [14]. **Borghetti et al.** do not report unexpected toxicities when SRS and non-SRS radiotherapy are combined with ALK (primarily crizotinib) or EGFR inhibitors within 30 days [10, 11]. Furthermore, the **case reports** do not clearly show extra toxicity of concurrent or sequential ALK TKI use [16-20].

Although it is not the scope of this review, it is noteworthy to mention that three **case-reports** describe severe radionecrosis after administration of alectinib or lorlatinib within 4 months up to 7 years after stereotactic radiotherapy [21-23]. It is unknown whether these findings are incidental or if ALK inhibitors may lead to an interaction with late normal tissue reactions to radiotherapy.

Head and neck

The available safety data are very limited.

We only identified two **case reports** regarding ALK inhibitors combined with head and neck radiotherapy. Brunac et al. (2020) report that 30 x 2 Gy to a craniovertebral mass and start of crizotinib 5 days after radiotherapy was well-tolerated (G2 radiomucositis) in a patient [8]. A case report from Zimmermann et al. (2017) shows G4 ulceration of the hypopharynx and upper esophagus with crizotinib and a Dmax <30 Gy to these OARs [24]. Furthermore, the risk of ototoxicity should be taken into account, which is described in the ‘Brain’ section [13].

Thorax

The available safety data are very limited.

Borghetti et al. report no toxicities for the combination with lung SRT. However, due to different timing schedules and the combination of data of both ALK and EGFR inhibitors, no specific conclusions can be drawn based on this study [10]. Two other **retrospective studies** also include thorax radiotherapy combined with crizotinib (and erlotinib in the study of Weickhardt et al.), without clearly increased toxicity, although the medication was paused during radiotherapy and description of the treatment details is limited

[14, 25]. The earlier mentioned **case report** from Zimmermann et al. (2017) shows G4 ulceration of the hypopharynx and upper esophagus with crizotinib and a Dmax <30 Gy to these OARs [24].

Abdomen/pelvis

The available safety data are very limited.

The studies of **Gan et al.** and **Weickhardt et al**. also include abdominal radiotherapy (drug paused during RT) and they do not describe increased toxicity [14, 25]. In the study of Weickhardt et al., one patient received liver SBRT combined with crizotinib or the EGFR inhibitor erlotinib, without toxicity [14].

Musculoskeletal

The available safety data are very limited, but do not indicate increased toxicity risks.

In a study of **Borghetti et al.** (2019), 28 patients (3 patients SRT, 25 patients hypofractionated RT) received radiotherapy of a bone structure combined with ALK or EGFR inhibitors (within 30 days of RT). In the hypofractionated group, G1 pain was seen in 28%, G2 pain in 40% and G3 pain in 4%. No pain was observed in the SRT group. In general, they conclude that there is no increased toxicity [10]. The same conclusions are drawn in another study of **Borghetti et al.** (drug within 30 days of RT) and a study from **Weickhardt et al.** (drug paused during RT) [11, 14].

Bibliography

[1] Chiarle R, Voena C, Ambrogio C, Piva R, Inghirami G. The anaplastic lymphoma kinase in the pathogenesis of cancer. Nat Rev Cancer. 2008;8:11-23. DOI: 10.1038/nrc2291.
[2] Roskoski R, Jr. Anaplastic lymphoma kinase (ALK): structure, oncogenic activation, and pharmacological inhibition. Pharmacol Res. 2013;68:68-94. DOI: 10.1016/j.phrs.2012.11.007.
[3] Hallberg B, Palmer RH. The role of the ALK receptor in cancer biology. Ann Oncol. 2016;27 Suppl 3:iii4-iii15. DOI: 10.1093/annonc/mdw301.
[4] Cooper WA, Lam DC, O'Toole SA, Minna JD. Molecular biology of lung cancer. J Thorac Dis. 2013;5 Suppl 5:S479-90. DOI: 10.3978/j.issn.2072-1439.2013.08.03.
[5] Pawlik TM, Keyomarsi K. Role of cell cycle in mediating sensitivity to radiotherapy. Int J Radiat Oncol Biol Phys. 2004;59:928-42. DOI: 10.1016/j.ijrobp.2004.03.005.
[6] Petrelli F, Lazzari C, Ardito R, Borgonovo K, Bulotta A, Conti B, et al. Efficacy of ALK inhibitors on NSCLC brain metastases: A systematic review and pooled analysis of 21 studies. PLoS One. 2018;13:e0201425. DOI: 10.1371/journal.pone.0201425.
[7] Bauer TM, Shaw AT, Johnson ML, Navarro A, Gainor JF, Thurm H, et al. Brain Penetration of Lorlatinib: Cumulative Incidences of CNS and Non-CNS Progression with Lorlatinib in Patients with Previously Treated ALK-Positive Non-Small-Cell Lung Cancer. Target Oncol. 2020;15:55-65. DOI: 10.1007/s11523-020-00702-4.
[8] Brunac AC, Laprie A, Castex MP, Laurent C, Le Loarer F, Karanian M, et al. The combination of radiotherapy and ALK inhibitors is effective in the treatment of intraosseous rhabdomyosarcoma with FUS-TFCP2 fusion transcript. Pediatric Blood and Cancer. 2020;67.
[9] Costa DB, Kobayashi S, Pandya SS, Yeo WL, Shen Z, Tan W, et al. CSF concentration of the anaplastic lymphoma kinase inhibitor crizotinib. J Clin Oncol. 2011;29:e443-5. DOI: 10.1200/JCO.2010.34.1313.
[10] Borghetti P, Bonu ML, Giubbolini R, Levra NG, Mazzola R, Perna M, et al. Concomitant radiotherapy and TKI in metastatic EGFR- or ALK-mutated non-small cell lung cancer: a multicentric analysis on behalf of AIRO lung cancer study group. Radiol Med. 2019;124:662-70. DOI: 10.1007/s11547-019-00999-w.
[11] Borghetti P, Bonu ML, Roca E, Pedretti S, Salah E, Baiguini A, et al. Radiotherapy and Tyrosine Kinase Inhibitors in Stage IV Non-small Cell Lung Cancer: Real-life Experience. In Vivo. 2018;32:159-64. DOI: 10.21873/invivo.11219.
[12] Miller JA, Kotecha R, Ahluwalia MS, Mohammadi AM, Suh JH, Barnett GH, et al. The impact of tumor biology on survival and response to radiation therapy among patients with non-small cell lung cancer brain metastases. Practical Radiation Oncology. 2017;7:e263-e73. DOI: 10.1016/j.prro.2017.01.001.
[13] Nakashima T, Nonoshita T, Hirata H, Inoue K, Nagashima A, Yoshitake T, et al. Adverse Events of Concurrent Radiotherapy and ALK Inhibitors for Brain Metastases of ALK-Rearranged Lung Adenocarcinoma. In Vivo. 2020;34:247-53. DOI: 10.21873/invivo.11767.
[14] Weickhardt AJ, Scheier B, Burke JM, Gan G, Lu X, Bunn PA, Jr., et al. Local ablative therapy of oligoprogressive disease prolongs disease control by tyrosine kinase inhibitors in oncogene-addicted non-small-cell lung cancer. J Thorac Oncol. 2012;7:1807-14. DOI: 10.1097/JTO.0b013e3182745948.
[15] Choi JW, Kong DS, Seol HJ, Nam DH, Yoo KH, Sun JM, et al. Outcomes of Gamma Knife Radiosurgery in Combination with Crizotinib for Patients with Brain Metastasis from Non-Small Cell Lung Cancer. World Neurosurg. 2016;95:399-405. DOI: 10.1016/j.wneu.2016.08.046.
[16] Dudnik E, Siegal T, Zach L, Allen AM, Flex D, Yust-Katz S, et al. Durable brain response with pulse-dose crizotinib and ceritinib in ALK-positive non-small cell lung cancer compared with brain radiotherapy. J Clin Neurosci. 2016;26:46-9. DOI: 10.1016/j.jocn.2015.05.068.
[17] Onesti CE, Iacono D, Angelini S, Mazzotta M, Giusti R, Lauro S, et al. Four lines of anaplastic lymphoma kinase inhibitors and brain radiotherapy in a long-surviving non-small-cell lung cancer

anaplastic lymphoma kinase-positive patient with leptomeningeal carcinomatosis. Anticancer Drugs. 2019;30:201-4. DOI: 10.1097/CAD.0000000000000699.
[18] Pinto IG, Lee M, Graziano S, Lacombe M, Gajra A. Concurrent crizotinib and whole-brain radiation for brain metastases in ALK-positive lung adenocarcinoma. Lung Cancer Management. 2014;3:369-71.
[19] Tanigawa K, Mizuno K, Kamenohara Y, Unoki T, Misono S, Inoue H. Effect of bevacizumab on brain radiation necrosis in anaplastic lymphoma kinase-positive lung cancer. Respirol Case Rep. 2019;7:e00454. DOI: 10.1002/rcr2.454.
[20] Urbanska EM, Santoni-Rugiu E, Melchior LC, Carlsen JF, Sorensen JB. Intracranial Response of ALK Non-Small-cell Lung Cancer to Second-line Dose-escalated Brigatinib After Alectinib Discontinuation Due to Drug-induced Hepatitis and Relapse After Whole Brain Radiotherapy Followed by Stereotactic Radiosurgery. Clinical Lung Cancer. 2020;4:04. DOI: 10.1016/j.cllc.2020.04.012.
[21] Ou SH, Klempner SJ, Azada MC, Rausei-Mills V, Duma C. Radiation necrosis presenting as pseudoprogression (PsP) during alectinib treatment of previously radiated brain metastases in ALK-positive NSCLC: Implications for disease assessment and management. Lung Cancer. 2015;88:355-9. DOI: 10.1016/j.lungcan.2015.03.022.
[22] Ou SH, Weitz M, Jalas JR, Kelly DF, Wong V, Azada MC, et al. Alectinib induced CNS radiation necrosis in an ALK+NSCLC patient with a remote (7 years) history of brain radiation. Lung Cancer. 2016;96:15-8. DOI: 10.1016/j.lungcan.2016.03.008.
[23] Zhu VW, Nagasaka M, Kubota T, Raval K, Robinette N, Armas O, et al. Symptomatic CNS Radiation Necrosis Requiring Neurosurgical Resection During Treatment with Lorlatinib in ALK-Rearranged NSCLC: A Report of Two Cases. Lung Cancer (Auckl). 2020;11:13-8. DOI: 10.2147/LCTT.S224991.
[24] Zimmermann MH, Beckmann G, Jung P, Flentje M. Hypopharyngeal and upper esophageal ulceration after cervical spine radiotherapy concurrent with crizotinib. Strahlenther Onkol. 2017;193:589-92. DOI: 10.1007/s00066-017-1135-8.
[25] Gan GN, Weickhardt AJ, Scheier B, Doebele RC, Gaspar LE, Kavanagh BD, et al. Stereotactic radiation therapy can safely and durably control sites of extra-central nervous system oligoprogressive disease in anaplastic lymphoma kinase-positive lung cancer patients receiving crizotinib. International Journal of Radiation Oncology, Biology, Physics. 2014;88:892-8.

BRAF (B-rapidly accelerated fibrosarcoma) and MEK (Mitogen-activated protein kinase kinase) inhibitors

1. Common BRAF and MEK inhibitors

BRAF

Name	Drug type	$T_{1/2}$	$T_{1/2}$ x 5	Weblinks
**Dabrafenib** *Tafinlar*	TKI	8-22h (different metabolites)	1.7-4.6d	PubChem FDA
**Encorafenib** *Braftovi*	TKI	3.5h	17.5h	PubChem FDA
**Vemurafenib** *Zelboraf*	TKI	57h (30-120h)	11.9d	PubChem FDA

MEK

Name	Drug type	$T_{1/2}$	$T_{1/2}$ x 5	Weblinks
**Trametinib** *Mekinist*	TKI	3.9-4.8d	19.5-24d	PubChem FDA
**Cobimetinib** *Cotellic*	TKI	44h (23-70h)	9.2d	PubChem FDA
**Binimetinib** *Mektovi*	TKI	3.5h	17.5h	PubChem FDA

TKI = tyrosine kinase inhibitor; $T_{1/2}$ = plasma half-life; h = hours; d = days.

2. Biological & pharmacological data

2.1 Pathway & drug mechanism

BRAF is part of the RAS-RAF-MEK-ERK signaling pathway. In BRAF-mutated tumors (most frequent mutation: BRAFV600E) activation of BRAF, independently of upstream signaling, can cause cell proliferation [1]. Inhibition of BRAF protein kinases downregulates RAF signaling, leading to inhibition of growth/proliferation in BRAF-mutated cells [1]. Inhibition of BRAF can also lead to paradoxical MAPK signaling in RAS-mutant and RAS/RAF wild-type cells, leading to fast division of these cells [2, 3]. Additionally, increased VEGF production has been described [4]. Faster proliferation of keratinocytes can lead to more cells in M-phase and consequently a heightened radiosensitivity. Alternatively, inhibition of proliferation could lead to less repopulation after radiotherapy.

MEK inhibitors (MEKi) inhibit activation of MEK, downstream of RAF. The combination with a BRAF inhibitor (BRAFi) can increase progression-free and overall survival in patients with BRAF V600-mutated melanoma. Additionally, this combination probably reduces the risk of paradoxical MAPK signaling-induced hyperkeratosis and cutaneous squamous-cell carcinoma [5, 6].

3. Literature review

General summary

The combination of RT with BRAFi ± MEKi increases the risk of skin toxicity, particularly radiation dermatitis. Stereotactic RT may be combined without increased skin toxicity when the dose to the skin is low. Toxicity after brain RT appears limited, but some studies report increased neurological toxicity. Data on RT to other sites is even more limited. Concurrent RT is not an absolute contra-indication in patients using BRAFi ± MEKi. However, caution is needed, due to the increased skin toxicity risk, mixed results with regard to brain

RT and limited toxicity data regarding other RT sites. Reducing skin dose and increasing the time interval between RT and targeted therapy may reduce this risk. Data regarding MEK-only inhibition combined with RT is even more scarce. In the combination studies with BRAFi, the addition of MEKi does not appear to increase RT toxicity.

Skin

The literature data clearly indicate an increased risk of skin toxicity when radiotherapy is given concurrently or in close proximity to BRAFi ± MEKi, particularly in combination with vemurafenib. Stereotactic RT may be combined without increased skin toxicity when the dose to the skin is low. Reducing skin dose and temporary drug interruption probably reduces the risk of an interaction, but does not exclude this possibility. MEKi-specific data are scarce, but no clear additional skin toxicity is observed compared to BRAFi alone.

Numerous small **retrospective (case) studies** report increased skin toxicity (primarily G2-3); in particular radiation dermatitis, hyperkeratosis and cutis verticis gyrata [7-25]. Increased skin reactions in the irradiated field are seen, even when vemurafenib is (re)started up to one week after RT [8, 9, 12, 14, 23]. Delayed skin reactions after concurrent therapy [18, 25] and radiation recall dermatitis [9, 11] are reported as well. These skin toxicities are seen in combination with both low-dose and high-dose RT. Conversely, a small **retrospective study** mentioned no increased toxicity is to be expected from BRAF/MEKi and radiotherapy [26].

Hecht et al. published two large **retrospective studies** (n=161 and n=155, possibly overlapping patients) regarding the combination of BRAFi (few with MEKi) with RT [27, 28]. They report that G≥2 radiation dermatitis risk was 44% after RT with BRAFi and 8% without BRAFi ($p<0.001$) [27]. There was a trend towards more G≥2 radiation dermatitis with vemurafenib (36-40%) than with dabrafenib (21-26%) [27, 28]. When BRAFi were discontinued (median 4 days) before radiotherapy, G≥2 dermatitis was observed in 14% (vemurafenib) and 0% (dabrafenib) [28]. Follicular cystic proliferation (including 1 case with cutis verticis gyrata) only occurred in the concurrent vemurafenib group [27, 28]. As a measure for individual radiosensitivity, peripheral blood lymphocytes showed higher radiosensitivity in patients using vemurafenib ($p=0.004$) and in patients who switched from vemurafenib to dabrafenib ($p=0.002$), but not for dabrafenib alone [27]. A higher skin toxicity rate was not observed in patients receiving stereotactic RT + BRAFi [27].

This finding was confirmed by **Kroeze et al.** (2021) who report no skin toxicities in a retrospective analysis including 43 patients receiving BRAFi ± MEKi combined with stereotactic radiotherapy (within 30 days) [29] and several smaller **retrospective (case) studies** on stereotactic brain RT in close proximity to or concurrent with BRAFi ± MEKi [19, 30-35]. The absence of increased skin toxicity in patients receiving stereotactic radiotherapy indicates a dose-response relationship, which is also illustrated in a **patient** receiving different skin doses with concomitant dabrafenib [7].

Very few clinical data are available regarding MEK inhibition alone. Two **phase I trials** add MEK inhibitor trametinib or selumetinib to chemoradiotherapy for locally advanced rectal cancer [36, 37]. In both studies the most dominant skin toxicity was rash (location not defined), which is also a side effect of these MEK inhibitors alone [38, 39]. Trametinib was discontinued in one patient and held for the last 3 days of chemoradiation in two other patients due to rash [37].

Brain

A number of retrospective studies and case reports have been published with various methodologies, toxicity analyses and time intervals between BRAFi ± MEKi and RT. In many studies, BRAFi/MEKi are temporarily paused. Although some studies show higher neurological toxicity rates when BRAFi/MEKi are combined with RT (concurrently or within a certain time interval), several other studies do not report increased toxicity. Combined therapy is therefore not an absolute contra-indication with regard to neurological toxicity, but due to the low quality and heterogeneity of the data, increased neurological toxicity cannot be ruled out. We found no studies investigating brain RT combined with a MEKi without a BRAFi.

In the study of **Hecht et al.** (2018), in both the concomitant vemurafenib (24 WBRT, 14 brain SRT) and dabrafenib (7 WBRT, 9 brain SRT) group, one patient was identified with a hemorrhagic brain metastasis. This toxicity did not occur in the (smaller) interrupted groups (vemurafenib 23 WBRT, 9 brain SRT;

dabrafenib 5 WBRT, 7 brain SRT) [28]. One case with a hemorrhagic brain metastasis was mentioned in **their earlier study** [27]. However, melanoma brain metastasis hemorrhage also regularly occurs without treatment [40, 41]. **Kroeze et al.** (2021) report more all-grade early (p=0.014) and late (p=0.009) toxicity when BRAFi/MEKi are continued during (multi-site) SRT, compared to interrupted SRT, but G≥3 toxicity is not increased. All toxicity in the BRAFi/MEKi group was CNS-related, but the toxicity details are not specified per treatment group [29]. Several small **retrospective studies** with often <30 patients receiving brain RT combined with BRAFi ± MEKi at different time intervals and with different RT techniques, do not show increased rates of radionecrosis, brain metastasis hemorrhage or other (high-grade) toxicities [26, 30-33, 42-45].

However, **Ly et al.** (2015) show a higher risk of brain metastasis hemorrhage in 17 patients treated with SRS and BRAFi with a median washout period of 7 days. The 1-year freedom from hemorrhage rate was 39.3% in patients with BRAFi and 77.0% in patients without BRAFi (p=0.0003). The 1-year local control rate with BRAFi was better (85.0% vs. 51.5%, p=0.0077) [46]. **Patel et al.** (2016) show a significantly higher risk of radiographic (HR=3.38, p=0.011) and symptomatic (HR=6.10, p≤0.001) radiation necrosis after SRS in patients treated with BRAFi vs. no BRAFi, although the majority (10/15) in the BRAFi group started BRAFi after SRS (median interval between SRS and BRAFi was 40 days) [47].

Other even **smaller studies and case reports** concerning brain SRT or WBRT show mixed results: some with severe neurological toxicity [16, 48, 49] and others with acceptable or no neurological toxicity [17, 19, 34, 50].

Head and neck

*Apart from two **case reports** concerning increased skin toxicity [12, 23], we identified no studies concerning head and neck RT combined with BRAFi/MEKi. **Hecht et al.** (2015 and 2018) report hearing disorder in 0-7%, which might also be related to e.g. WBRT [27, 28].*

Thorax

*Very limited data are available regarding thoracic RT combined with BRAFi/MEKi. Two **case reports** show increased toxicity, but the other studies do not clearly indicate increased non-skin-related thoracic RT toxicity. Nevertheless, caution is needed when combining these therapies due to the low amount of toxicity data.*

Hecht et al. (2015) report dysphagia in 2% of all 86 treatments combined with a BRAFi. The RT targets in this study vary, but contain 3 mediastinal metastases [27]. In a **more recent study** of Hecht et al. (2018), dysphagia (n=2), pneumonitis (n=1) and cardiac insufficiency (n=1) are described in a group receiving concomitant vemurafenib (n=59), but again, it is unknown whether these toxicities were related to mediastinal RT (n=4) [28]. **Merten et al.** (2014) report a patient with G3 esophagitis (mean oesophagus dose 31 Gy) after RT to thoracic vertebral bone metastases [15]. Another **case report** describes severe spinal cord myelopathy in a 14-year old male after 2 x 8.5 Gy to a mediastinal node, combined with dabrafenib. Additionally, the patient developed a symptomatic radiation pneumonitis [51]. Several other **small studies** include thoracic RT, but focus on skin toxicity [7, 9, 10, 17, 21, 23].

Abdomen/pelvis

Although some increased toxicity is reported, very limited data are available regarding abdominal/pelvic RT combined with BRAFi/MEKi. Caution is needed when combining these therapies.

Two **case reports** describe the combination of vemurafenib and RT (1 x 10 Gy and 10 x 3 Gy) to lumbar vertebrae without adverse effects, apart from G3 dermatitis (10 x 3 Gy) [10, 35]. Another **case report** describes G5 hepatic toxicity (hemorrhagic liver cysts, venous thrombi and liver necrosis) after 5 x 4 Gy to T1-T7 and T10-L1 with a posterior-anterior beam [8]. One **patient** developed G3 anorectitis, diarrhea, anorexia, weight loss and severe pain after 10 x 3 Gy to the posterior pelvis + 10 x 2.5 Gy to a rectal mass [16]. Some other **case studies** with abdominal/pelvic RT only report skin toxicity [13, 17, 21, 23].

Two **phase I trials** add a MEK inhibitor trametinib or selumetinib to chemoradiotherapy for locally advanced rectal cancer [36, 37]. Trametinib (2 mg) combined with 28 x 1.8 Gy and 5-FU chemoradiotherapy was well-

tolerated. One dose-limiting toxicity (DLT) occurred (diarrhea), which was primarily attributed to chemoradiotherapy [37]. Addition of selumetinib was not tolerated well. In the first cohort (50 mg BID),G3 diarrhea (n=1) and G3 fatigue (n=1) were DLTs, in the de-escalation cohort G3 diarrhea (n=2) was a DLT [36].

Musculoskeletal
No increased musculoskeletal-specific toxicity is reported.

The **studies** of Hecht et al. (2015 and 2018) include patients with concurrent RT to bone metastases and soft tissue metastases, but no non-skin-related toxicities are specifically attributed to these RT targets [27, 28]. A **retrospective study** of Ziegler et al. (2020) includes 8 patients with conventional or stereotactic bone RT. They report G3 bone pain in one patient that received BRAFi+MEKi after RT, but it is unclear whether this was due to RT or due to tumor progression [26]. Other **case studies** concerning musculoskeletal RT primarily describe skin toxicity [13, 20, 21, 23-25].

References

[1] Ascierto PA, Kirkwood JM, Grob JJ, Simeone E, Grimaldi AM, Maio M, et al. The role of BRAF V600 mutation in melanoma. J Transl Med. 2012;10:85. DOI: 10.1186/1479-5876-10-85.

[2] Poulikakos PI, Zhang C, Bollag G, Shokat KM, Rosen N. RAF inhibitors transactivate RAF dimers and ERK signalling in cells with wild-type BRAF. Nature. 2010;464:427-30. DOI: 10.1038/nature08902.

[3] Hatzivassiliou G, Song K, Yen I, Brandhuber BJ, Anderson DJ, Alvarado R, et al. RAF inhibitors prime wild-type RAF to activate the MAPK pathway and enhance growth. Nature. 2010;464:431-5. DOI: 10.1038/nature08833.

[4] Wang T, Xiao M, Ge Y, Krepler C, Belser E, Lopez-Coral A, et al. BRAF Inhibition Stimulates Melanoma-Associated Macrophages to Drive Tumor Growth. Clin Cancer Res. 2015;21:1652-64. DOI: 10.1158/1078-0432.CCR-14-1554.

[5] Flaherty KT, Infante JR, Daud A, Gonzalez R, Kefford RF, Sosman J, et al. Combined BRAF and MEK inhibition in melanoma with BRAF V600 mutations. N Engl J Med. 2012;367:1694-703. DOI: 10.1056/NEJMoa1210093.

[6] Long GV, Stroyakovskiy D, Gogas H, Levchenko E, de Braud F, Larkin J, et al. Dabrafenib and trametinib versus dabrafenib and placebo for Val600 BRAF-mutant melanoma: a multicentre, double-blind, phase 3 randomised controlled trial. The Lancet. 2015;386:444-51. DOI: 10.1016/s0140-6736(15)60898-4.

[7] Alterio D, Marvaso G, Ferrari A, Alessandro O, Cocorocchio E, Ferrucci PF, et al. Combination of dabrafenib and radiotherapy: could skin toxicity be affected by different irradiation techniques? BJR Case Reports. 2016;2:20150493.

[8] Anker CJ, Ribas A, Grossmann AH, Chen X, Narra KK, Akerley W, et al. Severe liver and skin toxicity after radiation and vemurafenib in metastatic melanoma. J Clin Oncol. 2013;31:e283-7. DOI: 10.1200/JCO.2012.44.7755.

[9] Boussemart L, Boivin C, Claveau J, Tao YG, Tomasic G, Routier E, et al. Vemurafenib and radiosensitization. JAMA Dermatol. 2013;149:855-7. DOI: 10.1001/jamadermatol.2013.4200.

[10] Churilla TM, Chowdhry VK, Pan D, de la Roza G, Damron T, Lacombe MA. Radiation-induced dermatitis with vemurafenib therapy. Pract Radiat Oncol. 2013;3:e195-8. DOI: 10.1016/j.prro.2012.11.012.

[11] Harding JJ, Barker CA, Carvajal RD, Wolchok JD, Chapman PB, Lacouture ME. Cutis verticis gyrata in association with vemurafenib and whole-brain radiotherapy. J Clin Oncol. 2014;32:e54-6. DOI: 10.1200/JCO.2013.49.3528.

[12] Houriet C, Klass ND, Beltraminelli H, Borradori L, Oberholzer PA. Localized Epidermal Cysts as a Radiation Recall Phenomenon in a Melanoma Patient Treated with Radiotherapy and the BRAF Inhibitor Vemurafenib. Case Reports Dermatology. 2014;6:213-7.

[13] Kuo KY, Jiang W, Swetter SM, Kwong BY. Enhanced radiation dermatitis associated with concurrent palliative radiation and vemurafenib therapy. Cutis. 2016;98:E4-6.

[14] Lang N, Sterzing F, Enk AH, Hassel JC. Cutis verticis gyrata-like skin toxicity during treatment of melanoma patients with the BRAF inhibitor vemurafenib after whole-brain radiotherapy is a consequence of the development of multiple follicular cysts and milia. Strahlentherapie und Onkologie. 2014;190:1080-1.

[15] Merten R, Hecht M, Haderlein M, Distel L, Fietkau R, Heinzerling L, et al. Increased skin and mucosal toxicity in the combination of vemurafenib with radiation therapy. Strahlenther Onkol. 2014;190:1169-72. DOI: 10.1007/s00066-014-0698-x.

[16] Peuvrel L, Ruellan AL, Thillays F, Quereux G, Brocard A, Saint-Jean M, et al. Severe radiotherapy-induced extracutaneous toxicity under vemurafenib. Eur J Dermatol. 2013;23:879-81. DOI: 10.1684/ejd.2013.2193.

[17] Pulvirenti T, Hong A, Clements A, Forstner D, Suchowersky A, Guminski A, et al. Acute Radiation Skin Toxicity Associated With BRAF Inhibitors. J Clin Oncol. 2016;34:e17-20. DOI: 10.1200/JCO.2013.49.0565.
[18] Reigneau M, Granel-Brocard F, Geoffrois L, Bauman AS, Trechot P, Barbaud A, et al. Efflorescence of scalp cysts during vemurafenib treatment following brain radiation therapy: a radiation recall dermatitis? Eur J Dermatol. 2013;23:544-5. DOI: 10.1684/ejd.2013.2108.
[19] Rompoti N, Schilling B, Livingstone E, Griewank K, Hillen U, Sauerwein W, et al. Combination of BRAF Inhibitors and Brain Radiotherapy in Patients With Metastatic Melanoma Shows Minimal Acute Toxicity. Journal of Clinical Oncology. 2013;31:3844-5.
[20] Saco M, Mitchell C. Severe radiation dermatitis associated with concomitant vemurafenib therapy in a patient with metastatic melanoma. J Am Acad Dermatol. 2014;70:e135-6. DOI: 10.1016/j.jaad.2013.10.046.
[21] Satzger I, Degen A, Asper H, Kapp A, Hauschild A, Gutzmer R. Serious skin toxicity with the combination of BRAF inhibitors and radiotherapy. J Clin Oncol. 2013;31:e220-2. DOI: 10.1200/JCO.2012.44.4265.
[22] Schulze B, Meissner M, Wolter M, Rodel C, Weiss C. Unusual acute and delayed skin reactions during and after whole-brain radiotherapy in combination with the BRAF inhibitor vemurafenib. Two case reports. Strahlenther Onkol. 2014;190:229-32. DOI: 10.1007/s00066-013-0474-3.
[23] Strobel SB, Patzold S, Zimmer L, Jensen A, Enk A, Hassel JC. Radiosensitization by BRAF inhibitors. J Dtsch Dermatol Ges. 2017;15:703-8. DOI: 10.1111/ddg.12672.
[24] Ueki K, Kosaka Y, Kimino G, Imagumbai T, Takayama K, Kokubo M. Treatment of malignant melanoma with nivolumab and vemurafenib combined with hypofractionated radiation therapy. Int Cancer Conf J. 2016;5:214-8. DOI: 10.1007/s13691-016-0260-z.
[25] Yilmaz M, Celik U, Hascicek S. Radiation recall dermatitis with dabrafenib and trametinib: A case report. World J Clin Cases. 2020;8:522-6. DOI: 10.12998/wjcc.v8.i3.522.
[26] Ziegler JS, Kroeze S, Hilbers ML, Imhof L, Guckenberger M, Levesque MP, et al. Toxicity of combined targeted therapy and concurrent radiotherapy in metastatic melanoma patients: a single-center retrospective analysis. Melanoma Res. 2020;30:552-61. DOI: 10.1097/CMR.0000000000000682.
[27] Hecht M, Zimmer L, Loquai C, Weishaupt C, Gutzmer R, Schuster B, et al. Radiosensitization by BRAF inhibitor therapy-mechanism and frequency of toxicity in melanoma patients. Ann Oncol. 2015;26:1238-44. DOI: 10.1093/annonc/mdv139.
[28] Hecht M, Meier F, Zimmer L, Polat B, Loquai C, Weishaupt C, et al. Clinical outcome of concomitant vs interrupted BRAF inhibitor therapy during radiotherapy in melanoma patients. Br J Cancer. 2018;118:785-92. DOI: 10.1038/bjc.2017.489.
[29] Kroeze SGC, Fritz C, Schaule J, Blanck O, Kahl KH, Kaul D, et al. Continued versus Interrupted Targeted Therapy during Metastasis-Directed Stereotactic Radiotherapy: A Retrospective Multi-Center Safety and Efficacy Analysis. Cancers (Basel). 2021;13. DOI: 10.3390/cancers13194780.
[30] Gaudy-Marqueste C, Carron R, Delsanti C, Loundou A, Monestier S, Archier E, et al. On demand Gamma-Knife strategy can be safely combined with BRAF inhibitors for the treatment of melanoma brain metastases. Annals of Oncology. 2014;25:2086-91.
[31] Tetu P, Allayous C, Oriano B, Dalle S, Mortier L, Leccia MT, et al. Impact of radiotherapy administered simultaneously with systemic treatment in patients with melanoma brain metastases within MelBase, a French multicentric prospective cohort. European Journal of Cancer. 2019;112:38-46.
[32] Ahmed KA, Freilich JM, Sloot S, Figura N, Gibney GT, Weber JS, et al. LINAC-based stereotactic radiosurgery to the brain with concurrent vemurafenib for melanoma metastases. J Neurooncol. 2015;122:121-6. DOI: 10.1007/s11060-014-1685-x.

[33] Stera S, Balermpas P, Blanck O, Wolff R, Wurster S, Baumann R, et al. Stereotactic radiosurgery combined with immune checkpoint inhibitors or kinase inhibitors for patients with multiple brain metastases of malignant melanoma. Melanoma Res. 2019;29:187-95. DOI: 10.1097/CMR.0000000000000542.
[34] Patel BG, Ahmed KA, Johnstone PA, Yu HH, Etame AB. Initial experience with combined BRAF and MEK inhibition with stereotactic radiosurgery for BRAF mutant melanoma brain metastases. Melanoma Res. 2016;26:382-6. DOI: 10.1097/CMR.0000000000000250.
[35] Stefan D, Popotte H, Stefan AR, Tesniere A, Tomaszewski A, Lesueur P, et al. Vemurafenib and concomitant stereotactic radiation for the treatment of melanoma with spinal metastases: A case report. Rep Pract Oncol Radiother. 2016;21:76-80. DOI: 10.1016/j.rpor.2015.07.005.
[36] Marti FEM, Jayson GC, Manoharan P, O'Connor J, Renehan AG, Backen AC, et al. Novel phase I trial design to evaluate the addition of cediranib or selumetinib to preoperative chemoradiotherapy for locally advanced rectal cancer: the DREAMtherapy trial. Eur J Cancer. 2019;117:48-59. DOI: 10.1016/j.ejca.2019.04.029.
[37] Wu C, Williams TM, Robb R, Webb A, Wei L, Chen W, et al. Phase I Trial of Trametinib with Neoadjuvant Chemoradiation in Patients with Locally Advanced Rectal Cancer. Clin Cancer Res. 2020;26:3117-25. DOI: 10.1158/1078-0432.CCR-19-4193.
[38] FDA. Highlights of prescribing information: Koselugo. 2020.
[39] FDA. Highlights of prescribing information: Mekinist. 2020.
[40] Ghia AJ, Tward JD, Anker CJ, Boucher KM, Jensen RL, Shrieve DC. Radiosurgery for melanoma brain metastases: the impact of hemorrhage on local control. J Radiosurg SBRT. 2014;3:43-50.
[41] Raizer JJ, Hwu WJ, Panageas KS, Wilton A, Baldwin DE, Bailey E, et al. Brain and leptomeningeal metastases from cutaneous melanoma: survival outcomes based on clinical features. Neuro Oncol. 2008;10:199-207. DOI: 10.1215/15228517-2007-058.
[42] Kotecha R, Miller JA, Venur VA, Mohammadi AM, Chao ST, Suh JH, et al. Melanoma brain metastasis: the impact of stereotactic radiosurgery, BRAF mutational status, and targeted and/or immune-based therapies on treatment outcome. Journal of Neurosurgery. 2018;129:50-9.
[43] Gatterbauer B, Hirschmann D, Eberherr N, Untersteiner H, Cho A, Shaltout A, et al. Toxicity and efficacy of Gamma Knife radiosurgery for brain metastases in melanoma patients treated with immunotherapy or targeted therapy-A retrospective cohort study. Cancer Med. 2020;9:4026-36. DOI: 10.1002/cam4.3021.
[44] Ahmed KA, Abuodeh YA, Echevarria MI, Arrington JA, Stallworth DG, Hogue C, et al. Clinical outcomes of melanoma brain metastases treated with stereotactic radiosurgery and anti-PD-1 therapy, anti-CTLA-4 therapy, BRAF/MEK inhibitors, BRAF inhibitor, or conventional chemotherapy. Ann Oncol. 2016;27:2288-94. DOI: 10.1093/annonc/mdw417.
[45] Xu Z, Lee CC, Ramesh A, Mueller AC, Schlesinger D, Cohen-Inbar O, et al. BRAF V600E mutation and BRAF kinase inhibitors in conjunction with stereotactic radiosurgery for intracranial melanoma metastases. J Neurosurg. 2017;126:726-34. DOI: 10.3171/2016.2.JNS1633.
[46] Ly D, Bagshaw HP, Anker CJ, Tward JD, Grossmann KF, Jensen RL, et al. Local control after stereotactic radiosurgery for brain metastases in patients with melanoma with and without BRAF mutation and treatment. Journal of Neurosurgery. 2015;123:395-401.
[47] Patel KR, Chowdhary M, Switchenko JM, Kudchadkar R, Lawson DH, Cassidy RJ, et al. BRAF inhibitor and stereotactic radiosurgery is associated with an increased risk of radiation necrosis. Melanoma Research. 2016;26:387-94.
[48] Couty E, Vallard A, Sotton S, Ouni S, Garcia MA, Espenel S, et al. Safety assessment of anticancer drugs in association with radiotherapy in metastatic malignant melanoma: a real-life report : Radiation/systemic drug combo in metastatic melanoma. Cancer Chemother Pharmacol. 2019;83:881-92. DOI: 10.1007/s00280-019-03806-5.

[49] Liebner DA, Walston SA, Cavaliere R, Powers CJ, Sauvageau E, Lehman NL, et al. Radiation necrosis mimicking rapid intracranial progression of melanoma metastasis in two patients treated with vemurafenib. Melanoma Res. 2014;24:172-6. DOI: 10.1097/CMR.0000000000000044.
[50] Marquez-Rodas I, Aviles-Izquierdo JA, Parra V, Alvarez-Gonzalez A, Borrego P, Fernandez-Garcia P, et al. Exclusion criteria vs reality: dual BRAF/MEK inhibition and radiotherapy in a patient with melanoma metastatic to the brain and ECOG 3. Tumori. 2016;102:11. DOI: 10.5301/tj.5000408.
[51] Flaum N, Lorigan P, Whitfield GA, Hawkins RE, Pinkham MB. Integrating radiation therapy with emerging systemic therapies: Lessons from a patient with cerebral radionecrosis, spinal cord myelopathy, and radiation pneumonitis. Pract Radiat Oncol. 2016;6:110-3. DOI: 10.1016/j.prro.2015.10.008.
