# Supplementary material for: ESMO-ESTRO consensus statements on the safety of combining radiotherapy with EGFR, ALK, or BRAF/MEK inhibitors
Source: ESMO Open. 2026 Feb 26;11(3):106076. doi: 10.1016/j.esmoop.2026.106076 (PMC12955645; doi:10.1016/j.esmoop.2026.106076)
Supplement: Supplementary Tables [file mmc3.pdf]

Supplementary Tables

This document provides additional data for the ESMO-ESTRO consensus statements on the safety of combining radiotherapy with EGFR, ALK, or BRAF/MEK inhibitors.

Table of Contents

Table S1. Search strategy for EGFR inhibitors. 2

Table S2. Search strategy for ALK inhibitors. 6

Table S3. Search strategy for BRAF inhibitors. 10

Table S4. Search strategy for MEK inhibitors. 14

Table S5. Inclusion and exclusion criteria. 18

Table S6. Levels of evidence. 19

Table S7. Delphi round one results and decisions for EGFR inhibitors. 20

Table S8. Delphi round two results for EGFR inhibitors. 22

Table S9. Delphi round one results and decisions for ALK inhibitors. 23

Table S10. Delphi round two results for ALK inhibitors. 25

Table S11. Delphi round one results and decisions for BRAF/MEK inhibitors. 26

Table S12. Delphi round two results for BRAF/MEK inhibitors. 27

Table S1. Search strategy for EGFR inhibitors.

Medline (Ovid)

Topic	Search strategy
Radiotherapy	exp *radiotherapy/ or (radiotherap* or radiation or irradiat* or gammaknife or "gamma knife" or cyberknife or "cyber knife" or stereotactic or SRS or SRT or SBRT or radiosurger* or chemoradi* or radiochemo* or radioimmuno* or radiosens* or radioresist*).ti. or (ablati*.ti. and (radiotherap* or radiation or irradiat* or gammaknife or "gamma knife" or cyberknife or "cyber knife" or stereotactic or SRS or SRT or SBRT or radiosurger* or chemoradi* or radiochemo* or radioimmuno* or radiosens*).ti,ab,kf.)
Toxicity	Drug-Related Side Effects and Adverse Reactions/ or clinical trials, phase i as topic/ or clinical trials, phase ii as topic/ or clinical trials, phase iii as topic/ or (clinical trial phase i or clinical trial phase ii or clinical trial phase iii).pt. or (toxicit* or feasibility or "side effect*" or "adverse effect*" or "adverse event*" or DLT or "dose limiting" or "maximum tolerated dose" or morbidity or safety or safe or safely or risk or tolerance or injury or complication* or fatigue or radionecrosis or necrosis or hemorrhag* or haemorrhag* or bleeding or effusion or pain or fracture or osteoradionecrosis or perforation or ulcer or ulceration or fistula or fibrosis or stenosis or obstruction or atrophy or induration or mucositis or edema or oedema or rash or dermatitis or erythema or folliculitis or FCP or "cutis verticis gyrata" or CVG or myelitis or myelopathy or neurotoxicity or neurotoxic or neuropathy or neurocognit* or cognit* or "memory loss" or Lhermitte or encephalopathy or alopecia or xerostomia or stomatitis or hemoptysis or haemoptysis or pneumonitis or cough or dyspnoea or dyspnea or esophagitis or oesophagitis or dysphagia or nausea or vomiting or hematemesis or haematemesis or hepatitis or cardiotoxic* or "renal failure" or atherosclerosis or nephropathy or nephrotoxic* or cystitis or hematuria or haematuria or incontinence or colitis or enteritis or diarrhea or diarrhoea or proctitis or "radiation recall" or otitis or epidermolysis or "phase 1*" or "phase i" or "phase ia*" or "phase ib*" or "phase 2*" or "phase ii" or "phase iia*" or "phase iib*" or "phase 3*" or "phase iii").ti,ab,kf.
EGFR (version 1)	(("Epithelial Growth Factor Receptor*" or EGFR* or "EGF receptor*") adj4 (inhibit* or anti)).ti,ab,kf. or (cetuximab or erbitux or panitumumab or vectibix or gefitinib or iressa or erlotinib or tarceva or osimertinib or tagrisso or afatinib or giotrif or icotinib or conmana or portrazza or necitumumab).ti,ab.
EGFR (version 2)	(("Epithelial Growth Factor Receptor*" or "epidermal growth factor receptor*" or EGFR* or "EGF receptor*") adj4 (inhibit* or anti)).ti,ab,kf. or (egfr-tki or cetuximab or erbitux or panitumumab or vectibix or gefitinib or iressa or erlotinib or tarceva or osimertinib or tagrisso or afatinib or giotrif or icotinib or conmana or portrazza or necitumumab or nimotuzumab or theraloc).ti,ab.

Final search	Records containing Radiotherapy *and* Toxicity *and* EGFR search terms
**Embase (Ovid)**	
**Topic**	**Search strategy**
Radiotherapy	exp *radiotherapy/ or (radiotherap* or radiation or irradiat* or gammaknife or "gamma knife" or cyberknife or "cyber knife" or stereotactic or SRS or SRT or SBRT or radiosurger* or chemoradi* or radiochemo* or radioimmuno* or radiosens* or radioresist*).ti. or (ablati*.ti. and (radiotherap* or radiation or irradiat* or gammaknife or "gamma knife" or cyberknife or "cyber knife" or stereotactic or SRS or SRT or SBRT or radiosurger* or chemoradi* or radiochemo* or radioimmuno* or radiosens*).ti,ab,kw.)
Toxicity	side effect/ or adverse event/ or "phase 1 clinical trial (topic)"/ or "phase 2 clinical trial (topic)"/ or "phase 3 clinical trial (topic)"/ or exp phase 1 clinical trial/ or exp phase 2 clinical trial/ or exp phase 3 clinical trial/ or (toxicit* or feasibility or "side effect*" or "adverse effect*" or "adverse event*" or DLT or "dose limiting" or "maximum tolerated dose" or morbidity or safety or safe or safely or risk or tolerance or injury or complication* or fatigue or radionecrosis or necrosis or hemorrhag* or haemorrhag* or bleeding or effusion or pain or fracture or osteoradionecrosis or perforation or ulcer or ulceration or fistula or fibrosis or stenosis or obstruction or atrophy or induration or mucositis or edema or oedema or rash or dermatitis or erythema or folliculitis or FCP or "cutis verticis gyrata" or CVG or myelitis or myelopathy or neurotoxicity or neurotoxic or neuropathy or neurocognit* or cognit* or "memory loss" or Lhermitte or encephalopathy or alopecia or xerostomia or stomatitis or hemoptysis or haemoptysis or pneumonitis or cough or dyspnoea or dyspnea or esophagitis or oesophagitis or dysphagia or nausea or vomiting or hematemesis or haematemesis or hepatitis or cardiotoxic* or "renal failure" or atherosclerosis or nephropathy or nephrotoxic* or cystitis or hematuria or haematuria or incontinence or colitis or enteritis or diarrhea or diarrhoea or proctitis or "radiation recall" or otitis or epidermolysis or "phase 1*" or "phase i" or "phase ia*" or "phase ib*" or "phase 2*" or "phase ii" or "phase iia*" or "phase iib*" or "phase 3*" or "phase iii").ti,ab,kw.
EGFR (version 1)	(("Epithelial Growth Factor Receptor*" or EGFR* or "EGF receptor*") adj4 (inhibit* or anti)).ti,ab,kw. or (cetuximab or erbitux or panitumumab or vectibix or gefitinib or iressa or erlotinib or tarceva or osimertinib or tagrisso or afatinib or giotrif or icotinib or conmana or portrazza or necitumumab).ti,ab.
EGFR (version 2)	(("Epithelial Growth Factor Receptor*" or "epidermal growth factor receptor*" or EGFR* or "EGF receptor*") adj4 (inhibit* or anti)).ti,ab,kw. or (egfr-tki or cetuximab or erbitux or panitumumab or vectibix or gefitinib or iressa or erlotinib or tarceva or osimertinib or tagrisso or afatinib or giotrif or icotinib or conmana or portrazza or necitumumab or nimotuzumab or theraloc).ti,ab.

Final search	Records containing Radiotherapy *and* Toxicity *and* EGFR search terms
**Scopus**	
**Topic**	**Search strategy**
Radiotherapy	TITLE(radiotherap* OR radiation OR irradiat* OR gammaknife OR "gamma knife" OR cyberknife OR "cyber knife" OR stereotactic OR SRS OR SRT OR SBRT OR radiosurger* OR chemoradi* OR radiochemo* OR radioimmuno* OR radiosens* OR radioresist*) OR (TITLE(ablati*) AND TITLE-ABS(radiotherap* OR radiation OR irradiat* OR gammaknife OR "gamma knife" OR cyberknife OR "cyber knife" OR stereotactic OR SRS OR SRT OR SBRT OR radiosurger* OR chemoradi* OR radiochemo* OR radioimmuno* OR radiosens*)) OR (TITLE(ablati*) AND AUTHKEY(radiotherap* OR radiation OR irradiat* OR gammaknife OR "gamma knife" OR cyberknife OR "cyber knife" OR stereotactic OR SRS OR SRT OR SBRT OR radiosurger* OR chemoradi* OR radiochemo* OR radioimmuno* OR radiosens*))
Toxicity	TITLE-ABS (toxicit* or feasibility or "side effect*" or "adverse effect*" or "adverse event*" or DLT or "dose limiting" or "maximum tolerated dose" or morbidity or safety or safe or safely or risk or tolerance or injury or complication* or fatigue or radionecrosis or necrosis or hemorrhag* or haemorrhag* or bleeding or effusion or pain or fracture or osteoradionecrosis or perforation or ulcer or ulceration or fistula or fibrosis or stenosis or obstruction or atrophy or induration or mucositis or edema or oedema or rash or dermatitis or erythema or folliculitis or FCP or "cutis verticis gyrata" or CVG or myelitis or myelopathy or neurotoxicity or neurotoxic or neuropathy or neurocognit* or cognit* or "memory loss" or Lhermitte or encephalopathy or alopecia or xerostomia or stomatitis or hemoptysis or haemoptysis or pneumonitis or cough or dyspnoea or dyspnea or esophagitis or oesophagitis or dysphagia or nausea or vomiting or hematemesis or haematemesis or hepatitis or cardiotoxic* or "renal failure" or atherosclerosis or nephropathy or nephrotoxic* or cystitis or hematuria or haematuria or incontinence or colitis or enteritis or diarrhea or diarrhoea or proctitis or "radiation recall" or otitis or epidermolysis or "phase 1*" OR "phase i" OR "phase ia*" OR "phase ib*" OR "phase 2*" OR "phase ii" OR "phase iia*" OR "phase iib*" OR "phase 3*" OR "phase iii") or AUTHKEY (toxicit* or feasibility or "side effect*" or "adverse effect*" or "adverse event*" or DLT or "dose limiting" or "maximum tolerated dose" or morbidity or safety or safe or safely or risk or tolerance or injury or complication* or fatigue or radionecrosis or necrosis or hemorrhag* or haemorrhag* or bleeding or effusion or pain or fracture or osteoradionecrosis or perforation or ulcer or ulceration or fistula or fibrosis or stenosis or obstruction or atrophy or induration or mucositis or edema or oedema or rash or dermatitis or erythema or folliculitis or FCP or "cutis verticis gyrata" or CVG or myelitis or myelopathy or

	neurotoxicity or neurotoxic or neuropathy or neurocognit* or cognit* or "memory loss" or Lhermitte or encephalopathy or alopecia or xerostomia or stomatitis or hemoptysis or haemoptysis or pneumonitis or cough or dyspnoea or dyspnea or esophagitis or oesophagitis or dysphagia or nausea or vomiting or hematemesis or haematemesis or hepatitis or cardiotoxic* or "renal failure" or atherosclerosis or nephropathy or nephrotoxic* or cystitis or hematuria or haematuria or incontinence or colitis or enteritis or diarrhea or diarrhoea or proctitis or "radiation recall" or otitis or epidermolysis or "phase 1*" OR "phase i" OR "phase ia*" OR "phase ib*" OR "phase 2*" OR "phase ii" OR "phase iia*" OR "phase iib*" OR "phase 3*" OR "phase iii")
EGFR (version 1)	(TITLE-ABS (("Epithelial Growth Factor Receptor*" OR egfr* OR "EGF receptor*") W/4 (inhibit* OR anti)) OR AUTHKEY (("Epithelial Growth Factor Receptor*" OR egfr* OR "EGF receptor*") W/4 (inhibit* OR anti)) OR TITLE-ABS (cetuximab OR erbitux OR panitumumab OR vectibix OR gefitinib OR iressa OR erlotinib OR tarceva OR osimertinib OR tagrisso OR afatinib OR giotrif OR icotinib OR conmana OR portrazza OR necitumumab)))
EGFR (version 2)	(TITLE-ABS (("Epithelial Growth Factor Receptor*" OR "epidermal growth factor receptor*" OR egfr* OR "EGF receptor*") W/4 (inhibit* OR anti)) OR AUTHKEY (("Epithelial Growth Factor Receptor*" OR egfr* OR "EGF receptor*") W/4 (inhibit* OR anti)) OR TITLE-ABS (egfr-tki OR cetuximab OR erbitux OR panitumumab OR vectibix OR gefitinib OR iressa OR erlotinib OR tarceva OR osimertinib OR tagrisso OR afatinib OR giotrif OR icotinib OR conmana OR portrazza OR necitumumab OR nimotuzumab OR theraloc)))
Final search	Records containing Radiotherapy *and* Toxicity *and* EGFR search terms

Table S2. Search strategy for ALK inhibitors.

Medline (Ovid)

**Topic**	**Search strategy**
Radiotherapy	exp *radiotherapy/ OR (radiotherap* OR radiation OR irradiat* OR gammaknife OR "gamma knife" OR cyberknife OR "cyber knife" OR stereotactic OR SRS OR SRT OR SBRT OR radiosurger* OR chemoradi* OR radiochemo* OR radioimmuno* OR radiosens* OR radioresist*).ti. OR (ablati*.ti. AND (radiotherap* OR radiation OR irradiat* OR gammaknife OR "gamma knife" OR cyberknife OR "cyber knife" OR stereotactic OR SRS OR SRT OR SBRT OR radiosurger* OR chemoradi* OR radiochemo* OR radioimmuno* OR radiosens*).ti,ab,kf.)
Toxicity	"Drug-Related Side Effects and Adverse Reactions"/ or clinical trials, phase i as topic/ or clinical trials, phase ii as topic/ or clinical trials, phase iii as topic/ or (clinical trial phase i or clinical trial phase ii or clinical trial phase iii).pt. or (toxicit* or feasibility or "side effect*" or "adverse effect*" or "adverse event*" or DLT or "dose limiting" or "maximum tolerated dose" or morbidity or safety or safe or safely or risk or tolerance or injury or complication* or fatigue or radionecrosis or necrosis or hemorrhag* or haemorrhag* or bleeding or effusion or pain or fracture or osteoradionecrosis or perforation or ulcer or ulceration or fistula or fibrosis or stenosis or obstruction or atrophy or induration or mucositis or edema or oedema or rash or dermatitis or erythema or folliculitis or FCP or "cutis verticis gyrata" or CVG or myelitis or myelopathy or neurotoxicity or neurotoxic or neuropathy or neurocognit* or cognit* or "memory loss" or Lhermitte or encephalopathy or alopecia or xerostomia or stomatitis or hemoptysis or haemoptysis or pneumonitis or cough or dyspnoea or dyspnea or esophagitis or oesophagitis or dysphagia or nausea or vomiting or hematemesis or haematemesis or hepatitis or cardiotoxic* or "renal failure" or atherosclerosis or nephropathy or nephrotoxic* or cystitis or hematuria or haematuria or incontinence or colitis or enteritis or diarrhea or diarrhoea or proctitis or "radiation recall" or otitis or epidermolysis or "phase 1*" or "phase i" or "phase ia*" or "phase ib*" or "phase 2*" or "phase ii" or "phase iia*" or "phase iib*" or "phase 3*" or "phase iii").ti,ab,kf.
ALK	(anaplastic lymphoma kinase/ AND protein kinase inhibitors/) OR (("anaplastic lymphoma kinase" OR ALK) ADJ4 (inhibit* OR anti)).ti,ab,kf. OR (crizotinib OR xalkori OR ceritinib OR zykadia OR alectinib OR alecensa OR ensartinib OR brigatinib OR alunbrig OR lorlatinib OR lorbrena).ti,ab.
Final search	Records containing Radiotherapy *and* Toxicity *and* ALK search terms

Embase (Ovid)

**Topic**	**Search strategy**
Radiotherapy	exp *radiotherapy/ OR (radiotherap* OR radiation OR irradiat* OR gammaknife OR "gamma knife" OR cyberknife OR "cyber knife" OR

	stereotactic OR SRS OR SRT OR SBRT OR radiosurger* OR chemoradi* OR radiochemo* OR radioimmuno* OR radiosens* OR radioresist*).ti. OR (ablati*.ti. AND (radiotherap* OR radiation OR irradiat* OR gammaknife OR "gamma knife" OR cyberknife OR "cyber knife" OR stereotactic OR SRS OR SRT OR SBRT OR radiosurger* OR chemoradi* OR radiochemo* OR radioimmuno* OR radiosens*).ti,ab,kw.)
Toxicity	side effect/ or adverse event/ or "phase 1 clinical trial (topic)"/ or "phase 2 clinical trial (topic)"/ or "phase 3 clinical trial (topic)"/ or exp phase 1 clinical trial/ or exp phase 2 clinical trial/ or exp phase 3 clinical trial/ or (toxicit* or feasibility or "side effect*" or "adverse effect*" or "adverse event*" or DLT or "dose limiting" or "maximum tolerated dose" or morbidity or safety or safe or safely or risk or tolerance or injury or complication* or fatigue or radionecrosis or necrosis or hemorrhag* or haemorrhag* or bleeding or effusion or pain or fracture or osteoradionecrosis or perforation or ulcer or ulceration or fistula or fibrosis or stenosis or obstruction or atrophy or induration or mucositis or edema or oedema or rash or dermatitis or erythema or folliculitis or FCP or "cutis verticis gyrata" or CVG or myelitis or myelopathy or neurotoxicity or neurotoxic or neuropathy or neurocognit* or cognit* or "memory loss" or Lhermitte or encephalopathy or alopecia or xerostomia or stomatitis or hemoptysis or haemoptysis or pneumonitis or cough or dyspnoea or dyspnea or esophagitis or oesophagitis or dysphagia or nausea or vomiting or hematemesis or haematemesis or hepatitis or cardiotoxic* or "renal failure" or atherosclerosis or nephropathy or nephrotoxic* or cystitis or hematuria or haematuria or incontinence or colitis or enteritis or diarrhea or diarrhoea or proctitis or "radiation recall" or otitis or epidermolysis or "phase 1*" or "phase i" or "phase ia*" or "phase ib*" or "phase 2*" or "phase ii" or "phase iia*" or "phase iib*" or "phase 3*" or "phase iii").ti,ab,kw.
ALK	(anaplastic lymphoma kinase/ AND protein kinase inhibitors/) OR (("anaplastic lymphoma kinase" OR ALK) ADJ4 (inhibit* OR anti)).ti,ab,kw. OR (crizotinib OR xalkori OR ceritinib OR zykadia OR alectinib OR alecensa OR ensartinib OR brigatinib OR alunbrig OR lorlatinib OR lorbrena).ti,ab.
Final search	Records containing Radiotherapy *and* Toxicity *and* ALK search terms
**Scopus**	
**Topic**	**Search strategy**
Radiotherapy	TITLE(radiotherap* OR radiation OR irradiat* OR gammaknife OR "gamma knife" OR cyberknife OR "cyber knife" OR stereotactic OR SRS OR SRT OR SBRT OR radiosurger* OR chemoradi* OR radiochemo* OR radioimmuno* OR radiosens* OR radioresist*) OR (TITLE(ablati*) AND TITLE-ABS(radiotherap* OR radiation OR irradiat* OR gammaknife OR "gamma knife" OR cyberknife OR "cyber knife" OR stereotactic OR SRS OR SRT OR SBRT OR radiosurger* OR chemoradi* OR radiochemo* OR radioimmuno*

	OR radiosens*)) OR (TITLE(ablati*) AND AUTHKEY(radiotherap* OR radiation OR irradiat* OR gammaknife OR "gamma knife" OR cyberknife OR "cyber knife" OR stereotactic OR SRS OR SRT OR SBRT OR radiosurger* OR chemoradi* OR radiochemo* OR radioimmuno* OR radiosens*))
Toxicity	TITLE-ABS (toxicit* or feasibility or "side effect*" or "adverse effect*" or "adverse event*" or DLT or "dose limiting" or "maximum tolerated dose" or morbidity or safety or safe or safely or risk or tolerance or injury or complication* or fatigue or radionecrosis or necrosis or hemorrhag* or haemorrhag* or bleeding or effusion or pain or fracture or osteoradionecrosis or perforation or ulcer or ulceration or fistula or fibrosis or stenosis or obstruction or atrophy or induration or mucositis or edema or oedema or rash or dermatitis or erythema or folliculitis or FCP or "cutis verticis gyrata" or CVG or myelitis or myelopathy or neurotoxicity or neurotoxic or neuropathy or neurocognit* or cognit* or "memory loss" or Lhermitte or encephalopathy or alopecia or xerostomia or stomatitis or hemoptysis or haemoptysis or pneumonitis or cough or dyspnoea or dyspnea or esophagitis or oesophagitis or dysphagia or nausea or vomiting or hematemesis or haematemesis or hepatitis or cardiotoxic* or "renal failure" or atherosclerosis or nephropathy or nephrotoxic* or cystitis or hematuria or haematuria or incontinence or colitis or enteritis or diarrhea or diarrhoea or proctitis or "radiation recall" or otitis or epidermolysis or "phase 1*" OR "phase i" OR "phase ia*" OR "phase ib*" OR "phase 2*" OR "phase ii" OR "phase iia*" OR "phase iib*" OR "phase 3*" OR "phase iii") or AUTHKEY (toxicit* or feasibility or "side effect*" or "adverse effect*" or "adverse event*" or DLT or "dose limiting" or "maximum tolerated dose" or morbidity or safety or safe or safely or risk or tolerance or injury or complication* or fatigue or radionecrosis or necrosis or hemorrhag* or haemorrhag* or bleeding or effusion or pain or fracture or osteoradionecrosis or perforation or ulcer or ulceration or fistula or fibrosis or stenosis or obstruction or atrophy or induration or mucositis or edema or oedema or rash or dermatitis or erythema or folliculitis or FCP or "cutis verticis gyrata" or CVG or myelitis or myelopathy or neurotoxicity or neurotoxic or neuropathy or neurocognit* or cognit* or "memory loss" or Lhermitte or encephalopathy or alopecia or xerostomia or stomatitis or hemoptysis or haemoptysis or pneumonitis or cough or dyspnoea or dyspnea or esophagitis or oesophagitis or dysphagia or nausea or vomiting or hematemesis or haematemesis or hepatitis or cardiotoxic* or "renal failure" or atherosclerosis or nephropathy or nephrotoxic* or cystitis or hematuria or haematuria or incontinence or colitis or enteritis or diarrhea or diarrhoea or proctitis or "radiation recall" or otitis or epidermolysis or "phase 1*" OR "phase i" OR "phase ia*" OR "phase ib*" OR "phase 2*" OR "phase ii" OR "phase iia*" OR "phase iib*" OR "phase 3*" OR "phase iii")

ALK	TITLE-ABS(("anaplastic lymphoma kinase" OR ALK) W/4 (inhibit* OR anti)) OR AUTHKEY(("anaplastic lymphoma kinase" OR ALK) W/4 (inhibit* OR anti)) OR TITLE-ABS(crizotinib OR xalkori OR ceritinib OR zykadia OR alectinib OR alecensa OR ensartinib OR brigatinib OR alunbrig OR lorlatinib OR lorbrena)
Final search	Records containing Radiotherapy *and* Toxicity *and* ALK search terms

Table S3. Search strategy for BRAF inhibitors.

Medline (Ovid)

**Topic**	**Search strategy**
Radiotherapy	exp *radiotherapy/ OR (radiotherap* OR radiation OR irradiat* OR gammaknife OR "gamma knife" OR cyberknife OR "cyber knife" OR stereotactic OR SRS OR SRT OR SBRT OR radiosurger* OR chemoradi* OR radiochemo* OR radioimmuno* OR radiosens* OR radioresist*).ti. OR (ablati*.ti. AND (radiotherap* OR radiation OR irradiat* OR gammaknife OR "gamma knife" OR cyberknife OR "cyber knife" OR stereotactic OR SRS OR SRT OR SBRT OR radiosurger* OR chemoradi* OR radiochemo* OR radioimmuno* OR radiosens*).ti,ab,kf.)
Toxicity	"Drug-Related Side Effects and Adverse Reactions"/ or clinical trials, phase i as topic/ or clinical trials, phase ii as topic/ or clinical trials, phase iii as topic/ or (clinical trial phase i or clinical trial phase ii or clinical trial phase iii).pt. or (toxicit* or feasibility or "side effect*" or "adverse effect*" or "adverse event*" or DLT or "dose limiting" or "maximum tolerated dose" or morbidity or safety or safe or safely or risk or tolerance or injury or complication* or fatigue or radionecrosis or necrosis or hemorrhag* or haemorrhag* or bleeding or effusion or pain or fracture or osteoradionecrosis or perforation or ulcer or ulceration or fistula or fibrosis or stenosis or obstruction or atrophy or induration or mucositis or edema or oedema or rash or dermatitis or erythema or folliculitis or FCP or "cutis verticis gyrata" or CVG or myelitis or myelopathy or neurotoxicity or neurotoxic or neuropathy or neurocognit* or cognit* or "memory loss" or Lhermitte or encephalopathy or alopecia or xerostomia or stomatitis or hemoptysis or haemoptysis or pneumonitis or cough or dyspnoea or dyspnea or esophagitis or oesophagitis or dysphagia or nausea or vomiting or hematemesis or haematemesis or hepatitis or cardiotoxic* or "renal failure" or atherosclerosis or nephropathy or nephrotoxic* or cystitis or hematuria or haematuria or incontinence or colitis or enteritis or diarrhea or diarrhoea or proctitis or "radiation recall" or otitis or epidermolysis or "phase 1*" or "phase i" or "phase ia*" or "phase ib*" or "phase 2*" or "phase ii" or "phase iia*" or "phase iib*" or "phase 3*" or "phase iii").ti,ab,kf.
BRAF	(Proto-Oncogene Proteins B-raf/ AND protein kinase inhibitors/) OR ((BRAF OR "B-RAF") ADJ4 (inhibit* OR anti)).ti,ab,kf. OR (vemurafenib OR zelboraf OR dabrafenib OR tafinlar OR encorafenib OR braftovi).ti,ab.
Final search	Records containing Radiotherapy *and* Toxicity *and* BRAF search terms

Embase (Ovid)

**Topic**	**Search strategy**
Radiotherapy	exp *radiotherapy/ OR (radiotherap* OR radiation OR irradiat* OR gammaknife OR "gamma knife" OR cyberknife OR "cyber knife" OR

	stereotactic OR SRS OR SRT OR SBRT OR radiosurger* OR chemoradi* OR radiochemo* OR radioimmuno* OR radiosens* OR radioresist*).ti. OR (ablati*.ti. AND (radiotherap* OR radiation OR irradiat* OR gammaknife OR "gamma knife" OR cyberknife OR "cyber knife" OR stereotactic OR SRS OR SRT OR SBRT OR radiosurger* OR chemoradi* OR radiochemo* OR radioimmuno* OR radiosens*).ti,ab,kw.)
Toxicity	side effect/ or adverse event/ or "phase 1 clinical trial (topic)"/ or "phase 2 clinical trial (topic)"/ or "phase 3 clinical trial (topic)"/ or exp phase 1 clinical trial/ or exp phase 2 clinical trial/ or exp phase 3 clinical trial/ or (toxicit* or feasibility or "side effect*" or "adverse effect*" or "adverse event*" or DLT or "dose limiting" or "maximum tolerated dose" or morbidity or safety or safe or safely or risk or tolerance or injury or complication* or fatigue or radionecrosis or necrosis or hemorrhag* or haemorrhag* or bleeding or effusion or pain or fracture or osteoradionecrosis or perforation or ulcer or ulceration or fistula or fibrosis or stenosis or obstruction or atrophy or induration or mucositis or edema or oedema or rash or dermatitis or erythema or folliculitis or FCP or "cutis verticis gyrata" or CVG or myelitis or myelopathy or neurotoxicity or neurotoxic or neuropathy or neurocognit* or cognit* or "memory loss" or Lhermitte or encephalopathy or alopecia or xerostomia or stomatitis or hemoptysis or haemoptysis or pneumonitis or cough or dyspnoea or dyspnea or esophagitis or oesophagitis or dysphagia or nausea or vomiting or hematemesis or haematemesis or hepatitis or cardiotoxic* or "renal failure" or atherosclerosis or nephropathy or nephrotoxic* or cystitis or hematuria or haematuria or incontinence or colitis or enteritis or diarrhea or diarrhoea or proctitis or "radiation recall" or otitis or epidermolysis or "phase 1*" or "phase i" or "phase ia*" or "phase ib*" or "phase 2*" or "phase ii" or "phase iia*" or "phase iib*" or "phase 3*" or "phase iii").ti,ab,kw.
BRAF	exp B Raf kinase inhibitor/ OR ((BRAF OR "B-RAF") ADJ4 (inhibit* OR anti)).ti,ab,kw. OR (vemurafenib OR zelboraf OR dabrafenib OR tafinlar OR encorafenib OR braftovi).ti,ab.
Final search	Records containing Radiotherapy *and* Toxicity *and* BRAF search terms
**Scopus**	
**Topic**	**Search strategy**
Radiotherapy	TITLE(radiotherap* OR radiation OR irradiat* OR gammaknife OR "gamma knife" OR cyberknife OR "cyber knife" OR stereotactic OR SRS OR SRT OR SBRT OR radiosurger* OR chemoradi* OR radiochemo* OR radioimmuno* OR radiosens* OR radioresist*) OR (TITLE(ablati*) AND TITLE-ABS(radiotherap* OR radiation OR irradiat* OR gammaknife OR "gamma knife" OR cyberknife OR "cyber knife" OR stereotactic OR SRS OR SRT OR SBRT OR radiosurger* OR chemoradi* OR radiochemo* OR radioimmuno* OR radiosens*)) OR (TITLE(ablati*) AND AUTHKEY(radiotherap* OR

	radiation OR irradiat* OR gammaknife OR "gamma knife" OR cyberknife OR "cyber knife" OR stereotactic OR SRS OR SRT OR SBRT OR radiosurger* OR chemoradi* OR radiochemo* OR radioimmuno* OR radiosens*))
Toxicity	TITLE-ABS (toxicit* or feasibility or "side effect*" or "adverse effect*" or "adverse event*" or DLT or "dose limiting" or "maximum tolerated dose" or morbidity or safety or safe or safely or risk or tolerance or injury or complication* or fatigue or radionecrosis or necrosis or hemorrhag* or haemorrhag* or bleeding or effusion or pain or fracture or osteoradionecrosis or perforation or ulcer or ulceration or fistula or fibrosis or stenosis or obstruction or atrophy or induration or mucositis or edema or oedema or rash or dermatitis or erythema or folliculitis or FCP or "cutis verticis gyrata" or CVG or myelitis or myelopathy or neurotoxicity or neurotoxic or neuropathy or neurocognit* or cognit* or "memory loss" or Lhermitte or encephalopathy or alopecia or xerostomia or stomatitis or hemoptysis or haemoptysis or pneumonitis or cough or dyspnoea or dyspnea or esophagitis or oesophagitis or dysphagia or nausea or vomiting or hematemesis or haematemesis or hepatitis or cardiotoxic* or "renal failure" or atherosclerosis or nephropathy or nephrotoxic* or cystitis or hematuria or haematuria or incontinence or colitis or enteritis or diarrhea or diarrhoea or proctitis or "radiation recall" or otitis or epidermolysis or "phase 1*" OR "phase i" OR "phase ia*" OR "phase ib*" OR "phase 2*" OR "phase ii" OR "phase iia*" OR "phase iib*" OR "phase 3*" OR "phase iii") or AUTHKEY (toxicit* or feasibility or "side effect*" or "adverse effect*" or "adverse event*" or DLT or "dose limiting" or "maximum tolerated dose" or morbidity or safety or safe or safely or risk or tolerance or injury or complication* or fatigue or radionecrosis or necrosis or hemorrhag* or haemorrhag* or bleeding or effusion or pain or fracture or osteoradionecrosis or perforation or ulcer or ulceration or fistula or fibrosis or stenosis or obstruction or atrophy or induration or mucositis or edema or oedema or rash or dermatitis or erythema or folliculitis or FCP or "cutis verticis gyrata" or CVG or myelitis or myelopathy or neurotoxicity or neurotoxic or neuropathy or neurocognit* or cognit* or "memory loss" or Lhermitte or encephalopathy or alopecia or xerostomia or stomatitis or hemoptysis or haemoptysis or pneumonitis or cough or dyspnoea or dyspnea or esophagitis or oesophagitis or dysphagia or nausea or vomiting or hematemesis or haematemesis or hepatitis or cardiotoxic* or "renal failure" or atherosclerosis or nephropathy or nephrotoxic* or cystitis or hematuria or haematuria or incontinence or colitis or enteritis or diarrhea or diarrhoea or proctitis or "radiation recall" or otitis or epidermolysis or "phase 1*" OR "phase i" OR "phase ia*" OR "phase ib*" OR "phase 2*" OR "phase ii" OR "phase iia*" OR "phase iib*" OR "phase 3*" OR "phase iii")

BRAF	TITLE-ABS((BRAF OR "B-RAF") W/4 (inhibit* OR anti)) OR AUTHKEY((BRAF OR "B-RAF") W/4 (inhibit* OR anti)) OR TITLE-ABS(vemurafenib OR zelboraf OR dabrafenib OR tafinlar OR encorafenib OR braftovi)
Final search	Records containing Radiotherapy *and* Toxicity *and* BRAF search terms

Table S4. Search strategy for MEK inhibitors.

Medline (Ovid)

**Topic**	**Search strategy**
Radiotherapy	exp *radiotherapy/ OR (radiotherap* OR radiation OR irradiat* OR gammaknife OR "gamma knife" OR cyberknife OR "cyber knife" OR stereotactic OR SRS OR SRT OR SBRT OR radiosurger* OR chemoradi* OR radiochemo* OR radioimmuno* OR radiosens* OR radioresist*).ti. OR (ablati*.ti. AND (radiotherap* OR radiation OR irradiat* OR gammaknife OR "gamma knife" OR cyberknife OR "cyber knife" OR stereotactic OR SRS OR SRT OR SBRT OR radiosurger* OR chemoradi* OR radiochemo* OR radioimmuno* OR radiosens*).ti,ab,kf.)
Toxicity	"Drug-Related Side Effects and Adverse Reactions"/ or clinical trials, phase i as topic/ or clinical trials, phase ii as topic/ or clinical trials, phase iii as topic/ or (clinical trial phase i or clinical trial phase ii or clinical trial phase iii).pt. or (toxicit* or feasibility or "side effect*" or "adverse effect*" or "adverse event*" or DLT or "dose limiting" or "maximum tolerated dose" or morbidity or safety or safe or safely or risk or tolerance or injury or complication* or fatigue or radionecrosis or necrosis or hemorrhag* or haemorrhag* or bleeding or effusion or pain or fracture or osteoradionecrosis or perforation or ulcer or ulceration or fistula or fibrosis or stenosis or obstruction or atrophy or induration or mucositis or edema or oedema or rash or dermatitis or erythema or folliculitis or FCP or "cutis verticis gyrata" or CVG or myelitis or myelopathy or neurotoxicity or neurotoxic or neuropathy or neurocognit* or cognit* or "memory loss" or Lhermitte or encephalopathy or alopecia or xerostomia or stomatitis or hemoptysis or haemoptysis or pneumonitis or cough or dyspnoea or dyspnea or esophagitis or oesophagitis or dysphagia or nausea or vomiting or hematemesis or haematemesis or hepatitis or cardiotoxic* or "renal failure" or atherosclerosis or nephropathy or nephrotoxic* or cystitis or hematuria or haematuria or incontinence or colitis or enteritis or diarrhea or diarrhoea or proctitis or "radiation recall" or otitis or epidermolysis or "phase 1*" or "phase i" or "phase ia*" or "phase ib*" or "phase 2*" or "phase ii" or "phase iia*" or "phase iib*" or "phase 3*" or "phase iii").ti,ab,kf.
MEK	((exp Mitogen-Activated Protein Kinase Kinases/ OR exp Mitogen-Activated Protein Kinases/ OR exp MAP Kinase Kinase Kinases/) AND protein kinase inhibitors/) OR ((MEK OR "mitogen-activated protein kinase*" OR MEK1 OR MEK2 OR MAP OR MAPKK*).ti,ab,kf. ADJ4 (inhibit* OR anti).ti,ab,kf.) OR (MEKi).ti,ab,kf. OR (trametinib OR mekinist OR cobimetinib OR cotellic OR binimetinib OR mektovi).ti,ab.
Final search	Records containing Radiotherapy *and* Toxicity *and* MEK search terms

Embase (Ovid)

Topic	Search strategy
Radiotherapy	exp *radiotherapy/ OR (radiotherap* OR radiation OR irradiat* OR gammaknife OR "gamma knife" OR cyberknife OR "cyber knife" OR stereotactic OR SRS OR SRT OR SBRT OR radiosurger* OR chemoradi* OR radiochemo* OR radioimmuno* OR radiosens* OR radioresist*).ti. OR (ablati*.ti. AND (radiotherap* OR radiation OR irradiat* OR gammaknife OR "gamma knife" OR cyberknife OR "cyber knife" OR stereotactic OR SRS OR SRT OR SBRT OR radiosurger* OR chemoradi* OR radiochemo* OR radioimmuno* OR radiosens*).ti,ab,kw.)
Toxicity	side effect/ or adverse event/ or "phase 1 clinical trial (topic)"/ or "phase 2 clinical trial (topic)"/ or "phase 3 clinical trial (topic)"/ or exp phase 1 clinical trial/ or exp phase 2 clinical trial/ or exp phase 3 clinical trial/ or (toxicit* or feasibility or "side effect*" or "adverse effect*" or "adverse event*" or DLT or "dose limiting" or "maximum tolerated dose" or morbidity or safety or safe or safely or risk or tolerance or injury or complication* or fatigue or radionecrosis or necrosis or hemorrhag* or haemorrhag* or bleeding or effusion or pain or fracture or osteoradionecrosis or perforation or ulcer or ulceration or fistula or fibrosis or stenosis or obstruction or atrophy or induration or mucositis or edema or oedema or rash or dermatitis or erythema or folliculitis or FCP or "cutis verticis gyrata" or CVG or myelitis or myelopathy or neurotoxicity or neurotoxic or neuropathy or neurocognit* or cognit* or "memory loss" or Lhermitte or encephalopathy or alopecia or xerostomia or stomatitis or hemoptysis or haemoptysis or pneumonitis or cough or dyspnoea or dyspnea or esophagitis or oesophagitis or dysphagia or nausea or vomiting or hematemesis or haematemesis or hepatitis or cardiotoxic* or "renal failure" or atherosclerosis or nephropathy or nephrotoxic* or cystitis or hematuria or haematuria or incontinence or colitis or enteritis or diarrhea or diarrhoea or proctitis or "radiation recall" or otitis or epidermolysis or "phase 1*" or "phase i" or "phase ia*" or "phase ib*" or "phase 2*" or "phase ii" or "phase iia*" or "phase iib*" or "phase 3*" or "phase iii").ti,ab,kw.
MEK	exp mitogen activated protein kinase kinase inhibitor/ OR ((MEK OR "mitogen-activated protein kinase*" OR MEK1 OR MEK2 OR MAP OR MAPKK*).ti,ab,kw. ADJ4 (inhibit* OR anti).ti,ab,kw.) OR (MEKi).ti,ab,kw. OR (trametinib OR mekinist OR cobimetinib OR cotellic OR binimetinib OR mektovi).ti,ab.
Final search	Records containing Radiotherapy *and* Toxicity *and* MEK search terms

Scopus

Topic	Search strategy
Radiotherapy	TITLE(radiotherap* OR radiation OR irradiat* OR gammaknife OR "gamma knife" OR cyberknife OR "cyber knife" OR stereotactic OR SRS OR SRT OR SBRT OR radiosurger* OR chemoradi* OR radiochemo* OR radioimmuno* OR radiosens* OR radioresist*) OR

	(TITLE(ablati*) AND TITLE-ABS(radiotherap* OR radiation OR irradiat* OR gammaknife OR "gamma knife" OR cyberknife OR "cyber knife" OR stereotactic OR SRS OR SRT OR SBRT OR radiosurger* OR chemoradi* OR radiochemo* OR radioimmuno* OR radiosens*)) OR (TITLE(ablati*) AND AUTHKEY(radiotherap* OR radiation OR irradiat* OR gammaknife OR "gamma knife" OR cyberknife OR "cyber knife" OR stereotactic OR SRS OR SRT OR SBRT OR radiosurger* OR chemoradi* OR radiochemo* OR radioimmuno* OR radiosens*))
Toxicity	TITLE-ABS (toxicit* or feasibility or "side effect*" or "adverse effect*" or "adverse event*" or DLT or "dose limiting" or "maximum tolerated dose" or morbidity or safety or safe or safely or risk or tolerance or injury or complication* or fatigue or radionecrosis or necrosis or hemorrhag* or haemorrhag* or bleeding or effusion or pain or fracture or osteoradionecrosis or perforation or ulcer or ulceration or fistula or fibrosis or stenosis or obstruction or atrophy or induration or mucositis or edema or oedema or rash or dermatitis or erythema or folliculitis or FCP or "cutis verticis gyrata" or CVG or myelitis or myelopathy or neurotoxicity or neurotoxic or neuropathy or neurocognit* or cognit* or "memory loss" or Lhermitte or encephalopathy or alopecia or xerostomia or stomatitis or hemoptysis or haemoptysis or pneumonitis or cough or dyspnoea or dyspnea or esophagitis or oesophagitis or dysphagia or nausea or vomiting or hematemesis or haematemesis or hepatitis or cardiotoxic* or "renal failure" or atherosclerosis or nephropathy or nephrotoxic* or cystitis or hematuria or haematuria or incontinence or colitis or enteritis or diarrhea or diarrhoea or proctitis or "radiation recall" or otitis or epidermolysis or "phase 1*" OR "phase i" OR "phase ia*" OR "phase ib*" OR "phase 2*" OR "phase ii" OR "phase iia*" OR "phase iib*" OR "phase 3*" OR "phase iii") or AUTHKEY (toxicit* or feasibility or "side effect*" or "adverse effect*" or "adverse event*" or DLT or "dose limiting" or "maximum tolerated dose" or morbidity or safety or safe or safely or risk or tolerance or injury or complication* or fatigue or radionecrosis or necrosis or hemorrhag* or haemorrhag* or bleeding or effusion or pain or fracture or osteoradionecrosis or perforation or ulcer or ulceration or fistula or fibrosis or stenosis or obstruction or atrophy or induration or mucositis or edema or oedema or rash or dermatitis or erythema or folliculitis or FCP or "cutis verticis gyrata" or CVG or myelitis or myelopathy or neurotoxicity or neurotoxic or neuropathy or neurocognit* or cognit* or "memory loss" or Lhermitte or encephalopathy or alopecia or xerostomia or stomatitis or hemoptysis or haemoptysis or pneumonitis or cough or dyspnoea or dyspnea or esophagitis or oesophagitis or dysphagia or nausea or vomiting or hematemesis or haematemesis or hepatitis or cardiotoxic* or "renal failure" or atherosclerosis or nephropathy or nephrotoxic* or cystitis or hematuria or haematuria or incontinence or colitis or enteritis or

	diarrhea or diarrhoea or proctitis or "radiation recall" or otitis or epidermolysis or "phase 1*" OR "phase i" OR "phase ia*" OR "phase ib*" OR "phase 2*" OR "phase ii" OR "phase iia*" OR "phase iib*" OR "phase 3*" OR "phase iii")
MEK	TITLE-ABS((MEK OR "mitogen-activated protein kinase*" OR MEK1 OR MEK2 OR MAP OR MAPKK*) W/4 (inhibit* OR anti)) OR AUTHKEY((MEK OR "mitogen-activated protein kinase*" OR MEK1 OR MEK2 OR MAP OR MAPKK*) W/4 (inhibit* OR anti)) OR TITLE-ABS(MEKi) OR AUTHKEY(MEKi) OR TITLE-ABS(trametinib OR mekinist OR cobimetinib OR cotellic OR binimetinib OR mektovi)
Final search	Records containing Radiotherapy *and* Toxicity *and* MEK search terms

Table S5. Inclusion and exclusion criteria.

	Inclusion criteria	Exclusion criteria
**Population**	All patients	
**Interventions**	RT (external beam or brachytherapy) with concurrent systemic targeted therapy/immunotherapy (between 5 $T_{1/2}$ before RT & 2 weeks after RT)	No clear information regarding the timing of radiotherapy
		Radioisotope/radionuclide therapy
		Neutron or carbon ion radiotherapy
		Vascular brachytherapy, intravascular radiotherapy
**Outcomes**	Description and grading of treatment-related toxicity (preferably according to CTCAE)	
**Study design/ publication**	Clinical studies/reports (e.g. case reports, retrospective/prospective cohorts, phase I-IV trials, meta-analyses)	Reviews (although screened for missing papers)
		Non-peer-reviewed journals
		Study protocols
		No full text available in English
		Conference abstracts

Table S6. Levels of evidence.

Derived from the ESMO Clinical Practice Guidelines Standard Operating Procedures (adapted from the Infectious Diseases Society of America-United States Public Health Service Grading System[a])	
**I**	Evidence from at least one large randomised, controlled trial of good methodological quality (low potential for bias) or meta-analyses of well-conducted randomised trials without heterogeneity
**II**	Small randomised trials or large randomised trials with a suspicion of bias (lower methodological quality) or meta-analyses of such trials or of trials demonstrated heterogeneity
**III**	Prospective cohort studies
**IV**	Retrospective cohort studies or case-control studies
**V**	Studies without control group, case reports, expert opinions

[a] By permission of Oxford University Press on behalf of the Infectious Diseases Society of America.

Dykewicz CA. Summary of the Guidelines for Preventing Opportunistic Infections among Hematopoietic Stem Cell Transplant Recipients. Clin Infect Dis. 2001;33(2):139-144 [adapted from: Gross PA, Barrett TL, Dellinger EP, et al. Purpose of quality standards for infectious diseases. Clin Infect Dis. 1994;18(3):421].

Table S7. Delphi round one results and decisions for EGFR inhibitors.

EGFR inhibitors							
**For the combination of EGFR inhibitors with RT to the:**							
**Irradiated area**	**Type of RT**	**Recommendation**	**Agree**	**Disagree**	**Agree %**	**Comment if you disagree and add relevant (new) references if applicable:**	**Statement decision for round 2**
Skin	Low-dose palliative	Consider a major adaptation.	13	6	68,4	**COMMENT 1**: Most of the data provided compares having anti-EGFR vs non-having them in the risk of cutaneous toxicity outside RT field. This only speaks about the risk of cutaneous toxicity of anti-EGFR and not about the risk of cutaneous toxicity of the combination RT-anti-EGFR. It is expected that whatever regimen containing anti-EGFR is going to have more cutaneous toxicity that not having them.<br>**COMMENT 2**: Consider a minor/no adaptation.<br>**COMMENT 3**: toxicity is usually manageable<br>**COMMENT 4**: I think we need to consider that there is a good reason for RT, and if ST is already running I am unsure if we should really wait too long... no adaptation<br>**COMMENT 5**: risk of skin toxicity known<br>**COMMENT 6**: Since most studies included a high RT dose, this would probably be ok and I would consider a minor adaption<br>**COMMENT 7:** Given the flare in disease that can be seen with interuption of EGFR TKI and do not think this is needed for palliative doses	AMENDED STATEMENT
Skin	High-dose conventionally fractionated	Consider a major adaptation.	18	1	94,7	**COMMENT 1**: toxicity is usually manageable<br>**COMMENT 2**: risk of skin toxicity known<br>**COMMENT 3**: (even if we could have exceptions like Bonner trail in H&N with Cetuximab)	NO VOTING
Skin	High-dose stereotactic	Consider a major adaptation.	16	3	84,2	**COMMENT 1**: Consider not combining these treatments.<br>**COMMENT 2**: toxicity is usually manageable<br>**COMMENT 3**: risk of skin toxicity known<br>**COMMENT 4**: No solid warning data from literature provided that radiation dose to the skin is low	SAME STATEMENT
Brain	Low-dose palliative	Consider a minor/no adaptation.	17	2	89,5	**COMMENT 1**: Based on the presented data, I would consider a major adaptation. Thoughts: we still know little about neurocognitive decline after WBRT, but we have some data showing that this can occur earlier than originally assumed (at 3-4 months post WBRT and therefor relevant for palliative patients). I don't think we have enough evidence to know this is not increased by the combined treatment.<br>**COMMENT 2**: not sure if "low-dose palliative" is adapted for the brain. lack of prospective data, may vary according to the technique of RT (proton/photon), the irradiated fields (SRS, WBRT), the volume irradiated and the dose	SAME STATEMENT
Brain	High-dose conventionally fractionated	Consider a major adaptation.	18	1	94,7	**COMMENT 1**: lack of prospective data, may vary according to the technique of RT (proton/photon), the irradiated fields (SRS, WBRT), the volume irradiated and the dose. principle of precaution should apply until enough data have been collected within clinical trials, at least for osimertinib	NO VOTING
Brain	High-dose stereotactic	Consider a major adaptation.	15	4	78,9	**COMMENT 1**: As per reference 31 quoted there was a NSS trend towards increased tox, given that SRS is short in duration would be better to err on the side of caution<br>**COMMENT 2**: I am unsure how this conclusion was reached based on the data - no adaptation<br>**COMMENT 3**: lack of prospective data, may vary according to the technique of RT (proton/photon), the irradiated fields (SRS, WBRT), the volume irradiated and the dose. principle of precaution should apply until enough data have been collected within clinical trials, at least for osimertinib<br>**COMMENT 4**: RTOG trial with SRS and Erlotinib (Sperduto, 2016)	SAME STATEMENT
Head & neck	Low-dose palliative	Consider a major adaptation.	17	2	89,5	**COMMENT 1**: Consider a minor/no adaptation.<br>**COMMENT 2**: toxicity is usually manageable<br>**COMMENT 3**: risk of mucositis known<br>**COMMENT 4**: consider previous surgical interventions & co-morbidities	SAME STATEMENT
Head & neck	High-dose conventionally fractionated	Consider a major adaptation.	16	3	84,2	**COMMENT 1**: I agree with higher rates of G3-4 mucositis and rash and inferiority of Cetuximab +RT to cisplatin + RT regiment , however concomitatnt treatment with Cetuximab and RT is still standard of care in many countries, for patients not eligible for cisplatin, when alternative is radiotherapy alone. Cetuximab is also recommended, under special circumstances, by NCCN. Until we replace Cetuximab with new drugs it's stil valid opition and it would be difficult to decrease upfront the therapeutic dose.<br>**COMMENT 2**: we have the option of adding EGFR treatment to H&N RT - this is considered a standard option by some - it is difficult to argue that this should be adapated... this should be discussed<br>**COMMENT 3**: toxicity is usually manageable<br>**COMMENT 4**: risk of mucositis known<br>**COMMENT 5**: consider previous surgical interventions & co-morbidities	SAME STATEMENT
Head & neck	High-dose stereotactic	Consider a major adaptation.	18	1	94,7	**COMMENT 1**: toxicity is usually manageable<br>**COMMENT 2**: risk of mucositis known<br>**COMMENT 3**: consider previous surgical interventions & co-morbidities	NO VOTING

Thorax	Low-dose palliative	Consider a major adaptation.	15	4	78,9	**COMMENT 1**: Consider a minor/no adaptation.<br>**COMMENT 2**: we do want to keep the pneumonitis risk low - but I think this is associated with the dose to lungs as well - I think we need to consider cases of mediastinal lymph nodes with minimal dose to the lungs - should we delay treatment - to be discussed<br>**COMMENT 3**: toxicity is usually manageable<br>**COMMENT 4**: risk of pneumonitis known<br>**COMMENT 5**: I think we need to distinguish between cetuximab and EGFR TKI. For cetuximab yes	SAME STATEMENT
Thorax	High-dose conventionally fractionated	Consider a major adaptation.	18	1	94,7	**COMMENT 1**: toxicity is usually manageable<br>**COMMENT 2**: risk of pneumonitis known	NO VOTING
Thorax	High-dose stereotactic	Consider a major adaptation.	18	1	94,7	**COMMENT 1**: no info so, consider not combining<br>**COMMENT 2**: toxicity is usually manageable<br>**COMMENT 3**: risk of pneumonitis known	NO VOTING
Abdomen/pelvis	Low-dose palliative	Consider a minor/no adaptation.	17	2	89,5	**COMMENT 1**: Based on the presented data, I would consider a major adaptation for EGFR-TKI's (not Ab's). None of the TKI's can be as specific for just EGFR as the Ab's (so always a risk of VEGF inhibition?). Especially for high dose per fraction- palliative RT, I would not feel confident that we have enough TKI data to be sure we don't cause GI perforations<br>**COMMENT 2**: unclear if the risk of toxicity is related to the dose	SAME STATEMENT
Abdomen/pelvis	High-dose conventionally fractionated	Consider a major adaptation.	16	3	84,2	**COMMENT 1**: Toxicity of the combination is acceptable<br>**COMMENT 2**: toxicity is usually manageable<br>**COMMENT 3**: risk of GI toxicity known<br>**COMMENT 4**: I would probably consider a minor adaption here given that Cetuximab is used in palliative systemic treatment as maintenance therapy in colorectal cancer it would be difficult to stop or pause. Since the studies all used high doses then a short palliative pelvic dose with IMRT would probably be acceptable.	SAME STATEMENT
Abdomen/pelvis	High-dose stereotactic	Consider a major adaptation.	16	3	84,2	**COMMENT 1**: Toxicity of the combination is acceptable<br>**COMMENT 2**: similar to the question mark above - the dose to bowel is relevant here - if irradiate a paraaortic lymph node without significant bowel dose I see no reason to adapt<br>**COMMENT 3**: toxicity is usually manageable<br>**COMMENT 4**: risk of GI toxicity known	SAME STATEMENT
Musculoskeletal tissues	Low-dose palliative	Consider a minor/no adaptation.	18	1	94,7	**COMMENT 1**: lack of prospective data	NO VOTING
Musculoskeletal tissues	High-dose conventionally fractionated	Consider a major adaptation.	17	2	89,5	**COMMENT 1**: No data to support an adaption<br>**COMMENT 2**: if we are considering high-volume high-dose - in the setting of poor evidence, caution is ok<br>**COMMENT 3**: Consider a minor/no adaptation.<br>**COMMENT 4**: lack of prospective data<br>**COMMENT 5**: This would make sense based on the principles gleaned from the other sites, but its hard to see how this would occur in clinical practice. Perhaps a synchronous tumours like a soft tissue sarcoma?	SAME STATEMENT
Musculoskeletal tissues	High-dose stereotactic	Consider a major adaptation.	16	3	84,2	**COMMENT 1**: No data to support an adaption<br>**COMMENT 2**: I would not be worried about small volume SBRT to muscle or bone<br>**COMMENT 3**: Consider a minor/no adaptation.<br>**COMMENT 4**: This would make sense based on the principles gleaned from the other sites, but its hard to see how this would occur in clinical practice. Perhaps a synchronous tumours like a soft tissue sarcoma?<br>**COMMENT 5**: lack of prospective data	SAME STATEMENT
**EXCEPTIONS: For the combination of osimertinib with RT to the:**							
Thorax	High-dose conventionally fractionated	**Consider not combining these treatments.**	18	1	94,7	**COMMENT 1**: Only for osimertinib OR for everything but the Ab's and erlotinib???<br>**COMMENT 2**: I think major adaptation is appropriate	NO VOTING
Thorax	High-dose stereotactic	**Consider not combining these treatments.**	18	1	94,7	**COMMENT 1**: I think major adaptation is appropriate	NO VOTING

Table S8. Delphi round two results for EGFR inhibitors.

EGFR inhibitors											
For the combination of EGFR inhibitors with RT to the:											
ROUND 1 STATEMENTS			LEVELS OF EVIDENCE	ROUND 1 VOTING RESULTS			ROUND 2 STATEMENTS				
Irradiated area	Type of RT	Recommendation	Level of evidence	Agree	Disagree	Agree %	Recommendation	Agree	Disagree	% Agree	Comments
Skin	Low-dose palliative	Consider a major adaptation.	1*	13	6	68,4	Consider a minor/no adaptation.	17	2	89,5	
Skin	High-dose conventionally fractionated	Consider a major adaptation.	1	18	1	94,7	STATEMENT ACCEPTED				
Skin	High-dose stereotactic	Consider a major adaptation.	2	16	3	84,2	Consider a major adaptation.	19	0	100,0	
Brain	Low-dose palliative	Consider a minor/no adaptation.	2	17	2	89,5	Consider a minor/no adaptation.	17	2	89,5	**COMMENT 1**: Osimertinib + brain RT may have increased rates of leukoencephalopathy (restrospective data suggested, PMID: 34865626
Brain	High-dose conventionally fractionated	Consider a major adaptation.	3	18	1	94,7	STATEMENT ACCEPTED				
Brain	High-dose stereotactic	Consider a major adaptation.	2	15	4	78,9	Consider a major adaptation.	19	0	100,0	
Head & neck	Low-dose palliative	Consider a major adaptation.	1*	17	2	89,5	Consider a major adaptation.	19	0	100,0	
Head & neck	High-dose conventionally fractionated	Consider a major adaptation.	1	16	3	84,2	Consider a major adaptation. **	19	0	100,0	
Head & neck	High-dose stereotactic	Consider a major adaptation.	5	18	1	94,7	STATEMENT ACCEPTED				
Thorax	Low-dose palliative	Consider a major adaptation.	1*	15	4	78,9	Consider a major adaptation.	17	2	89,5	**COMMENT 1:** Consider a minor/no adaptation. **COMMENT 2**: For single 8gy or 20/5 etc I do not think this is needed based on evidence
Thorax	High-dose conventionally fractionated	Consider a major adaptation.	1	18	1	94,7	STATEMENT ACCEPTED				
Thorax	High-dose stereotactic	Consider a major adaptation.	3	18	1	94,7	STATEMENT ACCEPTED				
Abdomen/pelvis	Low-dose palliative	Consider a minor/no adaptation.	2*	17	2	89,5	Consider a minor/no adaptation.	18	1	94,7	
Abdomen/pelvis	High-dose conventionally fractionated	Consider a major adaptation.	2	16	3	84,2	Consider a major adaptation.	19	0	100,0	
Abdomen/pelvis	High-dose stereotactic	Consider a major adaptation.	5	16	3	84,2	Consider a major adaptation.	19	0	100,0	
Musculoskeletal tissues	Low-dose palliative	Consider a minor/no adaptation.	1*	18	1	94,7	STATEMENT ACCEPTED				
Musculoskeletal tissues	High-dose conventionally fractionated	Consider a major adaptation.	1	17	2	89,5	Consider a major adaptation.	19	0	100,0	
Musculoskeletal tissues	High-dose stereotactic	Consider a major adaptation.	5	16	3	84,2	Consider a major adaptation.	19	0	100,0	
EXCEPTIONS: For the combination of osimertinib with RT to the:											
Thorax	High-dose conventionally fractionated	Consider not combining these treatments.	5	18	1	94,7	STATEMENT ACCEPTED				
Thorax	High-dose stereotactic	Consider not combining these treatments.	5	18	1	94,7	STATEMENT ACCEPTED				

* Level of evidence based on data from high RT dose schedules.

** This does not apply to intentional concurrent RT with cetuximab.

Table S9. Delphi round one results and decisions for ALK inhibitors.

**ALK inhibitors**								
**For the combination of ALK inhibitors with RT to the:**								
**Irradiated area**	**Type of RT**	**Recommendation**	**Agree**	**Disagree**	**Agree %**	**Comment if you disagree and add relevant (new) references if applicable:**	**Statement decision for round 2**
Skin	Low-dose palliative	Consider a minor/no adaptation.	18	1	94,7	**COMMENT 1**: lack of prospective data, in palliative setting, rationale for combined treatment needs to be confirmed and discussed with the patient	NO VOTING
Skin	High-dose conventionally fractionated	Consider a major adaptation.	18	1	94,7	**COMMENT 1**: No data to suggest adaptation<br>**COMMENT 2**: lack of prospective data<br>**COMMENT 3**: This comment is valid for all these different rows: no robust data from literature. Caution and safety are mandatory of course, but somewhen in our clinical activity we don't consider major adaptations with these drugs	NO VOTING
Skin	High-dose stereotactic	Consider a major adaptation.	18	1	94,7	**COMMENT 1**: No data to suggest adaptation<br>**COMMENT 2**: lack of prospective data	NO VOTING
Brain	Low-dose palliative	Consider a major adaptation.	16	3	84,2	**COMMENT 1**: No data to suggest adaptation<br>**COMMENT 2**: Consider a minor/no adaptation.<br>**COMMENT 3**: not sure if "low-dose palliative" is adapted for the brain. lack of prospective data, may vary according to the technique of RT (proton/photon), the irradiated fields (SRS, WBRT), the volume irradiated and the dose<br>**COMMENT 4**: Especially with new agents that cross the blood brain barrier	SAME STATEMENT
Brain	High-dose conventionally fractionated	Consider a major adaptation.	17	2	89,5	**COMMENT 1**: No data to suggest adaptation<br>**COMMENT 2**: lack of prospective data, may vary according to the technique of RT (proton/photon), the irradiated fields (SRS, WBRT), the volume irradiated and the dose<br>**COMMENT 3**: Especially with new agents that cross the blood brain barrier<br>**COMMENT 4:** I would choose "Consider not combining" because ALK inhibitors, and especially the newer ones (ie Lorlatinib) , have significant impact in cognition	SAME STATEMENT
Brain	High-dose stereotactic	Consider a major adaptation.	14	5	73,7	**COMMENT 1**: No data to suggest adaptation<br>**COMMENT 2**: based on the case reports more caution is needed for the two drugs mentioned<br>**COMMENT 3**: this is worth a discussion, I think clinical practice shows this is not a problem<br>**COMMENT 4**: Consider not combining these treatments for newer TKIs<br>**COMMENT 5**: lack of prospective data, may vary according to the technique of RT (proton/photon), the irradiated fields (SRS, WBRT), the volume irradiated and the dose<br>**COMMENT 6**: Especially with new agents that cross the blood brain barrier<br>**COMMENT 7:** I would choose "Consider not combining" because ALK inhibitors, and especially the newer ones (ie Lorlatinib) , have significant impact in cognition	SAME STATEMENT
Head & neck	Low-dose palliative	Consider a major adaptation.	14	5	73,7	**COMMENT 1**: No data to suggest adaptation<br>**COMMENT 2**: Consider a minor/no adaptation.<br>**COMMENT 3**: I think we have limited data for this, I am afraid we would cause undertreatment<br>**COMMENT 4**: lack of prospective data<br>**COMMENT 5**: I think a minor adaptation would be appropriate for all apart from crizotinib	SAME STATEMENT
Head & neck	High-dose conventionally fractionated	Consider a major adaptation.	18	1	94,7	**COMMENT 1**: No data to suggest adaptation<br>**COMMENT 2**: lack of prospective data	NO VOTING
Head & neck	High-dose stereotactic	Consider a major adaptation.	18	1	94,7	**COMMENT 1**: No data to suggest adaptation<br>**COMMENT 2**: lack of prospective data	NO VOTING
Thorax	Low-dose palliative	Consider a major adaptation.	14	4	77,8	**COMMENT 1**: No data to suggest adaptation<br>**COMMENT 2**: Consider a minor/no adaptation.<br>**COMMENT 3**: I think we have limited data for this, I am afraid we would cause undertreatment<br>**COMMENT 4**: lack of prospective data<br>**COMMENT 5**: I think a minor adaptation would be appropriate for all apart from crizotinib	SAME STATEMENT
Thorax	High-dose conventionally fractionated	Consider a major adaptation.	18	1	94,7	**COMMENT 1**: No data to suggest adaptation<br>**COMMENT 2**: lack of prospective data	NO VOTING
Thorax	High-dose stereotactic	Consider a major adaptation.	17	1	94,4	**COMMENT 1**: No data to suggest adaptation<br>**COMMENT 2:** I think we have limited data for this, I am afraid we would cause undertreatment<br>**COMMENT 3:** lack of prospective data	NO VOTING

Abdomen/pelvis	Low-dose palliative	Consider a major adaptation.	14	5	73,7	**COMMENT 1**: No data to suggest adaptation<br>**COMMENT 2**: would have thought it would be safe to consider minor no adaptation here- although acknowledge no data- but low dose pall RT is usually very end stage and for symptom control- bit equally acknowledge that in this setting cessation of Alk I is unlikely to do anything<br>**COMMENT 3**: Consider a minor/no adaptation.<br>**COMMENT 4**: lack of prospective data<br>**COMMENT 5**: I think a minor adaptation would be appropriate for all apart from crizotinib<br>**COMMENT 6**: I would choose "Consider a minor/no adaptation" based on literature data provided	SAME STATEMENT
Abdomen/pelvis	High-dose conventionally fractionated	Consider a major adaptation.	18	1	94,7	**COMMENT 1**: No data to suggest adaptation<br>**COMMENT 2**: lack of prospective data	NO VOTING
Abdomen/pelvis	High-dose stereotactic	Consider a major adaptation.	18	1	94,7	**COMMENT 1**: No data to suggest adaptation<br>**COMMENT 2**: lack of prospective data	NO VOTING
Musculoskeletal tissues	Low-dose palliative	Consider a minor/no adaptation.	18	1	94,7	**COMMENT 1**: lack of prospective data, in palliative setting, rationale for combined treatment needs to be confirmed and discussed with the patient	NO VOTING
Musculoskeletal tissues	High-dose conventionally fractionated	Consider a major adaptation.	17	2	89,5	**COMMENT 1**: No data to suggest adaptation<br>**COMMENT 2**: not enough info to think otherwise<br>**COMMENT 3**: lack of prospective data	SAME STATEMENT
Musculoskeletal tissues	High-dose stereotactic	Consider a major adaptation.	17	2	89,5	**COMMENT 1**: No data to suggest adaptation<br>**COMMENT 2**: I would not be worried about small volume SBRT to muscle or bone<br>**COMMENT 3**: not enough info to think otherwise<br>**COMMENT 4**: lack of prospective data	SAME STATEMENT

Table S10. Delphi round two results for ALK inhibitors.

ALK inhibitors											
For the combination of ALK inhibitors with RT to the:											
ROUND 1 STATEMENTS			LEVELS OF EVIDENCE	ROUND 1 VOTING RESULTS			ROUND 2 STATEMENTS				
Irradiated area	Type of RT	Recommendation	Level of evidence	Agree	Disagree	Agree %	Recommendation	Agree	Disagree	% Agree	Comments
Skin	Low-dose palliative	Consider a minor/no adaptation.	5	18	1	94,7	STATEMENT ACCEPTED				
Skin	High-dose conventionally fractionated	Consider a major adaptation.	5	18	1	94,7	STATEMENT ACCEPTED				
Skin	High-dose stereotactic	Consider a major adaptation.	5	18	1	94,7	STATEMENT ACCEPTED				
Brain	Low-dose palliative	Consider a major adaptation.	5	16	3	84,2	Consider a major	19	0	100,0	
Brain	High-dose conventionally fractionated	Consider a major adaptation.	5	17	2	89,5	Consider a major	19	0	100,0	
Brain	High-dose stereotactic	Consider a major adaptation.	4	14	5	73,7	Consider a major	18	1	94,7	**COMMENT 1**: Consider not combining these treatments.
Head & neck	Low-dose palliative	Consider a major adaptation.	5	14	5	73,7	Consider a major adaptation.	16	3	84,2	**COMMENT 1**: Level of evidence for ALK inhibitors is very low (5) and there is the risk of reducing drastically the dose of an active drug for a low dose palliative radiation. I would suggest minor/no adaptation<br>**COMMENT 2**: Consider a minor/no adaptation<br>**COMMENT 3**: Based on evidence I do not think this is needed
Head & neck	High-dose conventionally fractionated	Consider a major adaptation.	5	18	1	94,7	STATEMENT ACCEPTED				
Head & neck	High-dose stereotactic	Consider a major adaptation.	5	18	1	94,7	STATEMENT ACCEPTED				
Thorax	Low-dose palliative	Consider a major adaptation.	5	14	4	77,8	Consider a major adaptation.	16	2	88,9	**COMMENT 1**: Level of evidence for ALK inhibitors is very low (5) and there is the risk of reducing drastically the dose of an active drug for a low dose palliative radiation. I would suggest minor/no adaptation<br>**COMMENT 2**: Based on evidence I do not think this is needed
Thorax	High-dose conventionally fractionated	Consider a major adaptation.	5	18	1	94,7	STATEMENT ACCEPTED				
Thorax	High-dose stereotactic	Consider a major adaptation.	5	17	1	94,4	STATEMENT ACCEPTED				
Abdomen/pelvis	Low-dose palliative	Consider a major adaptation.	5	14	5	73,7	Consider a major adaptation.	16	3	84,2	**COMMENT 1**: Level of evidence for ALK inhibitors is very low (5) and there is the risk of reducing drastically the dose of an active drug for a low dose palliative radiation. I would suggest minor/no adaptation<br>**COMMENT 2**: Based on evidence I do not think this is needed
Abdomen/pelvis	High-dose conventionally fractionated	Consider a major adaptation.	5	18	1	94,7	STATEMENT ACCEPTED				
Abdomen/pelvis	High-dose stereotactic	Consider a major adaptation.	5	18	1	94,7	STATEMENT ACCEPTED				
Musculoskeletal tissues	Low-dose palliative	Consider a minor/no adaptation.	5	18	1	94,7	STATEMENT ACCEPTED				
Musculoskeletal tissues	High-dose conventionally fractionated	Consider a major adaptation.	5	17	2	89,5	Consider a major	19	0	100,0	
Musculoskeletal tissues	High-dose stereotactic	Consider a major adaptation.	5	17	2	89,5	Consider a major	19	0	100,0	

Table S11. Delphi round one results and decisions for BRAF/MEK inhibitors.

Irradiated area	Type of RT	Recommendation	Agree	Disagree	Agree %	Comment if you disagree and add relevant (new) references if applicable:	Statement decision for round 2
**BRAF/MEK inhibitors**							
**For the combination of BRAF/MEK inhibitors with RT to the:**							
Skin	Low-dose palliative	**Consider a major adaptation.**	19	0	100,0		NO VOTING
Skin	High-dose conventionally fractionated	**Consider not combining these treatments.**	19	0	100,0		NO VOTING
Skin	High-dose stereotactic	**Consider not combining these treatments.**	18	1	94,7	**COMMENT 1**: or major adaptation if skin dose low	NO VOTING
Brain	Low-dose palliative	**Consider a major adaptation.**	19	0	100,0	**COMMENT 1**: not sure if "low-dose palliative" is adapted for the brain. lack of prospective data, may vary according to the technique of RT (proton/photon), the irradiated fields (SRS, WBRT), the volume irradiated and the dose	NO VOTING
Brain	High-dose conventionally fractionated	**Consider a major adaptation.**	19	0	100,0	**COMMENT 1**: lack of prospective data, may vary according to the technique of RT (proton/photon), the irradiated fields (SRS, WBRT), the volume irradiated and the dose	NO VOTING
Brain	High-dose stereotactic	**Consider a major adaptation.**	19	0	100,0	**COMMENT 1**: lack of prospective data, may vary according to the technique of RT (proton/photon), the irradiated fields (SRS, WBRT), the volume irradiated and the dose	NO VOTING
Head & neck	Low-dose palliative	**Consider a major adaptation.**	19	0	100,0	**COMMENT 1**: lack of prospective data	NO VOTING
Head & neck	High-dose conventionally fractionated	**Consider a major adaptation.**	19	0	100,0	**COMMENT 1**: lack of prospective data	NO VOTING
Head & neck	High-dose stereotactic	**Consider a major adaptation.**	19	0	100,0	**COMMENT 1**: lack of prospective data	NO VOTING
Thorax	Low-dose palliative	**Consider a major adaptation.**	19	0	100,0	**COMMENT 1**: lack of prospective data	NO VOTING
Thorax	High-dose conventionally fractionated	**Consider a major adaptation.**	19	0	100,0	**COMMENT 1**: lack of prospective data	NO VOTING
Thorax	High-dose stereotactic	**Consider a major adaptation.**	19	0	100,0	**COMMENT 1**: lack of prospective data	NO VOTING
Abdomen/pelvis	Low-dose palliative	**Consider a major adaptation.**	19	0	100,0	**COMMENT 1**: lack of prospective data	NO VOTING
Abdomen/pelvis	High-dose conventionally fractionated	**Consider a major adaptation.**	19	0	100,0	**COMMENT 1**: lack of prospective data	NO VOTING
Abdomen/pelvis	High-dose stereotactic	**Consider a major adaptation.**	19	0	100,0	**COMMENT 1**: lack of prospective data	NO VOTING
Musculoskeletal tissues	Low-dose palliative	**Consider a major adaptation.**	16	3	84,2	**COMMENT 1**: would try to avoid being over-cautious<br>**COMMENT 2**: seems safe<br>**COMMENT 3**: lack of prospective data	SAME STATEMENT
Musculoskeletal tissues	High-dose conventionally fractionated	**Consider a major adaptation.**	19	0	100,0	**COMMENT 1**: lack of prospective data	NO VOTING
Musculoskeletal tissues	High-dose stereotactic	**Consider a major adaptation.**	19	0	100,0	**COMMENT 1**: lack of prospective data	NO VOTING
**EXCEPTION: For the combination of vemurafenib with RT to the:**							
Skin	Low-dose palliative	**Consider not combining these treatments.**	19	0	100,0	**COMMENT 1**: this should be expanded to higher doses and sbrt - or the necessity of treatment needs to be discussed more precisely	NO VOTING

Table S12. Delphi round two results for BRAF/MEK inhibitors.

BRAF/MEK inhibitors												
For the combination of BRAF/MEK inhibitors with RT to the:												
ROUND 1 STATEMENTS			LEVELS OF EVIDENCE	ROUND 1 VOTING RESULTS			ROUND 2 STATEMENTS	ROUND 2 VOTING AREA				
Irradiated area	Type of RT	Recommendation	Level of evidence	Agree	Disagree	Agree %	Recommendation	Agree	Disagree	% Agree	Comments	
Skin	Low-dose palliative	Consider a major adaptation.	4	19	0	100,0	STATEMENT ACCEPTED			100,0		
Skin	High-dose conventionally fractionated	Consider not combining these treatments.	4	19	0	100,0	STATEMENT ACCEPTED			100,0		
Skin	High-dose stereotactic	Consider not combining these treatments.	4	18	1	94,7	STATEMENT ACCEPTED			94,7		
Brain	Low-dose palliative	Consider a major adaptation.	4	19	0	100,0	STATEMENT ACCEPTED			100,0		
Brain	High-dose conventionally fractionated	Consider a major adaptation.	4	19	0	100,0	STATEMENT ACCEPTED			100,0		
Brain	High-dose stereotactic	Consider a major adaptation.	4	19	0	100,0	STATEMENT ACCEPTED			100,0		
Head & neck	Low-dose palliative	Consider a major adaptation.	5	19	0	100,0	STATEMENT ACCEPTED			100,0		
Head & neck	High-dose conventionally fractionated	Consider a major adaptation.	5	19	0	100,0	STATEMENT ACCEPTED			100,0		
Head & neck	High-dose stereotactic	Consider a major adaptation.	5	19	0	100,0	STATEMENT ACCEPTED			100,0		
Thorax	Low-dose palliative	Consider a major adaptation.	5	19	0	100,0	STATEMENT ACCEPTED			100,0		
Thorax	High-dose conventionally fractionated	Consider a major adaptation.	5	19	0	100,0	STATEMENT ACCEPTED			100,0		
Thorax	High-dose stereotactic	Consider a major adaptation.	5	19	0	100,0	STATEMENT ACCEPTED			100,0		
Abdomen/pelvis	Low-dose palliative	Consider a major adaptation.	5	19	0	100,0	STATEMENT ACCEPTED			100,0		
Abdomen/pelvis	High-dose conventionally fractionated	Consider a major adaptation.	3	19	0	100,0	STATEMENT ACCEPTED			100,0		
Abdomen/pelvis	High-dose stereotactic	Consider a major adaptation.	5	19	0	100,0	STATEMENT ACCEPTED			100,0		
Musculoskeletal tissue	Low-dose palliative	Consider a major adaptation.	4	16	3	84,2	Consider a major adaptation.	18	1	94,7	**COMMENT 1:** Consider a minor/no adaptation	
Musculoskeletal tissue	High-dose conventionally fractionated	Consider a major adaptation.	5	19	0	100,0	STATEMENT ACCEPTED			100,0		
Musculoskeletal tissue	High-dose stereotactic	Consider a major adaptation.	5	19	0	100,0	STATEMENT ACCEPTED			100,0		
**EXCEPTION: For the combination of vemurafenib with RT to the:**												
Skin	Low-dose palliative	Consider not combining these treatments.	4	19	0	100,0	STATEMENT ACCEPTED			100,0		
